# Supplementary material for: The Modified Shields Classification and 12 Families with Defined DSPP Mutations
Source: Genes (Basel). 2022 May 12;13(5):858. doi: 10.3390/genes13050858 (PMC9141616; doi:10.3390/genes13050858)
Supplement: Supplementary file 1 [file genes-13-00858-s001.zip › genes-1682702-supplementary.pdf]

The Modified Shields Classification and 12 Families with Defined *DSPP* Mutations

James P. Simmer[1*], Hong Zhang[1], Sophie J.H. Moon[1], Lori A-J. Donnelly[2], Yuan-Ling Lee[3], Figen Seymen[4],Mine Koruyucu[5], Hui-Chen Chan[6], Kevin Y. Lee[6], Suwei Wu[6], Chia-Lan Hsiang[6], Anthony T.P. Tsai[6], Rebecca L. Slayton[7], Melissa Morrow[8], Shih-Kai Wang[9], Edward D. Shields[10], and Jan C.-C. Hu[1]

1. Biologic and Materials Sciences & Prosthodontics, University of Michigan School of Dentistry, 1011 N University Ave. Ann Arbor, Michigan, USA.
2. Department of Oral and Maxillofacial Surgery, Adams School of Dentistry, University of North Carolina at Chapel Hill, 149 Brauer Hall, CB #7450 Chapel Hill, NC 27599.
3. Graduate Institute of Clinical Dentistry, National Taiwan University, No.1, Chang-de St., Zhongzheng Dist., Taipei City 100, Taiwan R.O.C.
4. Department of Pedodontics, Faculty of Dentistry, Altinbas University, Istanbul, Turkey
5. Department of Pedodontics, Faculty of Dentistry, Istanbul University, Istanbul, Turkey.
6. Taipei Municipal WanFang Hospital, Xinglong Rd 111, Taipei, Taiwan.
7. Department of Pediatric Dentistry, University of Washington School of Dentistry. 1959 NE Pacific St., B-307 Seattle, WA 98195-6365.
8. Department of Orthodontic and Pediatric Dentistry, University of Michigan School of Dentistry, 1011 N University Ave. Ann Arbor, Michigan, USA.
9. Department of Dentistry, National Taiwan University School of Dentistry, No. 1, Changde St., Zhongzheng Dist., Taipei City 100, Taiwan.
10. 2161 Pardee Road, Neebing, Ontario, CA P7L0G7

Supplemental Data File

Table S1. Alignment of DPP coding region of *DSPP* showing allelic variants and disease-causing mutations

Table S1. Alignment of DPP coding region of *DSPP* showing allelic variants and disease-causing mutations. DPP sequences exhibit novel patterns of insertions and deletions (indels), necessitating the use of this type of alignment to propertly compare patient data to the DSPP reference sequencer ("REFSEQ" is the NCBI *DSPP* reference sequence NM_014208.3). "Fam8a, Fam8b, Fam9a, Fam9b, Fam10a, Fam10b, Fam12a and Fam12b" are DPP sequences determined by SMART sequencing that show the mutated and native alleles of an affected member from Families 8 through 11 in this study. Ten DPP sequences (F1A through F5B) are from the 5 patients with inherited dentin defects previously characterized by SMART sequencing [1] Wild-type DPP haplotypes were downloaded from NCBI PopSets 162077127 and 162077085 [2]. These PopSets contain DPP haplotype sequences from 2 different sizes of cloned DPP polymerase chain reaction products [3] and are labeled "HAP#". Haplotypes labeled "SHAP#" were characterized in China and published, but were not submitted to GenBank [4]. However, the locations of all indels were clearly described with respect to the reference sequence. The alignments were made manually, trying to minimize the number of genetic events that would have been required to generate the observed allelic differences in DPP sequences. Only a single previously published DPP sequence for each indel pattern was retained in the final alignment. "MERGED" is a hypothetical *DSPP* haplotype that contains the sequences of all indels in the alignment, while "TRANSL" is the translation of the "MERGED" sequence. Notes provide the locations and phenotypes of mutations associated with inherited dentin defects. Blue highlight indicates a mutation that was manifested as type II dentin dysplasia. Green highlight indicates a mutation that was manifested as dentinogenesis imperfecta. Numbering of the mutation positions was based on the *DSPP* gene reference sequence NG_012151.1, starting with nucleotide 1, the *DSPP* mRNA reference sequence NM_004771.3, starting from the A of the ATG translation initiation codon, and the protein reference sequence NP_055023.2, starting with the Met[1] at the begining of the signal peptide sequence. The nomenclature used for all disease-causing *DSPP* sequence variations were verified using Mutalyzer 2.0.32 at https://mutalyzer.nl/

```
                                                                                                                                                                                                                            1572
REFSEQ                        (1387) GATGATCCCAATAGCAGTGATGAATCTAATGGCAATGATGATGCTAATTCAGAAAGTGACAATAACAGCAGTAGCCGAGGAGATGCTTCTTATAACTCTGATGAATCAAAAGATAATGGCAATGGCAGTGACTCAAAAGGAGCAGAAGATGATGACAGTGATAGCACATCAGACACTAATAATAGT
Fam8a                       CATGCAAGGAGATGATCCCAATAGCAGTGATGAATCTAATGGCAATGATGATGCTAATTCAGAAAGTGACAATAACAGCAGTAGCCGAGGAGATGCTTCTTATAACTCTGATGAATCAAAAGATAATGGCAATGGCAGTGACTCAAAAGGAGCAGAAGATGATGACAGTGATAGCACATCAGACACTAATAATAGT
Fam8b                       CATGCAAGGAGATGATCCCAATAGCAGTGATGAATCTAATGGCAATGATGATGCTAATTCAGAAAGTGACAATAACAGCAGTAGCCGAGGAGATGCTTCTTATAACTCTGATGAATCAAAAGATAATGGCAATGGCAGTGACTCAAAAGGAGCAGAAGATGATGACAGTGATAGCACATCAGACACTAATAATAGT
Fam9a                       CATGCAAGGAGATGATCCCAATAGCAGTGATGAATCTAATGGCAATGATGATGCTAATTCAGAAAGTGACAATAACAGCAGTAGCCGAGGAGATGCTTCTTATAACTCTGATGAATCAAAAGATAATGGCAATGGCAGTGACTCAAAAGGAGCAGAAGATGATGACAGTGATAGCACATCAGACACTAATAATAGT
Fam9b                        ATGCAAGGAGATGATCCCAATAGCAGTGATGAATCTAATGGCAATGATGATGCTAATTCAGAAAGTGACAATAACAGCAGTAGCCGAGGAGATGCTTCTTATAACTCTGATGAATCAAAAGATAATGGCAATGGCAGTGACTCAAAAGGAGCAGAAGATGATGACAGTGATAGCACATCAGACACTAATAATAGT
Fam10a                      CATGCAAGGAGATGATCCCAATAGCAGTGATGAATCTAATGGCAATGATGATGCTAATTCAGAAAGTGACAATAACAGCAGTAGCCGAGGAGATGCTTCTTATAACTCTGATGAATCAAAAGATAATGGCAATGGCAGTGACTCAAAAGGAGCAGAAGATGATGACAGTGATAGCACATCAGACACTAATAATAGT
Fam10b                      CATGCAAGGAGATGATCCCAATAGCAGTGATGAATCTAATGGCAATGATGATGCTAATTCAGAAAGTGACAATAACAGCAGTAGCCGAGGAGATGCTTCTTATAACTCTGATGAATCAAAAGATAATGGCAATGGCAGTGACTCAAAAGGAGCAGAAGATGATGACAGTGATAGCACATCAGACACTAATAATAGT
Fam11a                      CATGCAAGGAGATGATCCCAATAGCAGTGATGAATCTAATGGCAATGATGATGCTAATTCAGAAAGTGACAATAACAGCAGTAGCCGAGGAGATGCTTCTTATAACTCTGATGAATCAAAAGATAATGGCAATGGCAGTGACTCAAAAGGAGCAGAAGATGATGACAGTGATAGCACATCAGACACTAATAATAGT
Fam11b                      CATGCAAGGAGATGATCCCAATAGCAGTGATGAATCTAATGGCAATGATGATGCTAATTCAGAAAGTGACAATAACAGCAGTAGCCGAGGAGATGCTTCTTATAACTCTGATGAATCAAAAGATAATGGCAATGGCAGTGACTCAAAAGGAGCAGAAGATGATGACAGTGATAGCACATCAGACACTAATAATAGT
Fam12a                       ATGCAAGGAGATGATCCCAATAGCAGTGATGAATCTAATGGCAATGATGATGCTAATTCAGAAAGTGACAATAACAGCAGTAGCCGAGGAGATGCTTCTTATAACTCTGATGAATCAAAAGATAATGGCAATGGCAGTGACTCAAAAGGAGCAGAAGATGATGACAGTGATAGCACATCAGACACTAATAATAGT
Fam12b                       ATGCAAGGAGATGATCCCAATAGCAGTGATGAATCTAATGGCAATGATGATGCTAATTCAGAAAGTGACAATAACAGCAGTAGCCGAGGAGATGCTTCTTATAACTCTGATGAATCAAAAGATAATGGCAATGGCAGTGACTCAAAAGGAGCAGAAGATGATGACAGTGATAGCACATCAGACACTAATAATAGT
F1A                     AGTCCATGCAAGGAGATGATCCCAATAGCAGTGATGAATCTAATGGCAATGATGATGCTAATTCAGAAAGTGACAATAACAGCAGTAGCCGAGGAGATGCTTCTTATAACTCTGATGAATCAAAAGATAATGGCAATGGCAGTGACTCAAAAGGAGCAGAAGATGATGACAGTGATAGCACATCAGACACTAATAATAGT
F1B                     AGTCCATGCAAGGAGATGATCCCAATAGCAGTGATGAATCTAATGGCAATGATGATGCTAATTCAGAAAGTGACAATAACAGCAGTAGCCGAGGAGATGCTTCTTATAACTCTGATGAATCAAAAGATAATGGCAATGGCAGTGACTCAAAAGGAGCAGAAGATGATGACAGTGATAGCACATCAGACACTAATAATAGT
F2A                     AGTCCATGCAAGGAGATGATCCCAATAGCAGTGATGAATCTAATGGCAATGATGATGCTAATTCAGAAAGTGACAATAACAGCAGTAGCCGAGGAGATGCTTCTTATAACTCTGATGAATCAAAAGATAATGGCAATGGCAGTGACTCAAAAGGAGCAGAAGATGATGACAGTGATAGCACATCAGACACTAATAATAGT
F2B                     AGTCCATGCAAGGAGATGATCCCAATAGCAGTGATGAATCTAATGGCAATGATGATGCTAATTCAGAAAGTGACAATAACAGCAGTAGCCGAGGAGATGCTTCTTATAACTCTGATGAATCAAAAGATAATGGCAATGGCAGTGACTCAAAAGGAGCAGAAGATGATGACAGTGATAGCACATCAGACACTAATAATAGT
F3A                     AGTCCATGCAAGGAGATGATCCCAATAGCAGTGATGAATCTAATGGCAATGATGATGCTAATTCAGAAAGTGACAATAACAGCAGTAGCCGAGGAGATGCTTCTTATAACTCTGATGAATCAAAAGATAATGGCAATGGCAGTGACTCAAAAGGAGCAGAAGATGATGACAGTGATAGCACATCAGACACTAATAATAGT
F3B                     AGTCCATGCAAGGAGATGATCCCAATAGCAGTGATGAATCTAATGGCAATGATGATGCTAATTCAGAAAGTGACAATAACAGCAGTAGCCGAGGAGATGCTTCTTATAACTCTGATGAATCAAAAGATAATGGCAATGGCAGTGACTCAAAAGGAGCAGAAGATGATGACAGTGATAGCACATCAGACACTAATAATAGT
F4A                     AGTCCATGCAAGGAGATGATCCCAATAGCAGTGATGAATCTAATGGCAATGATGATGCTAATTCAGAAAGTGACAATAACAGCAGTAGCCGAGGAGATGCTTCTTATAACTCTGATGAATCAAAAGATAATGGCAATGGCAGTGACTCAAAAGGAGCAGAAGATGATGACAGTGATAGCACATCAGACACTAATAATAGT
F4B                     AGTCCATGCAAGGAGATGATCCCAATAGCAGTGATGAATCTAATGGCAATGATGATGCTAATTCAGAAAGTGACAATAACAGCAGTAGCCGAGGAGATGCTTCTTATAACTCTGATGAATCAAAAGATAATGGCAATGGCAGTGACTCAAAAGGAGCAGAAGATGATGACAGTGATAGCACATCAGACACTAATAATAGT
F5A                     AGTCCATGCAAGGAGATGATCCCAATAGCAGTGATGAATCTAATGGCAATGATGATGCTAATTCAGAAAGTGACAATAACAGCAGTAGCCGAGGAGATGCTTCTTATAACTCTGATGAATCAAAAGATAATGGCAATGGCAGTGACTCAAAAGGAGCAGAAGATGATGACAGTGATAGCACATCAGACACTAATAATAGT
F5B                     AGTCCATGCAAGGAGATGATCCCAATAGCAGTGATGAATCTAATGGCAATGATGATGCTAATTCAGAAAGTGACAATAACAGCAGTAGCCGAGGAGATGCTTCTTATAACTCTGATGAATCAAAAGATAATGGCAATGGCAGTGACTCAAAAGGAGCAGAAGATGATGACAGTGATAGCACATCAGACACTAATAATAGT
HAP1A                                                                                                                 CGAGGAGATGCTTCTTATAACTCTGATGAATCAAAAGATAATGGCAATGGCAGTGACTCAAAAGGAGCAGAAGATGATGACAGTGATAGCACATCAGACACTAATAATAGT
HAP2A                                                                                                                 CGAGGAGATGCTTCTTATAACTCTGATGAATCAAAAGATAATGGCAATGGCAGTGACTCAAAAGGAGCAGAAGATGATGACAGTGATAGCACATCAGACACTAATAATAGT
HAP3A                                                                                                                 CGAGGAGATGCTTCTTATAACTCTGATGAATCAAAAGATAATGGCAATGGCAGTGACTCAAAAGGAGCAGAAGATGATGACAGTGATAGCACATCAGACACTAATAATAGT
HAP15A                                                                                                                CGAGGAGATGCTTCTTATAACTCTGATGAATCAAAAGATAATGGCAATGGCAGTGACTCAAAAGGAGCAGAAGATGATGACAGTGATAGCACATCAGACACTAATAATAGT
HAP17B                                                                                                                CGAGGAGATGCTTCTTATAACTCTGATGAATCAAAAGATAATGGCAATGGCAGTGACTCAAAAGGAGCAGAAGATGATGACAGTGATAGCACATCAGACACTAATAATAGT
HAP20A                                                                                                                CGAGGAGATGCTTCTTATAACTCTGATGAATCAAAAGATAATGGCAATGGCAGTGACTCAAAAGGAGCAGAAGATGATGACAGTGATAGCACATCAGACACTAATAATAGT
HAP20B                                                                                                                CGAGGAGATGCTTCTTATAACTCTGATGAATCAAAAGATAATGGCAATGGCAGTGACTCAAAAGGAGCAGAAGATGATGACAGTGATAGCACATCAGACACTAATAATAGT
HAP36A                                                                                                                CGAGGAGATGCTTCTTATAACTCTGATGAATCAAAAGATAATGGCAATGGCAGTGACTCAAAAGGAGCAGAAGATGATGACAGTGATAGCACATCAGACACTAATAATAGT
HAP37A                                                                                                                CGAGGAGATGCTTCTTATAACTCTGATGAATCAAAAGATAATGGCAATGGCAGTGACTCAAAAGGAGCAGAAGATGATGACAGTGATAGCACATCAGACACTAATAATAGT
HAP38A                                                                                                                CGAGGAGATGCTTCTTATAACTCTGATGAATCAAAAGATAATGGCAATGGCAGTGACTCAAAAGGAGCAGAAGATGATGACAGTGATAGCACATCAGACACTAATAATAGT
SHAP1:                                 GATGATCCCAATAGCAGTGATGAATCTAATGGCAATGATGATGCTAATTCAGAAAGTGACAATAACAGCAGTAGCCGAGGAGATGCTTCTTATAACTCTGATGAATCAAAAGATAATGGCAATGGCAGTGACTCAAAAGGAGCAGAAGATGATGACAGTGATAGCACATCAGACACTAATAATAGT
SHAP2:                                 GATGATCCCAATAGCAGTGATGAATCTAATGGCAATGATGATGCTAATTCAGAAAGTGACAATAACAGCAGTAGCCGAGGAGATGCTTCTTATAACTCTGATGAATCAAAAGATAATGGCAATGGCAGTGACTCAAAAGGAGCAGAAGATGATGACAGTGATAGCACATCAGACACTAATAATAGT
SHAP3:                                 GATGATCCCAATAGCAGTGATGAATCTAATGGCAATGATGATGCTAATTCAGAAAGTGACAATAACAGCAGTAGCCGAGGAGATGCTTCTTATAACTCTGATGAATCAAAAGATAATGGCAATGGCAGTGACTCAAAAGGAGCAGAAGATGATGACAGTGATAGCACATCAGACACTAATAATAGT
SHAP4:                                 GATGATCCCAATAGCAGTGATGAATCTAATGGCAATGATGATGCTAATTCAGAAAGTGACAATAACAGCAGTAGCCGAGGAGATGCTTCTTATAACTCTGATGAATCAAAAGATAATGGCAATGGCAGTGACTCAAAAGGAGCAGAAGATGATGACAGTGATAGCACATCAGACACTAATAATAGT
SHAP5:                                 GATGATCCCAATAGCAGTGATGAATCTAATGGCAATGATGATGCTAATTCAGAAAGTGACAATAACAGCAGTAGCCGAGGAGATGCTTCTTATAACTCTGATGAATCAAAAGATAATGGCAATGGCAGTGACTCAAAAGGAGCAGAAGATGATGACAGTGATAGCACATCAGACACTAATAATAGT
SHAP6:                                 GATGATCCCAATAGCAGTGATGAATCTAATGGCAATGATGATGCTAATTCAGAAAGTGACAATAACAGCAGTAGCCGAGGAGATGCTTCTTATAACTCTGATGAATCAAAAGATAATGGCAATGGCAGTGACTCAAAAGGAGCAGAAGATGATGACAGTGATAGCACATCAGACACTAATAATAGT
SHAP6(2)                               GATGATCCCAATAGCAGTGATGAATCTAATGGCAATGATGATGCTAATTCAGAAAGTGACAATAACAGCAGTAGCCGAGGAGATGCTTCTTATAACTCTGATGAATCAAAAGATAATGGCAATGGCAGTGACTCAAAAGGAGCAGAAGATGATGACAGTGATAGCACATCAGACACTAATAATAGT
SHAP7                                  GATGATCCCAATAGCAGTGATGAATCTAATGGCAATGATGATGCTAATTCAGAAAGTGACAATAACAGCAGTAGCCGAGGAGATGCTTCTTATAACTCTGATGAATCAAAAGATAATGGCAATGGCAGTGACTCAAAAGGAGCAGAAGATGATGACAGTGATAGCACATCAGACACTAATAATAGT
```

```
SHAP102          GATGATCCCAATAGCAGTGATGAATCTAATGGCAATGATGATGCTAATTCAGAAAGTGACAATAACAGCAGTAGCCGAGGAGATGCTTCTTATAACTCTGATGAATCAAAAGATAATGGCAATGGCAGTGACTCAAAAGGAGCAGAAGATGATGACAGTGATAGCACATCAGACACTAATAATAGT
SHAP130          GATGATCCCAATAGCAGTGATGAATCTAATGGCAATGATGATGCTAATTCAGAAAGTGACAATAACAGCAGTAGCCGAGGAGATGCTTCTTATAACTCTGATGAATCAAAAGATAATGGCAATGGCAGTGACTCAAAAGGAGCAGAAGATGATGACAGTGATAGCACATCAGACACTAATAATAGT
SHAP106          GATGATCCCAATAGCAGTGATGAATCTAATGGCAATGATGATGCTAATTCAGAAAGTGACAATAACAGCAGTAGCCGAGGAGATGCTTCTTATAACTCTGATGAATCAAAAGATAATGGCAATGGCAGTGACTCAAAAGGAGCAGAAGATGATGACAGTGATAGCACATCAGACACTAATAATAGT
SHAP72           GATGATCCCAATAGCAGTGATGAATCTAATGGCAATGATGATGCTAATTCAGAAAGTGACAATAACAGCAGTAGCCGAGGAGATGCTTCTTATAACTCTGATGAATCAAAAGATAATGGCAATGGCAGTGACTCAAAAGGAGCAGAAGATGATGACAGTGATAGCACATCAGACACTAATAATAGT
SHAP110          GATGATCCCAATAGCAGTGATGAATCTAATGGCAATGATGATGCTAATTCAGAAAGTGACAATAACAGCAGTAGCCGAGGAGATGCTTCTTATAACTCTGATGAATCAAAAGATAATGGCAATGGCAGTGACTCAAAAGGAGCAGAAGATGATGACAGTGATAGCACATCAGACACTAATAATAGT
MERGED           GATGATCCCAATAGCAGTGATGAATCTAATGGCAATGATGATGCTAATTCAGAAAGTGACAATAACAGCAGTAGCCGAGGAGATGCTTCTTATAACTCTGATGAATCAAAAGATAATGGCAATGGCAGTGACTCAAAAGGAGCAGAAGATGATGACAGTGATAGCACATCAGACACTAATAATAGT
TRANSL    (463)   D  D  P  N  S  S  D  E  S  N  G  N  D  D  A  N  S  E  S  D  N  N  S  S  S  R  G  D  A  S  Y  N  S  D  E  S  K  D  N  G  N  G  S  D  S  K  G  A  E  D  D  D  S  D  S  T  S  D  T  N  N  S   524
```

```
              1573                                                                                                                     1686                                                                                        1772
REFSEQ    GACAGTAATGGCAATGGTAACAATGGGAATGATGACAATGACAAATCAGACAGTGGCAAAGGTAAATCAGATAGCAGTGACAGTGATAGTAGTGATAGCAGCAATAGCAGTGATAGTAGTGACAGCAGTGACAGTGACAGCAGTGATAGCAACAGTAGCAGTGATAGTGACAGCAGTGACAGTGACAGCAGTGATAGCAG
Fam8a     GACAGTAATGGCAATGGTAACAATGGGAATGATGACAATGACAAATCAGACAGTGGCAAAGGTAAATCAGATAGCAGTGACAGTGATAGTAGTGATAGCAGCAATAGCAGTGATAGTAGTGACAGCAGTGACAGTGACAGCAGTGATAGCAACAGTAGCAGTGATAGTGACAGCAGTGACAGTGACAGCAGTGATAGCAG
Fam8b     GACAGTAATGGCAATGGTAACAATGGGAATGATGACAATGACAAATCAGACAGTGGCAAAGGTAAATCAGATAGCAGTGACAGTGATAGTAGTGATAGCAGCAATAGCAGTGATAGTAGTGACAGCAGTGACAGTGACAGCAGTGATAGCAACAGTAGCAGTGATAGTGACAGCAGTGACAGTGACAGCAGTGATAGCAG
Fam9a     GACAGTAATGGCAATGGTAACAATGGGAATGATGACAATGACAAATCAGACAGTGGCAAAGGTAAATCAGATAGCAGTGACAGTGATAGTAGTGATAGCAGCAATAGCAGTGATAGTAGTGACAGCAGTGACAGTGACAGCAGTGATAGCAACAGTAGCAGTGATAGTGACAGCAGTGACAGTGACAGCAGTGATAGCAG
Fam9b     GACAGTAATGGCAATGGTAACAATGGGAATGATGACAATGACAAATCAGACAGTGGCAAAGGTAAATCAGATAGCAGTGACAGTGATAGTAGTGATAGCAGCAATAGCAGTGATAGTAGTGACAGCAGTGACAGTGACAGCAGTGATAGCAACAGTAGCAGTGATAGTGACAGCAGTGACAGTGACAGCAGTGATAGCAG
Fam10a    GACAGTAATGGCAATGGTAACAATGGGAATGATGACAATGACAAATCAGACAGTGGCAAAGGTAAATCAGATAGCAGTGACAGTGATAGTAGTGATAGCAGCAATAGCAGTGATAGTAGTGACAGCAGTGACAGTGACAGCAGTGATAGCAACAGTAGCAGTGATAGTGACAGCAGTGACAGTGACAGCAGTGATAGCAG
Fam10b    GACAGTAATGGCAATGGTAACAATGGGAATGATGACAATGACAAATCAGACAGTGGCAAAGGTAAATCAGATAGCAGTGACAGTGATAGTAGTGATAGCAGCAATAGCAGTGATAGTAGTGACAGCAGTGACAGTGACAGCAGTGATAGCAACAGTAGCAGTGATAGTGACAGCAGTGACAGTGACAGCAGTGATAGCAG
Fam11a    GACAGTAATGGCAATGGTAACAATGGGAATGATGACAATGACAAATCAGACAGTGGCAAAGGTAAATCAGATAGCAGTGACAGTGATAGTAGTGATAGCAGCAATAGCAGTGATAGTAGTGACAGCAGTGACAGTGACAGCAGTGATAGCAACAGTAGCAGTGATAGTGACAGCAGTGACAGTGACAGCAGTGATAGCAG
Fam11b    GACAGTAATGGCAATGGTAACAATGGGAATGATGACAATGACAAATCAGACAGTGGCAAAGGTAAATCAGATAGCAGTGACAGTGATAGTAGTGATAGCAGCAATAGCAGTGATAGTAGTGACAGCAGTGACAGTGACAGCAGTGATAGCAACAGTAGCAGTGATAGTGACAGCAGTGACAGTGACAGCAGTGATAGCAG
Fam12a    GACAGTAATGGCAATGGTAACAATGGGAATGATGACAATGACAAATCAGACAGTGGCAAAGGTAAATCAGATAGCAGTGACAGTGATAGTAGTGATAGCAGCAATAGCAGTGATAGTAGTGACAGCAGTGACAGTGACAGCAGTGATAGCAACAGTAGCAGTGATAGTGACAGCAGTGACAGTGACAGCAGTGATAGCAG
Fam12b    GACAGTAATGGCAATGGTAACAATGGGAATGATGACAATGACAAATCAGACAGTGGCAAAGGTAAATCAGATAGCAGTGACAGTGATAGTAGTGATAGCAGCAATAGCAGTGATAGTAGTGACAGCAGTGACAGTGACAGCAGTGATAGCAACAGTAGCAGTGATAGTGACAGCAGTGACAGTGACAGCAGTGATAGCAG
F1A       GACAGTAATGGCAATGGTAACAATGGGAATGATGACAATGACAAATCAGACAGTGGCAAAGGTAAATCAGATAGCAGTGACAGTGATAGTAGTGATAGCAGCAATAGCAGTGATAGTAGTGACAGCAGTGACAGTGACAGCAGTGATAGCAACAGTAGCAGTGATAGTGACAGCAGTGACAGTGACAGCAGTGATAGCAG
F1B       GACAGTAATGGCAATGGTAACAATGGGAATGATGACAATGACAAATCAGACAGTGGCAAAGGTAAATCAGATAGCAGTGACAGTGATAGTAGTGATAGCAGCAATAGCAGTGATAGTAGTGACAGCAGTGACAGTGACAGCAGTGATAGCAACAGTAGCAGTGATAGTGACAGCAGTGACAGTGACAGCAGTGATAGCAG
F2A       GACAGTAATGGCAATGGTAACAATGGGAATGATGACAATGACAAATCAGACAGTGGCAAAGGTAAATCAGATAGCAGTGACAGTGATAGTAGTGATAGCAGCAATAGCAGTGATAGTAGTGACAGCAGTGACAGTGACAGCAGTGATAGCAACAGTAGCAGTGATAGTGACAGCAGTGACAGTGACAGCAGTGATAGCAG
F2B       GACAGTAATGGCAATGGTAACAATGGGAATGATGACAATGACAAATCAGACAGTGGCAAAGGTAAATCAGATAGCAGTGACAGTGATAGTAGTGATAGCAGCAATAGCAGTGATAGTAGTGACAGCAGTGACAGTGACAGCAGTGATAGCAACAGTAGCAGTGATAGTGACAGCAGTGACAGTGACAGCAGTGATAGCAG
F3A       GACAGTAATGGCAATGGTAACAATGGGAATGATGACAATGACAAATCAGACAGTGGCAAAGGTAAATCAGATAGCAGTGACAGTGATAGTAGTGATAGCAGCAATAGCAGTGATAGTAGTGACAGCAGTGACAGTGACAGCAGTGATAGCAACAGTAGCAGTGATAGTGACAGCAGTGACAGTGACAGCAGTGATAGCAG
F3B       GACAGTAATGGCAATGGTAACAATGGGAATGATGACAATGACAAATCAGACAGTGGCAAAGGTAAATCAGATAGCAGTGACAGTGATAGTAGTGATAGCAGCAATAGCAGTGATAGTAGTGACAGCAGTGACAGTGACAGCAGTGATAGCAACAGTAGCAGTGATAGTGACAGCAGTGACAGTGACAGCAGTGATAGCAG
F4A       GACAGTAATGGCAATGGTAACAATGGGAATGATGACAATGACAAATCAGACAGTGGCAAAGGTAAATCAGATAGCAGTGACAGTGATAGTAGTGATAGCAGCAATAGCAGTGATAGTAGTGACAGCAGTGACAGTGACAGCAGTGATAGCAACAGTAGCAGTGATAGTGACAGCAGTGACAGTGACAGCAGTGATAGCAG
F4B       GACAGTAATGGCAATGGTAACAATGGGAATGATGACAATGACAAATCAGACAGTGGCAAAGGTAAATCAGATAGCAGTGACAGTGATAGTAGTGATAGCAGCAATAGCAGTGATAGTAGTGACAGCAGTGACAGTGACAGCAGTGATAGCAACAGTAGCAGTGATAGTGACAGCAGTGACAGTGACAGCAGTGATAGCAG
F5A       GACAGTAATGGCAATGGTAACAATGGGAATGATGACAATGACAAATCAGACAGTGGCAAAGGTAAATCAGATAGCAGTGACAGTGATAGTAGTGATAGCAGCAATAGCAGTGATAGTAGTGACAGCAGTGACAGTGACAGCAGTGATAGCAACAGTAGCAGTGATAGTGACAGCAGTGACAGTGACAGCAGTGATAGCAG
F5B       GACAGTAATGGCAATGGTAACAATGGGAATGATGACAATGACAAATCAGACAGTGGCAAAGGTAAATCAGATAGCAGTGACAGTGATAGTAGTGATAGCAGCAATAGCAGTGATAGTAGTGACAGCAGTGACAGTGACAGCAGTGATAGCAACAGTAGCAGTGATAGTGACAGCAGTGACAGTGACAGCAGTGATAGCAG
HAP1A     GACAGTAATGGCAATGGTAACAATGGGAATGATGACAATGACAAATCAGACAGTGGCAAAGGTAAATCAGATAGCAGTGACAGTGATAGTAGTGATAGCAGCAATAGCAGTGATAGTAGTGACAGCAGTGACAGTGACAGCAGTGATAGCAACAGTAGCAGTGATAGTGACAGCAGTGACAGTGACAGCAGTGATAGCAG
HAP2A     GACAGTAATGGCAATGGTAACAATGGGAATGATGACAATGACAAATCAGACAGTGGCAAAGGTAAATCAGATAGCAGTGACAGTGATAGTAGTGATAGCAGCAATAGCAGTGATAGTAGTGACAGCAGTGACAGTGACAGCAGTGATAGCAACAGTAGCAGTGATAGTGACAGCAGTGACAGTGACAGCAGTGATAGCAG
HAP3A     GACAGTAATGGCAATGGTAACAATGGGAATGATGACAATGACAAATCAGACAGTGGCAAAGGTAAATCAGATAGCAGTGACAGTGATAGTAGTGATAGCAGCAATAGCAGTGATAGTAGTGACAGCAGTGACAGTGACAGCAGTGATAGCAACAGTAGCAGTGATAGTGACAGCAGTGACAGTGACAGCAGTGATAGCAG
HAP15A    GACAGTAATGGCAATGGTAACAATGGGAATGATGACAATGACAAATCAGACAGTGGCAAAGGTAAATCAGATAGCAGTGACAGTGATAGTAGTGATAGCAGCAATAGCAGTGATAGTAGTGACAGCAGTGACAGTGACAGCAGTGATAGCAACAGTAGCAGTGATAGTGACAGCAGTGACAGTGACAGCAGTGATAGCAG
HAP17B    GACAGTAATGGCAATGGTAACAATGGGAATGATGACAATGACAAATCAGACAGTGGCAAAGGTAAATCAGATAGCAGTGACAGTGATAGTAGTGATAGCAGCAATAGCAGTGATAGTAGTGACAGCAGTGACAGTGACAGCAGTGATAGCAACAGTAGCAGTGATAGTGACAGCAGTGACAGTGACAGCAGTGATAGCAG
HAP20A    GACAGTAATGGCAATGGTAACAATGGGAATGATGACAATGACAAATCAGACAGTGGCAAAGGTAAATCAGATAGCAGTGACAGTGATAGTAGTGATAGCAGCAATAGCAGTGATAGTAGTGACAGCAGTGACAGTGACAGCAGTGATAGCAACAGTAGCAGTGATAGTGACAGCAGTGACAGTGACAGCAGTGATAGCAG
HAP20B    GACAGTAATGGCAATGGTAACAATGGGAATGATGACAATGACAAATCAGACAGTGGCAAAGGTAAATCAGATAGCAGTGACAGTGATAGTAGTGATAGCAGCAATAGCAGTGATAGTAGTGACAGCAGTGACAGTGACAGCAGTGATAGCAACAGTAGCAGTGATAGTGACAGCAGTGACAGTGACAGCAGTGATAGCAG
HAP36A    GACAGTAATGGCAATGGTAACAATGGGAATGATGACAATGACAAATCAGACAGTGGCAAAGGTAAATCAGATAGCAGTGACAGTGATAGTAGTGATAGCAGCAATAGCAGTGATAGTAGTGACAGCAGTGACAGTGACAGCAGTGATAGCAACAGTAGCAGTGATAGTGACAGCAGTGACAGTGACAGCAGTGATAGCAG
HAP37A    GACAGTAATGGCAATGGTAACAATGGGAATGATGACAATGACAAATCAGACAGTGGCAAAGGTAAATCAGATAGCAGTGACAGTGATAGTAGTGATAGCAGCAATAGCAGTGATAGTAGTGACAGCAGTGACAGTGACAGCAGTGATAGCAACAGTAGCAGTGATAGTGACAGCAGTGACAGTGACAGCAGTGATAGCAG
HAP38A    GACAGTAATGGCAATGGTAACAATGGGAATGATGACAATGACAAATCAGACAGTGGCAAAGGTAAATCAGATAGCAGTGACAGTGATAGTAGTGATAGCAGCAATAGCAGTGATAGTAGTGACAGCAGTGACAGTGACAGCAGTGATAGCAACAGTAGCAGTGATAGTGACAGCAGTGACAGTGACAGCAGTGATAGCAG
SHAP1:    GACAGTAATGGCAATGGTAACAATGGGAATGATGACAATGACAAATCAGACAGTGGCAAAGGTAAATCAGATAGCAGTGACAGTGATAGTAGTGATAGCAGCAATAGCAGTGATAGTAGTGACAGCAGTGACAGTGACAGCAGTGATAGCAACAGTAGCAGTGATAGTGACAGCAGTGACAGTGACAGCAGTGATAGCAG
SHAP2:    GACAGTAATGGCAATGGTAACAATGGGAATGATGACAATGACAAATCAGACAGTGGCAAAGGTAAATCAGATAGCAGTGACAGTGATAGTAGTGATAGCAGCAATAGCAGTGATAGTAGTGACAGCAGTGACAGTGACAGCAGTGATAGCAACAGTAGCAGTGATAGTGACAGCAGTGACAGTGACAGCAGTGATAGCAG
SHAP3:    GACAGTAATGGCAATGGTAACAATGGGAATGATGACAATGACAAATCAGACAGTGGCAAAGGTAAATCAGATAGCAGTGACAGTGATAGTAGTGATAGCAGCAATAGCAGTGATAGTAGTGACAGCAGTGACAGTGACAGCAGTGATAGCAACAGTAGCAGTGATAGTGACAGCAGTGACAGTGACAGCAGTGATAGCAG
SHAP4:    GACAGTAATGGCAATGGTAACAATGGGAATGATGACAATGACAAATCAGACAGTGGCAAAGGTAAATCAGATAGCAGTGACAGTGATAGTAGTGATAGCAGCAATAGCAGTGATAGTAGTGACAGCAGTGACAGTGACAGCAGTGATAGCAACAGTAGCAGTGATAGTGACAGCAGTGACAGTGACAGCAGTGATAGCAG
SHAP5:    GACAGTAATGGCAATGGTAACAATGGGAATGATGACAATGACAAATCAGACAGTGGCAAAGGTAAATCAGATAGCAGTGACAGTGATAGTAGTGATAGCAGCAATAGCAGTGATAGTAGTGACAGCAGTGACAGTGACAGCAGTGATAGCAACAGTAGCAGTGATAGTGACAGCAGTGACAGTGACAGCAGTGATAGCAG
SHAP6:    GACAGTAATGGCAATGGTAACAATGGGAATGATGACAATGACAAATCAGACAGTGGCAAAGGTAAATCAGATAGCAGTGACAGTGATAGTAGTGATAGCAGCAATAGCAGTGATAGTAGTGACAGCAGTGACAGTGACAGCAGTGATAGCAACAGTAGCAGTGATAGTGACAGCAGTGACAGTGACAGCAGTGATAGCAG
SHAP6(2)  GACAGTAATGGCAATGGTAACAATGGGAATGATGACAATGACAAATCAGACAGTGGCAAAGGTAAATCAGATAGCAGTGACAGTGATAGTAGTGATAGCAGCAATAGCAGTGATAGTAGTGACAGCAGTGACAGTGACAGCAGTGATAGCAACAGTAGCAGTGATAGTGACAGCAGTGACAGTGACAGCAGTGATAGCAG
SHAP7     GACAGTAATGGCAATGGTAACAATGGGAATGATGACAATGACAAATCAGACAGTGGCAAAGGTAAATCAGATAGCAGTGACAGTGATAGTAGTGATAGCAGCAATAGCAGTGATAGTAGTGACAGCAGTGACAGTGACAGCAGTGATAGCAACAGTAGCAGTGATAGTGACAGCAGTGACAGTGACAGCAGTGATAGCAG
SHAP102   GACAGTAATGGCAATGGTAACAATGGGAATGATGACAATGACAAATCAGACAGTGGCAAAGGTAAATCAGATAGCAGTGACAGTGATAGTAGTGATAGCAGCAATAGCAGTGATAGTAGTGACAGCAGTGACAGTGACAGCAGTGATAGCAACAGTAGCAGTGATAGTGACAGCAGTGACAGTGACAGCAGTGATAGCAG
SHAP130   GACAGTAATGGCAATGGTAACAATGGGAATGATGACAATGACAAATCAGACAGTGGCAAAGGTAAATCAGATAGCAGTGACAGTGATAGTAGTGATAGCAGCAATAGCAGTGATAGTAGTGACAGCAGTGACAGTGACAGCAGTGATAGCAACAGTAGCAGTGATAGTGACAGCAGTGACAGTGACAGCAGTGATAGCAG
SHAP106   GACAGTAATGGCAATGGTAACAATGGGAATGATGACAATGACAAATCAGACAGTGGCAAAGGTAAATCAGATAGCAGTGACAGTGATAGTAGTGATAGCAGCAATAGCAGTGATAGTAGTGACAGCAGTGACAGTGACAGCAGTGATAGCAACAGTAGCAGTGATAGTGACAGCAGTGACAGTGACAGCAGTGATAGCAG
SHAP72    GACAGTAATGGCAATGGTAACAATGGGAATGATGACAATGACAAATCAGACAGTGGCAAAGGTAAATCAGATAGCAGTGACAGTGATAGTAGTGATAGCAGCAATAGCAGTGATAGTAGTGACAGCAGTGACAGTGACAGCAGTGATAGCAACAGTAGCAGTGATAGTGACAGCAGTGACAGTGACAGCAGTGATAGCAG
SHAP110   GACAGTAATGGCAATGGTAACAATGGGAATGATGACAATGACAAATCAGACAGTGGCAAAGGTAAATCAGATAGCAGTGACAGTGATAGTAGTGATAGCAGCAATAGCAGTGATAGTAGTGACAGCAGTGACAGTGACAGCAGTGATAGCAACAGTAGCAGTGATAGTGACAGCAGTGACAGTGACAGCAGTGATAGCAG
MERGED    GACAGTAATGGCAATGGTAACAATGGGAATGATGACAATGACAAATCAGACAGTGGCAAAGGTAAATCAGATAGCAGTGACAGTGATAGTAGTGATAGCAGCAATAGCAGTGATAGTAGTGACAGCAGTGACAGTGACAGCAGTGATAGCAACAGTAGCAGTGATAGTGACAGCAGTGACAGTGACAGCAGTGATAGCAG
TRANSL     D  S  N  G  N  G  N  N  G  N  D  D  N  D  K  S  D  S  G  K  G  K  S  D  S  S  D  S  D  S  S  D  S  S  N  S  S  D  S  S  D  S  S  D  S  D  S  S  D  S  N  S  S  S  D  S  D  S  S  D  S  D  S  S  D  S  S   591
```

Mutations:

1. g.10820delT; c.1686delT; p.(Asp562Glufs*752) [5]

	1773 … 1830 … 1870_1873 1874-1877 … 1915_1918/1918_1921/1922_1925 … 1972	
REFSEQ	TGACAGTGATAGTAGTGATAGCAGCAATAGCAGTGACAGTAGTGACAGCAGTGATAG**C**AGTGACAGTAGTGATAGTAGTGACAGCAGTGACAGCAAG**TCAGACAG**CAGCAAATCAGAGAGCGACAGCAGTGATAGTGACAGT**AAGTCAGACAG**CAGTGACAGCAACAGCAGTGACAGTAGTGACAACAGTGATAGCAGCG	
Fam8a	TGACAGTGATAGTAGTGATAGCAGCAATAGCAGTGACAGTAGTGACAGCAGTGATAGCAGTGACAGTAGTGATAGTAGTGACAGCAGTGACAGCAAGTCAGACAGCAGCAAATCAGAGAGCGACAGCAGTGATAGTGACAGTAAGTCAGACAGCAGTGACAGCAACAGCAGTGACAGTAGTGACAACAGTGATAGCAG**T**G	
Fam8b	TGACAGTGATAGTAGTGATAGCAGCAATAGCAGTGACAGTAGTGACAGCAGTGATAGCAGTGACAGTAGTGATAGTAGTGACAGCAGTGACAGCAAGTCAGACAGCAGCAAATCAGAGAGCGACAGCAGTGATAGTGACAGTAAGTCAGACAGCAGTGACAGCAACAGCAGTGACAGTAGTGACAACAGTGATAGCAGCG	
Fam9a	TGACAGTGATAGTAGTGATAGCAGCAATAGCAGTGACAGTAGTGACAGCAGTGATAGCAGTGACAGTAGTGATAGTAGTGACAGCAGTGACAGCAAGTCAGACAGCAGCAAATCAGAGAGCGACAGCAGTGATAGTGACAGTAAGTCAGACAGCAGTGACAGCAACAGCAGTGACAGTAGTGACAACAGTGATAGCAGCG	
Fam9b	TGACAGTGATAGTAGTGATAGCAGCAATAGCAGTGACAGTAGTGACAGCAGTGATAGCAGTGACAGTAGTGATAGTAGTGACAGCAGTGACAGCAAGTCAGACAGCAGCAAATCAGAGAGCGACAGCAGTGATAGTGACAGTAAGTCAGACAGCAGTGACAGCAACAGCAGTGACAGTAGTGACAACAGTGATAGCAGCG	
Fam10a	TGACAGTGATAGTAGTGATAGCAGCAATAGCAGTGACAGTAGTGACAGCAGTGATAGCAGTGACAGTAGTGATAGTAGTGACAGCAGTGACAGCAAGTCAGACAGCAGCAAATCAGAGAGCGACAGCAGTGATAGTGACAGTAAGTCAGACAGCAGTGACAGCAACAGCAGTGACAGTAGTGACAACAGTGATAGCAGCG	
Fam10b	TGACAGTGATAGTAGTGATAGCAGCAATAGCAGTGACAGTAGTGACAGCAGTGATAGCAGTGACAGTAGTGATAGTAGTGACAGCAGTGACAGCAAGTCAGACAGCAGCAAATCAGAGAGCGACAGCAGTGATAGTGACAGTAAGTCAGACAGCAGTGACAGCAACAGCAGTGACAGTAGTGACAACAGTGATAGCAG**T**G	
Fam11a	TGACAGTGATAGTAGTGATAGCAGCAATAGCAGTGACAGTAGTGACAGCAGTGATAGCAGTGACAGTAGTGATAGTAGTGACAGCAGTGACAGCAAGTCAGACAGCAGCAAATCAGAGAGCGACAGCAGTGATAGTGACAGTAAGTCAGACAGCAGTGACAGCAACAGCAGTGACAGTAGTGACAACAGTGATAGCAG**T**G	
Fam11b	TGACAGTGATAGTAGTGATAGCAGCAATAGCAGTGACAGTAGTGACAGCAGTGATAGCAGTGACAGTAGTGATAGTAGTGACAGCAGTGACAGCAAGTCAGACAGCAGCAAATCAGAGAGCGACAGCAGTGATAGTGACAGTAAGTCAGACAGCAGTGACAGCAACAGCAGTGACAGTAGTGACAACAGTGATAGCAG**T**G	
Fam12a	TGACAGTGATAGTAGTGATAGCAGCAATAGCAGTGACAGTAGTGACAGCAGTGATAGCAGTGACAGTAGTGATAGTAGTGACAGCAGTGACAGCAAGTCAGACAGCAGCAAATCAGAGAGCGACAGCAGTGATAGTGACAGTAAGTCAGACAGCAGTGACAGCAACAGCAGTGACAGTAGTGACAACAGTGATAGCAGCG	
Fam12b	TGACAGTGATAGTAGTGATAGCAGCAATAGCAGTGACAGTAGTGACAGCAGTGATAGCAGTGACAGTAGTGATAGTAGTGACAGCAGTGACAGCAAGTCAGACAGCAGCAAATCAGAGAGCGACAGCAGTGATAGTGACAGTAAGTCAGACAGCAGTGACAGCAACAGCAGTGACAGTAGTGACAACAGTGATAGCAG**T**G	
F1A	TGACAGTGATAGTAGTGATAGCAGCAATAGCAGTGACAGTAGTGACAGCAGTGATAGCAGTGACAGTAGTGATAGTAGTGACAGCAGTGACAGCAAGTCAGACAGCAGCAAATCAGAGAGCGACAGCAGTGATAGTGACAGTAAGTCAGACAGCAGTGACAGCAACAGCAGTGACAGTAGTGACAACAGTGATAGCAGCG	
F1B	TGACAGTGATAGTAGTGATAGCAGCAATAGCAGTGACAGTAGTGACAGCAGTGATAGCAGTGACAGTAGTGATAGTAGTGACAGCAGTGACAGCAAGTCAGACAGCAGCAAATCAGAGAGCGACAGCAGTGATAGTGACAGTAAGTCAGACAGCAGTGACAGCAACAGCAGTGACAGTAGTGACAACAGTGATAGCAGCG	
F2A	TGACAGTGATAGTAGTGATAGCAGCAATAGCAGTGACAGTAGTGACAGCAGTGATAGCAGTGACAGTAGTGATAGTAGTGACAGCAGTGACAGCAAGTCAGACAGCAGCAAATCAGAGAGCGACAGCAGTGATAGTGACAGTAAGTCAGACAGCAGTGACAGCAACAGCAGTGACAGTAGTGACAACAGTGATAGCAG**T**G	
F2B	TGACAGTGATAGTAGTGATAGCAGCAATAGCAGTGACAGTAGTGACAGCAGTGATAGCAGTGACAGTAGTGATAGTAGTGACAGCAGTGACAGCAAGTCAGACAGCAGCAAATCAGAGAGCGACAGCAGTGATAGTGACAGTAAGTCAGACAGCAGTGACAGCAACAGCAGTGACAGTAGTGACAACAGTGATAGCAGCG	
F3A	TGACAGTGATAGTAGTGATAGCAGCAATAGCAGTGACAGTAGTGACAGCAGTGATAGCAGTGACAGTAGTGATAGTAGTGACAGCAGTGACAGCAAGTCAGACAGCAGCAAATCAGAGAGCGACAGCAGTGATAGTGACAGTAAGTCAGACAGCAGTGACAGCAACAGCAGTGACAGTAGTGACAACAGTGATAGCAG**T**G	
F3B	TGACAGTGATAGTAGTGATAGCAGCAATAGCAGTGACAGTAGTGACAGCAGTGATAGCAGTGACAGTAGTGATAGTAGTGACAGCAGTGACAGCAAGTCAGACAGCAGCAAATCAGAGAGCGACAGCAGTGATAGTGACAGTAAGTCAGACAGCAGTGACAGCAACAGCAGTGACAGTAGTGACAACAGTGATAGCAGCG	
F4A	TGACAGTGATAGTAGTGATAGCAGCAATAGCAGTGACAGTAGTGACAGCAGTGATAGCAGTGACAGTAGTGATAGTAGTGACAGCAGTGACAGCAAGTCAGACAGCAGCAAATCAGAGAGCGACAGCAGTGATAGTGACAGTAAGTCAGACAGCAGTGACAGCAACAGCAGTGACAGTAGTGACAACAGTGATAGCAGCG	
F4B	TGACAGTGATAGTAGTGATAGCAGCAATAGCAGTGACAGTAGTGACAGCAGTGATAGCAGTGACAGTAGTGATAGTAGTGACAGCAGTGACAGCAAGTCAGACAGCAGCAAATCAGAGAGCGACAGCAGTGATAGTGACAGTAAGTCAGACAGCAGTGACAGCAACAGCAGTGACAGTAGTGACAACAGTGATAGCAGCG	
F5A	TGACAGTGATAGTAGTGATAGCAGCAATAGCAGTGACAGTAGTGACAGCAGTGATAGCAGTGACAGTAGTGATAGTAGTGACAGCAGTGACAGCAAGTCAGACAGCAGCAAATCAGAGAGCGACAGCAGTGATAGTGACAGTAAGTCAGACAGCAGTGACAGCAACAGCAGTGACAGTAGTGACAACAGTGATAGCAGCG	
F5B	TGACAGTGATAGTAGTGATAGCAGCAATAGCAGTGACAGTAGTGACAGCAGTGATAGCAGTGACAGTAGTGATAGTAGTGACAGCAGTGACAGCAAGTCAGACAGCAGCAAATCAGAGAGCGACAGCAGTGATAGTGACAGTAAGTCAGACAGCAGTGACAGCAACAGCAGTGACAGTAGTGACAACAGTGATAGCAG**T**G	
HAP1A	TGACAGTGATAGTAGTGATAGCAGCAATAGCAGTGACAGTAGTGACAGCAGTGATAGCAGTGACAGTAGTGATAGTAGTGACAGCAGTGACAGCAAGTCAGACAGCAGCAAATCAGAGAGCGACAGCAGTGATAGTGACAGTAAGTCAGACAGCAGTGACAGCAACAGCAGTGACAGTAGTGACAACAGTGATAGCAGCG	
HAP2A	TGACAGTGATAGTAGTGATAGCAGCAATAGCAGTGACAGTAGTGACAGCAGTGATAGCAGTGACAGTAGTGATAGTAGTGACAGCAGTGACAGCAAGTCAGACAGCAGCAAATCAGAGAGCGACAGCAGTGATAGTGACAGTAAGTCAGACAGCAGTGACAGCAACAGCAGTGACAGTAGTGACAACAGTGATAGCAGCG	
HAP3A	TGACAGTGATAGTAGTGATAGCAGCAATAGCAGTGACAGTAGTGACAGCAGTGATAGCAGTGACAGTAGTGATAGTAGTGACAGCAGTGACAGCAAGTCAGACAGCAGCAAATCAGAGAGCGACAGCAGTGATAGTGACAGTAAGTCAGACAGCAGTGACAGCAACAGCAGTGACAGTAGTGACAACAGTGATAGCAGCG	
HAP15A	TGACAGTGATAGTAGTGATAGCAGCAATAGCAGTGACAGTAGTGACAGCAGTGATAGCAGTGACAGTAGTGATAGTAGTGACAGCAGTGACAGCAAGTCAGACAGCAGCAAATCAGAGAGCGACAGCAGTGATAGTGACAGTAAGTCAGACAGCAGTGACAGCAACAGCAGTGACAGTAGTGACAACAGTGATAGCAG**T**G	
HAP17B	TGACAGTGATAGTAGTGATAGCAGCAATAGCAGTGACAGTAGTGACAGCAGTGATAGCAGTGACAGTAGTGATAGTAGTGACAGCAGTGACAGCAAGTCAGACAGCAGCAAATCAGAGAGCGACAGCAGTGATAGTGACAGTAAGTCAGACAGCAGTGACAGCAACAGCAGTGACAGTAGTGACAACAGTGATAGCAGCG	
HAP20A	TGACAGTGATAGTAGTGATAGCAGCAATAGCAGTGACAGTAGTGACAGCAGTGATAGCAGTGACAGTAGTGATAGTAGTGACAGCAGTGACAGCAAGTCAGACAGCAGCAAATCAGAGAGCGACAGCAGTGATAGTGACAGTAAGTCAGACAGCAGTGACAGCAACAGCAGTGACAGTAGTGACAACAGTGATAGCAGCG	
HAP20B	TGACAGTGATAGTAGTGATAGCAGCAATAGCAGTGACAGTAGTGACAGCAGTGATAGCAGTGACAGTAGTGATAGTAGTGACAGCAGTGACAGCAAGTCAGACAGCAGCAAATCAGAGAGCGACAGCAGTGATAGTGACAGTAAGTCAGACAGCAGTGACAGCAACAGCAGTGACAGTAGTGACAACAGTGATAGCAGCG	
HAP36A	TGACAGTGATAGTAGTGATAGCAGCAATAGCAGTGACAGTAGTGACAGCAGTGATAGCAGTGACAGTAGTGATAGTAGTGACAGCAGTGACAGCAAGTCAGACAGCAGCAAATCAGAGAGCGACAGCAGTGATAGTGACAGTAAGTCAGACAGCAGTGACAGCAACAGCAGTGACAGTAGTGACAACAGTGATAGCAGCG	
HAP37A	TGACAGTGATAGTAGTGATAGCAGCAATAGCAGTGACAGTAGTGACAGCAGTGATAGCAGTGACAGTAGTGATAGTAGTGACAGCAGTGACAGCAAGTCAGACAGCAGCAAATCAGAGAGCGACAGCAGTGATAGTGACAGTAAGTCAGACAGCAGTGACAGCAACAGCAGTGACAGTAGTGACAACAGTGATAGCAGCG	
HAP38A	TGACAGTGATAGTAGTGATAGCAGCAATAGCAGTGACAGTAGTGACAGCAGTGATAGCAGTGACAGTAGTGATAGTAGTGACAGCAGTGACAGCAAGTCAGACAGCAGCAAATCAGAGAGCGACAGCAGTGATAGTGACAGTAAGTCAGACAGCAGTGACAGCAACAGCAGTGACAGTAGTGACAACAGTGATAGCAGCG	
SHAP1:	TGACAGTGATAGTAGTGATAGCAGCAATAGCAGTGACAGTAGTGACAGCAGTGATAGCAGTGACAGTAGTGATAGTAGTGACAGCAGTGACAGCAAGTCAGACAGCAGCAAATCAGAGAGCGACAGCAGTGATAGTGACAGTAAGTCAGACAGCAGTGACAGCAACAGCAGTGACAGTAGTGACAACAGTGATAGCAGCG	
SHAP2:	TGACAGTGATAGTAGTGATAGCAGCAATAGCAGTGACAGTAGTGACAGCAGTGATAGCAGTGACAGTAGTGATAGTAGTGACAGCAGTGACAGCAAGTCAGACAGCAGCAAATCAGAGAGCGACAGCAGTGATAGTGACAGTAAGTCAGACAGCAGTGACAGCAACAGCAGTGACAGTAGTGACAACAGTGATAGCAGCG	
SHAP3:	TGACAGTGATAGTAGTGATAGCAGCAATAGCAGTGACAGTAGTGACAGCAGTGATAGCAGTGACAGTAGTGATAGTAGTGACAGCAGTGACAGCAAGTCAGACAGCAGCAAATCAGAGAGCGACAGCAGTGATAGTGACAGTAAGTCAGACAGCAGTGACAGCAACAGCAGTGACAGTAGTGACAACAGTGATAGCAG**T**G	
SHAP4:	TGACAGTGATAGTAGTGATAGCAGCAATAGCAGTGACAGTAGTGACAGCAGTGATAGCAGTGACAGTAGTGATAGTAGTGACAGCAGTGACAGCAAGTCAGACAGCAGCAAATCAGAGAGCGACAGCAGTGATAGTGACAGTAAGTCAGACAGCAGTGACAGCAACAGCAGTGACAGTAGTGACAACAGTGATAGCAGCG	
SHAP5:	TGACAGTGATAGTAGTGATAGCAGCAATAGCAGTGACAGTAGTGACAGCAGTGATAGCAGTGACAGTAGTGATAGTAGTGACAGCAGTGACAGCAAGTCAGACAGCAGCAAATCAGAGAGCGACAGCAGTGATAGTGACAGTAAGTCAGACAGCAGTGACAGCAACAGCAGTGACAGTAGTGACAACAGTGATAGCAGCG	
SHAP6:	TGACAGTGATAGTAGTGATAGCAGCAATAGCAGTGACAGTAGTGACAGCAGTGATAGCAGTGACAGTAGTGATAGTAGTGACAGCAGTGACAGCAAGTCAGACAGCAGCAAATCAGAGAGCGACAGCAGTGATAGTGACAGTAAGTCAGACAGCAGTGACAGCAACAGCAGTGACAGTAGTGACAACAGTGATAGCAGCG	
SHAP6(2)	TGACAGTGATAGTAGTGATAGCAGCAATAGCAGTGACAGTAGTGACAGCAGTGATAGCAGTGACAGTAGTGATAGTAGTGACAGCAGTGACAGCAAGTCAGACAGCAGCAAATCAGAGAGCGACAGCAGTGATAGTGACAGTAAGTCAGACAGCAGTGACAGCAACAGCAGTGACAGTAGTGACAACAGTGATAGCAGCG	
SHAP7	TGACAGTGATAGTAGTGATAGCAGCAATAGCAGTGACAGTAGTGACAGCAGTGATAGCAGTGACAGTAGTGATAGTAGTGACAGCAGTGACAGCAAGTCAGACAGCAGCAAATCAGAGAGCGACAGCAGTGATAGTGACAGTAAGTCAGACAGCAGTGACAGCAACAGCAGTGACAGTAGTGACAACAGTGATAGCAGCG	
SHAP102	TGACAGTGATAGTAGTGATAGCAGCAATAGCAGTGACAGTAGTGACAGCAGTGATAGCAGTGACAGTAGTGATAGTAGTGACAGCAGTGACAGCAAGTCAGACAGCAGCAAATCAGAGAGCGACAGCAGTGATAGTGACAGTAAGTCAGACAGCAGTGACAGCAACAGCAGTGACAGTAGTGACAACAGTGATAGCAGCG	
SHAP130	TGACAGTGATAGTAGTGATAGCAGCAATAGCAGTGACAGTAGTGACAGCAGTGATAGCAGTGACAGTAGTGATAGTAGTGACAGCAGTGACAGCAAGTCAGACAGCAGCAAATCAGAGAGCGACAGCAGTGATAGTGACAGTAAGTCAGACAGCAGTGACAGCAACAGCAGTGACAGTAGTGACAACAGTGATAGCAGCG	
SHAP106	TGACAGTGATAGTAGTGATAGCAGCAATAGCAGTGACAGTAGTGACAGCAGTGATAGCAGTGACAGTAGTGATAGTAGTGACAGCAGTGACAGCAAGTCAGACAGCAGCAAATCAGAGAGCGACAGCAGTGATAGTGACAGTAAGTCAGACAGCAGTGACAGCAACAGCAGTGACAGTAGTGACAACAGTGATAGCAGCG	
SHAP72	TGACAGTGATAGTAGTGATAGCAGCAATAGCAGTGACAGTAGTGACAGCAGTGATAGCAGTGACAGTAGTGATAGTAGTGACAGCAGTGACAGCAAGTCAGACAGCAGCAAATCAGAGAGCGACAGCAGTGATAGTGACAGTAAGTCAGACAGCAGTGACAGCAACAGCAGTGACAGTAGTGACAACAGTGATAGCAGCG	
SHAP110	TGACAGTGATAGTAGTGATAGCAGCAATAGCAGTGACAGTAGTGACAGCAGTGATAGCAGTGACAGTAGTGATAGTAGTGACAGCAGTGACAGCAAGTCAGACAGCAGCAAATCAGAGAGCGACAGCAGTGATAGTGACAGTAAGTCAGACAGCAGTGACAGCAACAGCAGTGACAGTAGTGACAACAGTGATAGCAGCG	
MERGED	TGACAGTGATAGTAGTGATAGCAGCAATAGCAGTGACAGTAGTGACAGCAGTGATAGCAGTGACAGTAGTGATAGTAGTGACAGCAGTGACAGCAAGTCAGACAGCAGCAAATCAGAGAGCGACAGCAGTGATAGTGACAGTAAGTCAGACAGCAGTGACAGCAACAGCAGTGACAGTAGTGACAACAGTGATAGCAGCG	
TRANSL	D S D S S D S S N S S D S S D S S D S S D S S D S S D S K S D S S K S E S D S S D S D S K S D S S D S N S S D S S D N S D S S	657

Mutations:

2. g.10964delC; c.1830del**C**; p.(Ser610Argfs*704) [5]
3. g.11004_11007delTCAG; c.1870_1873del**TCAG**; p.(Ser624Thrfs*689) [2]
4. g.11008_11011delACAG; c.1874_1877del**ACAG**; p.(Asp625Alafs*688) [6]
5. g.11049_11052delAAGT; c.1915_1918del**AAGT**; p.(Lys639Glnfs*674) [1,7]
6. g.11052_11055delTCAG; c.1918_1921del**TCAG**; p.(Ser640Thrfs*673) [1,2,5]
7. g.11056_11059delACAG; c.1922_1925del**ACAG**; p.(Asp641Alafs*672) [5]

```
         1973                                                                   ID1  2040                        ID2                      2063                                                                                                   2134               2154
REFSEQ   ACAGCAGCAATAGCAGTAACAGCAGTGATAGTAGTGACAGCAGTGATAGCAGTGACAGCAGCAGTAGCAGTGACAGCAGCA------------------ACAGCAGTGATAGTAGTGACAGTAGTGACAGCAGCAATAGCAGTGAGAGCAGTGATAGTAGTGACAGCAGTGATAGTGACAGCAGTGATAGTAGTGACAGC
Fam8a    ACAGCAGCAATAGCAGTAACAGCAGTGATAGTAGTGACAGCAGTGATAGCAGTGACAGCAGCAGTAGCAGTGACAGCAGCA------------------ACAGCAGTGATAGTAGTGACAGTAGTGACAGCAGCAATAGCAGTGAGAGCAGTGATAGTAGTGACAGCAGTGATAGTGACAGCAGTGATAGTAGTGACAGC
Fam8b    ACAGCAGCAATAGCAGTAACAGCAGTGATAGTAGTGACAGCAGTGATAGCAGTGACAGCAGCAGTAGCAGTGACAGCAGCA------------------ACAGCAGTGATAGTAGTGACAGTAGTGACAGCAGCAATAGCAGTGAGAGCAGTGATAGTAGTGACAGCAGTGATAGTGACAGCAGTGATAGTAGTGACAGC
Fam9a    ACAGCAGCAATAGCAGTAACAGCAGTGATAGTAGTGACAGCAGTGATAGCAGTGACAGCAGCAGTAGCAGTGACAGCAGCA------------------ACAGCAGTGATAGTAGTGACAGTAGTGACAGCAGCAATAGCAGTGAGAGCAGTGATAGTAGTGACAGCAGTGATAGTGACAGCAGTGATAGTAGTGACAGC
Fam9b    ACAGCAGCAATAGCAGTAACAGCAGTGATAGTAGTGACAGCAGTGATAGCAGTGACAGCAGCAGTAGCAGTGACAGCAGCAGTAGCAGTGACAGCAGCAACAGCAGTGATAGTAGTGACAGTAGTGACAGCAGCAATAGCAGTGAGAGCAGTGATAGTAGTGACAGCAGTGATAGTGACAGCAGTGATAGTAGTGACAGC
Fam10a   ACAGCAGCAATAGCAGTAACAGCAGTGATAGTAGTGACAGCAGTGATAGCAGTGACAGCAGCAGTAGCAGTGACAGCAGCAGTAGCAGTGACAGCAGCAACAGCAGTGATAGTAGTGACAGTAGTGACAGCAGCAATAGCAGTGAGAGCAGTGATAGTAGTGACAGCAGTGATAGTGACAGCAGTGATAGTAGTGACAGC
Fam10b   ACAGCAGCAATAGCAGTAACAGCAGTGATAGTAGTGACAGCAGTGATAGCAGTGACAGCAGCAGTAGCAGTGACAGCAGCA------------------ACAGCAGTGATAGTAGTGACAGTAGTGACAGCAGCAATAGCAGTGAGAGCAGTGATAGTAGTGACAGCAGTGATAGTGACAGCAGTGATAGTAGTGACAGC
Fam11a   ACAGCAGCAATAGCAGTAACAGCAGTGATAGTAGTGACAGCAGTGATAGCAGTGACAGCAGCAGTAGCAGTGACAGCAGCA------------------ACAGCAGTGATAGTAGTGACAGTAGTGACAGCAGCAATAGCAGTGAGAGCAGTGATAGTAGTGACAGCAGTGATAGTGACAGCAGTGATAGTAGTGACAGC
Fam11b   ACAGCAGCAATAGCAGTAACAGCAGTGATAGTAGTGACAGCAGTGATAGCAGTGACAGCAGCAGTAGCAGTGACAGCAGCA------------------ACAGCAGTGATAGTAGTGACAGTAGTGACAGCAGCAATAGCAGTGAGAGCAGTGATAGTAGTGACAGCAGTGATAGTGACAGCAGTGATAGTAGTGACAGC
Fam12a   ACAGCAGCAATAGCAGTAACAGCAGTGATAGTAGTGACAGCAGTGATAGCAGTGACAGCAGCAGTAGCAGTGACAGCAGCAGTAGCAGTGACAGCAGCAACAGCAGTGATAGTAGTGACAGTAGTGACAGCAGCAATAGCAGTGAGAGCAGTGATAGTAGTGACAGCAGTGATAGTGACAGCAGTGATAGTAGTGACAGC
Fam12b   ACAGCAGCAATAGCAGTAACAGCAGTGATAGTAGTGACAGCAGTGATAGCAGTGACAGCAGCAGTAGCAGTGACAGCAGCA------------------ACAGCAGTGATAGTAGTGACAGTAGTGACAGCAGCAATAGCAGTGAGAGCAGTGATAGTAGTGACAGCAGTGATAGTGACAGCAGTGATAGTAGTGACAGC
F1A      ACAGCAGCAATAGCAGTAACAGCAGTGATAGTAGTGACAGCAGTGATAGCAGTGACAGCAGCAGTAGCAGTGACAGCAGCAGTAGCAGTGACAGCAGCAACAGCAGTGATAGTAGTGACAGTAGTGACAGCAGCAATAGCAGTGAGAGCAGTGATAGTAGTGACAGCAGTGATAGTGACAGCAGTGATAGTAGTGACAGC
F1B      ACAGCAGCAATAGCAGTAACAGCAGTGATAGTAGTGACAGCAGTGATAGCAGTGACAGCAGCAGTAGCAGTGACAGCAGCAGTAGCAGTGACAGCAGCAACAGCAGTGATAGTAGTGACAGTAGTGACAGCAGCAATAGCAGTGAGAGCAGTGATAGTAGTGACAGCAGTGATAGTGACAGCAGTGATAGTAGTGACAGC
F2A      ACAGCAGCAATAGCAGTAACAGCAGTGATAGTAGTGACAGCAGTGATAGCAGTGACAGCAGCAGTAGCAGTGACAGCAGCA------------------ACAGCAGTGATAGTAGTGACAGTAGTGACAGCAGCAATAGCAGTGAGAGCAGTGATAGTAGTGACAGCAGTGATAGTGACAGCAGTGATAGTAGTGACAGC
F2B      ACAGCAGCAATAGCAGTAACAGCAGTGATAGTAGTGACAGCAGTGATAGCAGTGACAGCAGCAGTAGCAGTGACAGCAGCA------------------ACAGCAGTGATAGTAGTGACAGTAGTGACAGCAGCAATAGCAGTGAGAGCAGTGATAGTAGTGACAGCAGTGATAGTGACAGCAGTGATAGTAGTGACAGC
F3A      ACAGCAGCAATAGCAGTAACAGCAGTGATAGTAGTGACAGCAGTGATAGCAGTGACAGCAGCAGTAGCAGTGACAGCAGCA------------------ACAGCAGTGATAGTAGTGACAGTAGTGACAGCAGCAATAGCAGTGAGAGCAGTGATAGTAGTGACAGCAGTGATAGTGACAGCAGTGATAGTAGTGACAGC
F3B      ACAGCAGCAATAGCAGTAACAGCAGTGATAGTAGTGACAGCAGTGATAGCAGTGACAGCAGCAGTAGCAGTGACAGCAGCA------------------ACAGCAGTGATAGTAGTGACAGTAGTGACAGCAGCAATAGCAGTGAGAGCAGTGATAGTAGTGACAGCAGTGATAGTGACAGCAGTGATAGTAGTGACAGC
F4A      ACAGCAGCAATAGCAGTAACAGCAGTGATAGTAGTGACAGCAGTGATAGCAGTGACAGCAGCAGTAGCAGTGACAGCAGCA------------------ACAGCAGTGATAGTAGTGACAGTAGTGACAGCAGCAATAGCAGTGAGAGCAGTGATAGTAGTGACAGCAGTGATAGTGACAGCAGTGATAGTAGTGACAGC
F4B      ACAGCAGCAATAGCAGTAACAGCAGTGATAGTAGTGACAGCAGTGATAGCAGTGACAGCAGCAGTAGCAGTGACAGCAGCA------------------ACAGCAGTGATAGTAGTGACAGTAGTGACAGCAGCAATAGCAGTGAGAGCAGTGATAGTAGTGACAGCAGTGATAGTGACAGCAGTGATAGTAGTGACAGC
F5A      ACAGCAGCAATAGCAGTAACAGCAGTGATAGTAGTGACAGCAGTGATAGCAGTGACAGCAGCAGTAGCAGTGACAGCAGCA------------------ACAGCAGTGATAGTAGTGACAGTAGTGACAGCAGCAATAGCAGTGAGAGCAGTGATAGTAGTGACAGCAGTGATAGTGACAGCAGTGATAGTAGTGACAGC
F5B      ACAGCAGCAATAGCAGTAACAGCAGTGATAGTAGTGACAGCAGTGATAGCAGTGACAGCAGCAGTAGCAGTGACAGCAGCA------------------ACAGCAGTGATAGTAGTGACAGTAGTGACAGCAGCAATAGCAGTGAGAGCAGTGATAGTAGTGACAGCAGTGATAGTGACAGCAGTGATAGTAGTGACAGC
HAP1A    ACAGCAGCAATAGCAGTAACAGCAGTGATAGTAGTGACAGCAGTGATAGCAGTGACAGCAGCAGTAGCAGTGACAGCAGCAGTAGCAGTGACAGCAGCAACAGCAGTGATAGTAGTGACAGTAGTGACAGCAGCAATAGCAGTGAGAGCAGTGATAGTAGTGACAGCAGTGATAGTGACAGCAGTGATAGTAGTGACAGC
HAP2A    ACAGCAGCAATAGCAGTAACAGCAGTGATAGTAGTGACAGCAGTGATAGCAGTGACAGCAGCAGTAGCAGTGACAGCAGCA------------------ACAGCAGTGATAGTAGTGACAGTAGTGACAGCAGCAATAGCAGTGAGAGCAGTGATAGTAGTGACAGCAGTGATAGTGACAGCAGTGATAGTAGTGACAGC
HAP3A    ACAGCAGCAATAGCAGTAACAGCAGTGATAGTAGTGACAGCAGTGATAGCAGTGACAGCAGCAGTAGCAGTGACAGCAGCA------------------ACAGCAGTGATAGTAGTGACAGTAGTGACAGCAGCAATAGCAGTGAGAGCAGTGATAGTAGTGACAGCAGTGATAGTGACAGCAGTGATAGTAGTGACAGC
HAP15A   ACAGCAGCAATAGCAGTAACAGCAGTGATAGTAGTGACAGCAGTGATAGCAGTGACAGCAGCAGTAGCAGTGACAGCAGCA------------------ACAGCAGTGATAGTAGTGACAGTAGTGACAGCAGCAATAGCAGTGAGAGCAGTGATAGTAGTGACAGCAGTGATAGTGACAGCAGTGATAGTAGTGACAGC
HAP17B   ACAGCAGCAATAGCAGTAACAGCAGTGATAGTAGTGACAGCAGTGATAGCAGTGACAGCAGCAGTAGCAGTGACAGCAGCAGTAGCAGTGACAGCAGCAACAGCAGTGATAGTAGTGACAGTAGTGACAGCAGCAATAGCAGTGAGAGCAGTGATAGTAGTGACAGCAGTGATAGTGACAGCAGTGATAGTAGTGACAGC
HAP20A   ACAGCAGCAATAGCAGTAACAGCAGTGATAGTAGTGACAGCAGTGATAGCAGTGACAGCAGCAGTAGCAGTGACAGCAGCA------------------ACAGCAGTGATAGTAGTGACAGTAGTGACAGCAGCAATAGCAGTGAGAGCAGTGATAGTAGTGACAGCAGTGATAGTGACAGCAGTGATAGTAGTGACAGC
HAP20B   ACAGCAGCAATAGCAGTAACAGCAGTGATAGTAGTGACAGCAGTGATAGCAGTGACAGCAGCAGTAGCAGTGACAGCAGCA------------------ACAGCAGTGATAGTAGTGACAGTAGTGACAGCAGCAATAGCAGTGAGAGCAGTGATAGTAGTGACAGCAGTGATAGTGACAGCAGTGATAGTAGTGACAGC
HAP36A   ACAGCAGCAATAGCAGTAACAGCAGTGATAGTAGTGACAGCAGTGATAGCAGTGACAGCAGC------AGTGACAGCAGCA------------------ACAGCAGTGATAGTAGTGACAGTAGTGACAGCAGCAATAGCAGTGAGAGCAGTGATAGTAGTGACAGCAGTGATAGTGACAGCAGTGATAGTAGTGACAGC
HAP37A   ACAGCAGCAATAGCAGTAACAGCAGTGATAGTAGTGACAGCAGTGATAGCAGTGACAGCAGCAGTAGCAGTGACAGCAGCA------------------ACAGCAGTGATAGTAGTGACAGTAGTGACAGCAGCAATAGCAGTGAGAGCAGTGATAGTAGTGACAGCAGTGATAGTGACAGCAGTGATAGTAGTGACAGC
HAP38A   ACAGCAGCAATAGCAGTAACAGCAGTGATAGTAGTGACAGCAGTGATAGCAGTGACAGCAGCAGTAGCAGTGACAGCAGCA------------------ACAGCAGTGATAGTAGTGACAGTAGTGACAGCAGCAATAGCAGTGAGAGCAGTGATAGTAGTGACAGCAGTGATAGTGACAGCAGTGATAGTAGTGACAGC
SHAP1:   ACAGCAGCAATAGCAGTAACAGCAGTGATAGTAGTGACAGCAGTGATAGCAGTGACAGCAGCAGTAGCAGTGACAGCAGCAGTAGCAGTGACAGCAGCAACAGCAGTGATAGTAGTGACAGTAGTGACAGCAGCAATAGCAGTGAGAGCAGTGATAGTAGTGACAGCAGTGATAGTGACAGCAGTGATAGTAGTGACAGC
SHAP2:   ACAGCAGCAATAGCAGTAACAGCAGTGATAGTAGTGACAGCAGTGATAGCAGTGACAGCAGCAGTAGCAGTGACAGCAGCA------------------ACAGCAGTGATAGTAGTGACAGTAGTGACAGCAGCAATAGCAGTGAGAGCAGTGATAGTAGTGACAGCAGTGATAGTGACAGCAGTGATAGTAGTGACAGC
SHAP3:   ACAGCAGCAATAGCAGTAACAGCAGTGATAGTAGTGACAGCAGTGATAGCAGTGACAGCAGCAGTAGCAGTGACAGCAGCA------------------ACAGCAGTGATAGTAGTGACAGTAGTGACAGCAGCAATAGCAGTGAGAGCAGTGATAGTAGTGACAGCAGTGATAGTGACAGCAGTGATAGTAGTGACAGC
SHAP4:   ACAGCAGCAATAGCAGTAACAGCAGTGATAGTAGTGACAGCAGTGATAGCAGTGACAGCAGCAGTAGCAGTGACAGCAGCA------------------ACAGCAGTGATAGTAGTGACAGTAGTGACAGCAGCAATAGCAGTGAGAGCAGTGATAGTAGTGACAGCAGTGATAGTGACAGCAGTGATAGTAGTGACAGC
SHAP5:   ACAGCAGCAATAGCAGTAACAGCAGTGATAGTAGTGACAGCAGTGATAGCAGTGACAGCAGCAGTAGCAGTGACAGCAGCAGTAGCAGTGACAGCAGCAACAGCAGTGATAGTAGTGACAGTAGTGACAGCAGCAATAGCAGTGAGAGCAGTGATAGTAGTGACAGCAGTGATAGTGACAGCAGTGATAGTAGTGACAGC
SHAP6:   ACAGCAGCAATAGCAGTAACAGCAGTGATAGTAGTGACAGCAGTGATAGCAGTGACAGCAGCAGTAGCAGTGACAGCAGCAGTAGCAGTGACAGCAGCAACAGCAGTGATAGTAGTGACAGTAGTGACAGCAGCAATAGCAGTGAGAGCAGTGATAGTAGTGACAGCAGTGATAGTGACAGCAGTGATAGTAGTGACAGC
SHAP6(2) ACAGCAGCAATAGCAGTAACAGCAGTGATAGTAGTGACAGCAGTGATAGCAGTGACAGCAGCAGTAGCAGTGACAGCAGCAGTAGCAGTGACAGCAGCAACAGCAGTGATAGTAGTGACAGTAGTGACAGCAGCAATAGCAGTGAGAGCAGTGATAGTAGTGACAGCAGTGATAGTGACAGCAGTGATAGTAGTGACAGC
SHAP7    ACAGCAGCAATAGCAGTAACAGCAGTGATAGTAGTGACAGCAGTGATAGCAGTGACAGCAGCAGTAGCAGTGACAGCAGCA------------------ACAGCAGTGATAGTAGTGACAGTAGTGACAGCAGCAATAGCAGTGAGAGCAGTGATAGTAGTGACAGCAGTGATAGTGACAGCAGTGATAGTAGTGACAGC
SHAP102  ACAGCAGCAATAGCAGTAACAGCAGTGATAGTAGTGACAGCAGTGATAGCAGTGACAGCAGCAGTAGCAGTGACAGCAGCA------------------ACAGCAGTGATAGTAGTGACAGTAGTGACAGCAGCAATAGCAGTGAGAGCAGTGATAGTAGTGACAGCAGTGATAGTGACAGCAGTGATAGTAGTGACAGC
SHAP130  ACAGCAGCAATAGCAGTAACAGCAGTGATAGTAGTGACAGCAGTGATAGCAGTGACAGCAGCAGTAGCAGTGACAGCAGCAGTAGCAGTGACAGCAGCAACAGCAGTGATAGTAGTGACAGTAGTGACAGCAGCAATAGCAGTGAGAGCAGTGATAGTAGTGACAGCAGTGATAGTGACAGCAGTGATAGTAGTGACAGC
SHAP106  ACAGCAGCAATAGCAGTAACAGCAGTGATAGTAGTGACAGCAGTGATAGCAGTGACAGCAGCAGTAGCAGTGACAGCAGCAGTAGCAGTGACAGCAGCAACAGCAGTGATAGTAGTGACAGTAGTGACAGCAGCAATAGCAGTGAGAGCAGTGATAGTAGTGACAGCAGTGATAGTGACAGCAGTGATAGTAGTGACAGC
SHAP72   ACAGCAGCAATAGCAGTAACAGCAGTGATAGTAGTGACAGCAGTGATAGCAGTGACAGCAGCAGTAGCAGTGACAGCAGCA------------------ACAGCAGTGATAGTAGTGACAGTAGTGACAGCAGCAATAGCAGTGAGAGCAGTGATAGTAGTGACAGCAGTGATAGTGACAGCAGTGATAGTAGTGACAGC
SHAP110  ACAGCAGCAATAGCAGTAACAGCAGTGATAGTAGTGACAGCAGTGATAGCAGTGACAGCAGCAGTAGCAGTGACAGCAGCA------------------ACAGCAGTGATAGTAGTGACAGTAGTGACAGCAGCAATAGCAGTGAGAGCAGTGATAGTAGTGACAGCAGTGATAGTGACAGCAGTGATAGTAGTGACAGC
MERGED   ACAGCAGCAATAGCAGTAACAGCAGTGATAGTAGTGACAGCAGTGATAGCAGTGACAGCAGCAGTAGCAGTGACAGCAGCAGTAGCAGTGACAGCAGCAACAGCAGTGATAGTAGTGACAGTAGTGACAGCAGCAATAGCAGTGAGAGCAGTGATAGTAGTGACAGCAGTGATAGTGACAGCAGTGATAGTAGTGACAGC
TRANSL   D  S  S  N  S  S  N  S  S  D  S  S  D  S  S  D  S  S  D  S  S  S  S  S  D  S  S  S  S  S  D  S  S  N  S  S  D  S  S  D  S  S  D  S  S  N  S  S  E  S  S  D  S  S  D  S  S  D  S  D  S  S  D  S  S  D  S   718
```

Notes:

ID1: NM_014208.3:c.2035_2040del AGTAGC.

ID2: NM_014208.3:c.2053_2054insGTAGCAGTGACAGCAGCA.

Mutations:

8. g.11174delC; c.2040del**C**; p.(Ser680Argfs*634) [4]
9. g.11197delA; c.2063del**A**; p.(Asp688Valfs*626) [5]
10. g.11268delA; c.2134del**A**; p.(Ser712Alafs*602) [1,8]

```
      2155                                                                  ID3                                                                          2272                    ID4                                                                        2349 2354
REFSEQ    AGTAATAGTAACAGCAGCGATAGTGACAGCAGCAACAGCAGCGATAGCAGTGACAGCAGCAACAGCAGTGACAGCAGTGATAGCAGTGACAGCAGCAACAGCAGTGACAGTAGCGATAGCAGTGACAGCAGCAACAGCAGTGACAGCAGTGATAGCAGTGACAGCAGTGATAGTAGTGACAGCAGCAACAGCAGTGATAG
Fam8a     AGTAATAGTAACAGCAGCGATAGTGACAGCAGCAACAGCAGCGATAGCAGTGACAGCAGCAACAGCAGTGACAGCAGTGATAGCAGTGACAGCAGCAACAGCAGTGACAGTAGCGATAGCAGTGACAGCAGCAACAGCAGTGACAGCAGTGATAGCAGTGACAGCAGTGATAGTAGTGACAGCAGCAACAGCAGTGATAG
Fam8b     AGTAATAGTAACAGCAGCGATAGTGACAGCAGCAACAGCAGCGATAGCAGTGACAGCAGCAACAGCAGTGACAGCAGTGATAGCAGTGACAGCAGCAACAGCAGTGACAGTAGCGATAGCAGTGACAGCAGCAACAGCAGTGACAGCAGTGATAGCAGTGACAGCAGTGATAGTAGTGACAGCAGCAACAGCAGTGATAG
Fam9a     AGTAATAGTAACAGCAGCGATAGTGACAGCAGCAACAGCAGCGATAGCAGTGACAGCAGCAACAGCAGTGACAGCAGTGATAGCAGTGACAGCAGCAACAGCAGTGACAGTAGCGATAGCAGTGACAGCAGCAACAGCAGTGACAGCAGTGATAGCAGTGACAGCAGTGATAGTAGTGACAGCAGCAACAGCAGTGATAG
Fam9b     AGTAATAGTAACAGCAGCGATAGTGACAGCAGCAACAGCAGCGATAGCAGTGACAGCAGCAACAGCAGTGACAGCAGTGATAGCAGTGACAGCAGCAACAGCAGTGACAGTAGCGATAGCAGTGACAGCAGCAACAGCAGTGACAGCAGTGATAGCAGTGACAGCAGTGATAGTAGTGACAGCAGCAACAGCAGTGATAG
Fam10a    AGTAATAGTAACAGCAGCGATAGTGACAGCAGCAACAGCAGCGATAGCAGTGACAGCAGCAACAGCAGTGACAGCAGTGATAGCAGTGACAGCAGCAACAGCAGTGACAGTAGCGATAGCAGTGACAGCAGCAACAGCAGTGACAGCAGTGATAGCAGTGACAGCAGTGATAGTAGTGACAGCAGCAACAGCAGTGATAG
Fam10b    AGTAATAGTAACAGCAGCGATAGTGACAGCAGCAACAGCAGCGATAGCAGTGACAGCAGCAACAGCAGTGACAGCAGTGATAGCAGTGACAGCAGCAACAGCAGTGACAGTAGCGATAGCAGTGACAGCAGCAACAGCAGTGACAGCAGTGATAGCAGTGACAGCAGTGATAGTAGTGACAGCAGCAACAGCAGTGATAG
Fam11a    AGTAATAGTAACAGCAGCGATAGTGACAGCAGCAACAGCAGCGATAGCAGTGACAGCAGCAACAGCAGTGACAGCAGTGATAGCAGTGACAGCAGCAACAGCAGTGACAGTAGCGATAGCAGTGACAGCAGCAACAGCAGTGACAGCAGTGATAGCAGTGACAGCAGTGATAGTAGTGACAGCAGCAACAGCAGTGATAG
Fam11b    AGTAATAGTAACAGCAGCGATAGTGACAGCAGCAACAGCAGCGATAGCAGTGACAGCAGCAACAGCAGTGACAGCAGTGATAGCAGTGACAGCAGCAACAGCAGTGACAGTAGCGATAGCAGTGACAGCAGCAACAGCAGTGACAGCAGTGATAGCAGTGACAGCAGTGATAGTAGTGACAGCAGCAACAGCAGTGATAG
Fam12a    AGTAATAGTAACAGCAGCGATAGTGACAGCAGCAACAGCAGCGATAGCAGTGACAGCAGCAACAGCAGTGACAGCAGTGATAGCAGTGACAGCAGCAACAGCAGTGACAGTAGCGATAGCAGTGACAGCAGCAACAGCAGTGACAGCAGTGATAGCAGTGACAGCAGTGATAGTAGTGACAGCAGCAACAGCAGTGATAG
Fam12b    AGTAATAGTAACAGCAGCGATAGTGACAGCAGCAACAGCAGCGATAGCAGTGACAGCAGCAACAGCAGTGACAGCAGTGATAGCAGTGACAGCAGCAACAGCAGTGACAGTAGCGATAGCAGTGACAGCAGCAACAGCAGTGACAGCAGTGATAGCAGTGACAGCAGTGATAGTAGTGACAGCAGCAACAGCAGTGATAG
F1A       AGTAATAGTAACAGCAGCGATAGTGACAGCAGCAACAGCAGCGATAGCAGTGACAGCAGCAACAGCAGTGACAGCAGTGATAGCAGTGACAGCAGCAACAGCAGTGACAGTAGCGATAGCAGTGACAGCAGCAACAGCAGTGACAGCAGTGATAGCAGTGACAGCAGTGATAGTAGTGACAGCAGCAACAGCAGTGATAG
F1B       AGTAATAGTAACAGCAGCGATAGTGACAGCAGCAACAGCAGCGATAGCAGTGACAGCAG------------------TGATAGCAGTGACAGCAGCAACAGCAGTGACAGTAGCGATAGCAGTGACAGCAGCAACAGCAGTGACAGCAGTGATAGCAGTGACAGCAGTGATAGTAGTGACAGCAGCAACAGCAGTGATAG
F2A       AGTAATAGTAACAGCAGCGATAGTGACAGCAGCAACAGCAGCGATAGCAGTGACAGCAGCAACAGCAGTGACAGCAGTGATAGCAGTGACAGCAGCAACAGCAGTGACAGTAGCGATAGCAGTGACAGCAGCAACAGCAGTGACAGCAGTGATAGCAGTGACAGCAGTGATAGTAGTGACAGCAGCAACAGCAGTGATAG
F2B       AGTAATAGTAACAGCAGCGATAGTGACAGCAGCAACAGCAGCGATAGCAGTGACAGCAGCAACAGCAGTGACAGCAGTGATAGCAGTGACAGCAGCAACAGCAGTGACAGTAGCGATAGCAGTGACAGCAGCAACAGCAGTGACAGCAGTGATAGCAGTGACAGCAGTGATAGTAGTGACAGCAGCAACAGCAGTGATAG
F3A       AGTAATAGTAACAGCAGCGATAGTGACAGCAGCAACAGCAGCGATAGCAGTGACAGCAGCAACAGCAGTGACAGCAGTGATAGCAGTGACAGCAGCAACAGCAGTGACAGTAGCGATAGCAGTGACAGCAGCAACAGCAGTGACAGCAGTGATAGCAGTGACAGCAGTGATAGTAGTGACAGCAGCAACAGCAGTGATAG
F3B       AGTAATAGTAACAGCAGCGATAGTGACAGCAGCAACAGCAGCGATAGCAGTGACAGCAGCAACAGCAGTGACAGCAGTGATAGCAGTGACAGCAGCAACAGCAGTGACAGTAGCGATAGCAGTGACAGCAGCAACAGCAGTGACAGCAGTGATAGCAGTGACAGCAGTGATAGTAGTGACAGCAGCAACAGCAGTGATAG
F4A       AGTAATAGTAACAGCAGCGATAGTGACAGCAGCAACAGCAGCGATAGCAGTGACAGCAGCAACAGCAGTGACAGCAGTGATAGCAGTGACAGCAGCAACAGCAGTGACAGTAGCGATAGCAGTGACAGCAG------------------TGATAGCAGTGACAGCAGTGATAGTAGTGACAGCAGCAACAGCAGTGATAG
F4B       AGTAATAGTAACAGCAGCGATAGTGACAGCAGCAACAGCAGCGATAGCAGTGACAGCAGCAACAGCAGTGACAGCAGTGATAGCAGTGACAGCAGCAACAGCAGTGACAGTAGCGATAGCAGTGACAGCAGCAACAGCAGTGACAGCAGTGATAGCAGTGACAGCAGTGATAGTAGTGACAGCAGCAACAGCAGTGATAG
F5A       AGTAATAGTAACAGCAGCGATAGTGACAGCAGCAACAGCAGCGATAGCAGTGACAGCAGCAACAGCAGTGACAGCAGTGATAGCAGTGACAGCAGCAACAGCAGTGACAGTAGCGATAGCAGTGACAGCAGCAACAGCAGTGACAGCAGTGATAGCAGTGACAGCAGTGATAGTAGTGACAGCAGCAACAGCAGTGATAG
F5B       AGTAATAGTAACAGCAGCGATAGTGACAGCAGCAACAGCAGCGATAGCAGTGACAGCAGCAACAGCAGTGACAGCAGTGATAGCAGTGACAGCAGCAACAGCAGTGACAGTAGCGATAGCAGTGACAGCAGCAACAGCAGTGACAGCAGTGATAGCAGTGACAGCAGTGATAGTAGTGACAGCAGCAACAGCAGTGATAG
HAP1A     AGTAATAGTAACAGCAGCGATAGTGACAGCAGCAACAGCAGCGATAGCAGTGACAGCAG------------------TGATAGCAGTGACAGCAGCAACAGCAGTGACAGTAGCGATAGCAGTGACAGCAGCAACAGCAGTGACAGCAGTGATAGCAGTGACAGCAGTGATAGTAGTGACAGCAGCAACAGCAGTGATAG
HAP2A     AGTAATAGTAACAGCAGCGATAGTGACAGCAGCAACAGCAGCGATAGCAGTGACAGCAGCAACAGCAGTGACAGCAGTGATAGCAGTGACAGCAGCAACAGCAGTGACAGTAGCGATAGCAGTGACAGCAGCAACAGCAGTGACAGCAGTGATAGCAGTGACAGCAGTGATAGTAGTGACAGCAGCAACAGCAGTGATAG
HAP3A     AGTAATAGTAACAGCAGCGATAGTGACAGCAGCAACAGCAGCGATAGCAGTGACAGCAGCAACAGCAGTGACAGCAGTGATAGCAGTGACAGCAGCAACAGCAGTGACAGTAGCGATAGCAGTGACAGCAGCAACAGCAGTGACAGCAGTGATAGCAGTGACAGCAGTGATAGTAGTGACAGCAGCAACAGCAGTGATAG
HAP15A    AGTAATAGTAACAGCAGCGATAGTGACAGCAGCAACAGCAGCGATAGCAGTGACAGCAGCAACAGCAGTGACAGCAGTGATAGCAGTGACAGCAGCAACAGCAGTGACAGTAGCGATAGCAGTGACAGCAGCAACAGCAGTGACAGCAGTGATAGCAGTGACAGCAGTGATAGTAGTGACAGCAGCAACAGCAGTGATAG
HAP17B    AGTAATAGTAACAGCAGCGATAGTGACAGCAGCAACAGCAGCGATAGCAGTGACAGCAGCAACAGCAGTGACAGCAGTGATAGCAGTGACAGCAGCAACAGCAGTGACAGTAGCGATAGCAGTGACAGCAGCAACAGCAGTGACAGCAGTGATAGCAGTGACAGCAGTGATAGTAGTGACAGCAGCAACAGCAGTGATAG
HAP20A    AGTAATAGTAACAGCAGCGATAGTGACAGCAGCAACAGCAGCGATAGCAGTGACAGCAGCAACAGCAGTGACAGCAGTGATAGCAGTGACAGCAGCAACAGCAGTGACAGTAGCGATAGCAGTGACAGCAGCAACAGCAGTGACAGCAGTGATAGCAGTGACAGCAGTGATAGTAGTGACAGCAGCAACAGCAGTGATAG
HAP20B    AGTAATAGTAACAGCAGCGATAGTGACAGCAGCAACAGCAGCGATAGCAGTGACAGCAGCAACAGCAGTGACAGCAGTGATAGCAGTGACAGCAGCAACAGCAGTGACAGTAGCGATAGCAGTGACAGCAGCAACAGCAGTGACAGCAGTGATAGCAGTGACAGCAGTGATAGTAGTGACAGCAGCAACAGCAGTGATAG
HAP36A    AGTAATAGTAACAGCAGCGATAGTGACAGCAGCAACAGCAGCGATAGCAGTGACAGCAGCAACAGCAGTGACAGCAGTGATAGCAGTGACAGCAGCAACAGCAGTGACAGTAGCGATAGCAGTGACAGCAGCAACAGCAGTGACAGCAGTGATAGCAGTGACAGCAGTGATAGTAGTGACAGCAGCAACAGCAGTGATAG
HAP37A    AGTAATAGTAACAGCAGCGATAGTGACAGCAGCAACAGCAGCGATAGCAGTGACAGCAGCAACAGCAGTGACAGCAGTGATAGCAGTGACAGCAGCAACAGCAGTGACAGTAGCGATAGCAGTGACAGCAGCAACAGCAGTGACAGCAGTGATAGCAGTGACAGCAGTGATAGTAGTGACAGCAGCAACAGCAGTGATAG
HAP38A    AGTAATAGTAACAGCAGCGATAGTGACAGCAGCAACAGCAGCGATAGCAGTGACAGCAGCAACAGCAGTGACAGCAGTGATAGCAGTGACAGCAGCAACAGCAGTGACAGTAGCGATAGCAGTGACAGCAGCAACAGCAGTGACAGCAGTGATAGCAGTGACAGCAGTGATAGTAGTGACAGCAGCAACAGCAGTGATAG
SHAP1:    AGTAATAGTAACAGCAGCGATAGTGACAGCAGCAACAGCAGCGATAGCAGTGACAGCAGCAACAGCAGTGACAGCAGTGATAGCAGTGACAGCAGCAACAGCAGTGACAGTAGCGATAGCAGTGACAGCAGCAACAGCAGTGACAGCAGTGATAGCAGTGACAGCAGTGATAGTAGTGACAGCAGCAACAGCAGTGATAG
SHAP2:    AGTAATAGTAACAGCAGCGATAGTGACAGCAGCAACAGCAGCGATAGCAGTGACAGCAGCAACAGCAGTGACAGCAGTGATAGCAGTGACAGCAGCAACAGCAGTGACAGTAGCGATAGCAGTGACAGCAGCAACAGCAGTGACAGCAGTGATAGCAGTGACAGCAGTGATAGTAGTGACAGCAGCAACAGCAGTGATAG
SHAP3:    AGTAATAGTAACAGCAGCGATAGTGACAGCAGCAACAGCAGCGATAGCAGTGACAGCAGCAACAGCAGTGACAGCAGTGATAGCAGTGACAGCAGCAACAGCAGTGACAGTAGCGATAGCAGTGACAGCAGCAACAGCAGTGACAGCAGTGATAGCAGTGACAGCAGTGATAGTAGTGACAGCAGCAACAGCAGTGATAG
SHAP4:    AGTAATAGTAACAGCAGCGATAGTGACAGCAGCAACAGCAGCGATAGCAGTGACAGCAGCAACAGCAGTGACAGCAGTGATAGCAGTGACAGCAGCAACAGCAGTGACAGTAGCGATAGCAGTGACAGCAGCAACAGCAGTGACAGCAGTGATAGCAGTGACAGCAGTGATAGTAGTGACAGCAGCAACAGCAGTGATAG
SHAP5:    AGTAATAGTAACAGCAGCGATAGTGACAGCAGCAACAGCAGCGATAGCAGTGACAGCAGCAACAGCAGTGACAGCAGTGATAGCAGTGACAGCAGCAACAGCAGTGACAGTAGCGATAGCAGTGACAGCAGCAACAGCAGTGACAGCAGTGATAGCAGTGACAGCAGTGATAGTAGTGACAGCAGCAACAGCAGTGATAG
SHAP6:    AGTAATAGTAACAGCAGCGATAGTGACAGCAGCAACAGCAGCGATAGCAGTGACAGCAGCAACAGCAGTGACAGCAGTGATAGCAGTGACAGCAGCAACAGCAGTGACAGTAGCGATAGCAGTGACAGCAGCAACAGCAGTGACAGCAGTGATAGCAGTGACAGCAGTGATAGTAGTGACAGCAGCAACAGCAGTGATAG
SHAP6(2)  AGTAATAGTAACAGCAGCGATAGTGACAGCAGCAACAGCAGCGATAGCAGTGACAGCAGCAACAGCAGTGACAGCAGTGATAGCAGTGACAGCAGCAACAGCAGTGACAGTAGCGATAGCAGTGACAGCAGCAACAGCAGTGACAGCAGTGATAGCAGTGACAGCAGTGATAGTAGTGACAGCAGCAACAGCAGTGATAG
SHAP7     AGTAATAGTAACAGCAGCGATAGTGACAGCAGCAACAGCAGCGATAGCAGTGACAGCAGCAACAGCAGTGACAGCAGTGATAGCAGTGACAGCAGCAACAGCAGTGACAGTAGCGATAGCAGTGACAGCAGCAACAGCAGTGACAGCAGTGATAGCAGTGACAGCAGTGATAGTAGTGACAGCAGCAACAGCAGTGATAG
SHAP102   AGTAATAGTAACAGCAGCGATAGTGACAGCAGCAACAGCAGCGATAGCAGTGACAGCAGCAACAGCAGTGACAGCAGTGATAGCAGTGACAGCAGCAACAGCAGTGACAGTAGCGATAGCAGTGACAGCAGCAACAGCAGTGACAGCAGTGATAGCAGTGACAGCAGTGATAGTAGTGACAGCAGCAACAGCAGTGATAG
SHAP130   AGTAATAGTAACAGCAGCGATAGTGACAGCAGCAACAGCAGCGATAGCAGTGACAGCAGCAACAGCAGTGACAGCAGTGATAGCAGTGACAGCAGCAACAGCAGTGACAGTAGCGATAGCAGTGACAGCAGCAACAGCAGTGACAGCAGTGATAGCAGTGACAGCAGTGATAGTAGTGACAGCAGCAACAGCAGTGATAG
SHAP106   AGTAATAGTAACAGCAGCGATAGTGACAGCAGCAACAGCAGCGATAGCAGTGACAGCAGCAACAGCAGTGACAGCAGTGATAGCAGTGACAGCAGCAACAGCAGTGACAGTAGCGATAGCAGTGACAGCAGCAACAGCAGTGACAGCAGTGATAGCAGTGACAGCAGTGATAGTAGTGACAGCAGCAACAGCAGTGATAG
SHAP72    AGTAATAGTAACAGCAGCGATAGTGACAGCAGCAACAGCAGCGATAGCAGTGACAGCAGCAACAGCAGTGACAGCAGTGATAGCAGTGACAGCAGCAACAGCAGTGACAGTAGCGATAGCAGTGACAGCAGCAACAGCAGTGACAGCAGTGATAGCAGTGACAGCAGTGATAGTAGTGACAGCAGCAACAGCAGTGATAG
SHAP110   AGTAATAGTAACAGCAGCGATAGTGACAGCAGCAACAGCAGCGATAGCAGTGACAGCAGCAACAGCAGTGACAGCAGTGATAGCAGTGACAGCAGCAACAGCAGTGACAGTAGCGATAGCAGTGACAGCAGCAACAGCAGTGACAGCAGTGATAGCAGTGACAGCAGTGATAGTAGTGACAGCAGCAACAGCAGTGATAG
MERGED    AGTAATAGTAACAGCAGCGATAGTGACAGCAGCAACAGCAGCGATAGCAGTGACAGCAGCAACAGCAGTGACAGCAGTGATAGCAGTGACAGCAGCAACAGCAGTGACAGTAGCGATAGCAGTGACAGCAGCAACAGCAGTGACAGCAGTGATAGCAGTGACAGCAGTGATAGTAGTGACAGCAGCAACAGCAGTGATAG
TRANSL     S  N  S  N  S  S  D  S  D  S  S  N  S  S  D  S  S  D  S  S  N  S  S  D  S  S  D  S  S  D  S  S  N  S  S  D  S  S  D  S  S  D  S  S  N  S  S  D  S  S  D  S  S  D  S  S  D  S  S  D  S  S  N  S  S  D  S   785
```

Notes:

ID3: NM_014208.3:c.2214_2231delCAACAGCAGTGACAGCAG.

ID4: NM_014208.3:c.2286_2303delCAACAGCAGTGACAGCAG.

Mutations:

11. g.11406delA; c.2272del**A**; p.(Ser758Alafs*556) [2]
12. g.11483delT; c.2349del**T**; p.(Ser783Argfs*531) [5]

```
         2355                                                                                                                                                                 ID5     ID6     2525                        2554
REFSEQ   CAACGACAGCAGCAATAGCAGTGACAGCAGTGATAGCAGCAACAGCAGTGATAGCAGCAACAGCAGTGATAGCAGTGATAGCAGTGACAGCAGTGATAGCGACAGCAGCAATAGCAGTGACAGCAGTAATAGTAGTGACAGCAGCGATAGCAGCAACAGCAGTGATAGCAGCGACAGCAGCGATAGCAGTGACGGCAGTG
Fam8a    CAACGACAGCAGCAATAGCAGTGACAGCAGTGATAGCAGCAACAGCAGTGATAGCAGCAACAGCAGTGATAGCAGTGATAGCAGTGACAGCAGTGATAGCGACAGCAGCAATAGCAGTGACAGCAGTAATAGTAGTGACAGCAGCGATAGCAGCAACAGCAGTGATAGCAGCGACAGCAGCGATAGCAGTGACAGCAGTG
Fam8b    CAACGACAGCAGCAATAGCAGTGACAGCAGTGATAGCAGCAACAGCAGTGATAGCAGCAACAGCAGTGATAGCAGTGATAGCAGTGACAGCAGTGATAGCGACAGCAGCAATAGCAGTGACAGCAGTAATAGTAGTGACAGCAGCGATAGCAGCAACAGCAGTGATAGCA-CGACAGCAGCGATAGCAGTGACGGCAGTG
Fam9a    CAACGACAGCAGCAATAGCAGTGACAGCAGTGATAGCAGCAACAGCAGTGATAGCAGCAACAGCAGTGATAGCAGTGATAGCAGTGACAGCAGTGATAGCGACAGCAGCAATAGCAGTGACAGCAGTAATAGTAGTGACAGCAGCGATAGCAGCAACAGCAGTGATAGCA-CGACAGCAGCGATAGCAGTGACGGCAGTG
Fam9b    CAACGACAGCAGCAATAGCAGTGACAGCAGTGATAGCAGCAACAGCAGTGATAGCAGCAACAGCAGTGATAGCAGTGATAGCAGTGACAGCAGTGATAGCGACAGCAGCAATAGCAGTGACAGCAGTAATAGTAGTGACAGCAGCGATAGCA---ACAGCAGTGATAGCAGCGACAGCAGCGATAGCAGTGACAGCAGTG
Fam10a   CAACGACAGCAGCAATAGCAGTGACAGCAGTGATAGCAGCAACAGCAGTGATAGCAGCAACAGCAGTGATAGCAGTGATAGCAGTGACAGCAGTGATAGCGACAGCAGCAATAGCAGTGACAGCAGTAATAGTAGTGACAGCAGCGATAGCA---ACAGCAGTGATAGCAGCGACAGCAGCGATAGCAGTGACAGCAGTG
Fam10b   CAACGACAGCAGCAATAGCAGTGACAGCAGTGATAGCAGCAACAGCAGTGATAGCAGCAACAGCAGTGATAGCAGTGATAGCAGTGACAGCAGTGATAGCGACAGCAGCAATAGCAGTGACAGCAGTAATAGTAGTGACAGCAGCGATAGCAGCAACAGCAGTGATAGCAGCGACAGCAGCGATAGCAGTGACAGCAGTG
Fam11a   CAACGACAGCAGCAATAGCAGTGACAGCAGTGATAGCAGCAACAGCAGTGATAGCAGCAACAGCAGTGATAGCAGTGATAGCAGTGACAGCAGTGATAGCGACAGCAGCAATAGCAGTGACAGCAGTAATAGTAGTGACAGCAGCGATAGCAGCAACAGCAGTGATAGCAGCGACAGCAGCGATAGCAGTGACAGCAGTG
Fam11b   CAACGACAGCAGCAATAGCAGTGACAGCAGTGATAGCAGCAACAGCAGTGATAGCAGCAACAGCAGTGATAGCAGTGATAGCAGTGACAGCAGTGATAGCGACAGCAGCAATAGCAGTGACAGCAGTAATAGTAGTGACAGCAGCGATAGCAGCAACAGCAGTGATAGCAGCGACAGCAGCGATAGCAGTGACAGCAGTG
Fam12a   CAACGACAGCAGCAATAGCAGTGACAGCAGTGATAGCAGCAACAGCAGTGATAGCAGCAACAGCAGTGATAGCAGTGATAGCAGTGACAGCAGTGATAGCGACAGCAGCAATAGCAGTGACAGCAGTAATAGTAGTGACAGCAGCGATAGCA---ACAGCAGTGATAGCAGCGACAGCAGCGATAGCAGTGACAGCAGTG
Fam12b   CAACGACAGCAGCAATAGCAGTGACAGCAGTGATAGCAGCAACAGCAGTGATAGCAGCAACAGCAGTGATAGCAGTGATAGCAGTGACAGCAGTGATAGCGACAGCAGCAATAGCAGTGACAGCAGTAATAGTAGTGACAGCAGCGATAGCAGCAACAGCAGTGATAGCAGCGACAGCAGCGATAGCAGTGACAGCAGTG
F1A      CAACGACAGCAGCAATAGCAGTGACAGCAGTGATAGCAGCAACAGCAGTGATAGCAGCAACAGCAGTGATAGCAGTGATAGCAGTGACAGCAGTGATAGCGACAGCAGCAATAGCAGTGACAGCAGTAATAGTAGTGACAGCAGCGATAGCA---ACAGCAGTGATAGCAGCGACAGCAGCGATAGCAGTGACAGCAGTG
F1B      CAACGACAGCAGCAATAGCAGTGACAGCAGTGATAGCAGCAACAGCAGTGATAGCAGCAACAGCAGTGATAGCAGTGATAGCAGTGACAGCAGTGATAGCGACAGCAGCAATAGCAGTGACAGCAGTAATAGTAGTGACAGCAGCGATAGCAGCAACAGCAGTGATAGCAGCGACAGCAGCGATAGCAGTGACAGCAGTG
F2A      CAACGACAGCAGCAATAGCAGTGACAGCAGTGATAGCAGCAACAGCAGTGATAGCAGCAACAGCAGTGATAGCAGTGATAGCAGTGACAGCAGTGATAGCGACAGCAGCAATAGCAGTGACAGCAGTAATAGTAGTGACAGCAGCGATAGCAGCAACAGCAGTGATAGCAGCGACAGCAGCGATAGCAGTGACAGCAGTG
F2B      CAACGACAGCAGCAATAGCAGTGACAGCAGTGATAGCAGCAACAGCAGTGATAGCAGCAACAGCAGTGATAGCAGTGATAGCAGTGACAGCAGTGATAGCGACAGCAGCAATAGCAGTGACAGCAGTAATAGTAGTGACAGCAGCGATAGCAGCAACAGCAGTGATAGCAGCGACAGCAGCGATAGCAGTGACGGCAGTG
F3A      CAACGACAGCAGCAATAGCAGTGACAGCAGTGATAGCAGCAACAGCAGTGATAGCAGCAACAGCAGTGATAGCAGTGATAGCAGTGACAGCAGTGATAGCGACAGCAGCAATAGCAGTGACAGCAGTAATAGTAGTGACAGCAGCGATAGCAGCAACAGCAGTGATAGCAGCGACAGCAGCGATAGCAGTGACAGCAGTG
F3B      CAACGACAGCAGCAATAGCAGTGACAGCAGTGATAGCAGCAACAGCAGTGATAGCAGCAACAGCAGTGATAGCAGTGATAGCAGTGACAGCAGTGATAGCGACAGCAGCAATAGCAGTGACAGCAGTAATAGTAGTGACAGCAGCGATAGCAGCAACAGCAGTGATAGCAGCGACAGCAGCGATAGCAGTGACGGCAGTG
F4A      CAACGACAGCAGCAATAGCAGTGACAGCAGTGATAGCAGCAACAGCAGTGATAGCAGCAACAGCAGTGATAGCAGTGATAGCAGTGACAGCAGTGATAGCGACAGCAGCAATAGCAGTGACAGCAGTAATAGTAGTGACAGCAGCGATAGCAGC------------------GACAGCAGAGATAGCAGTGACAGCAGTG
F4B      CAACGACAGCAGCAATAGCAGTGACAGCAGTGATAGCAGCAACAGCAGTGATAGCAGCAACAGCAGTGATAGCAGTGATAGCAGTGACAGCAGTGATAGCGACAGCAGCAATAGCAGTGACAGCAGTAATAGTAGTGACAGCAGCGATAGCAGCAACAGCAGTGATAGCAGCGACAGCAGCGATAGCAGTGACGGCAGTG
F5A      CAACGACAGCAGCAATAGCAGTGACAGCAGTGATAGCAGCAACAGCAGTGATAGCAGCAACAGCAGTGATAGCAGTGATAGCAGTGACAGCAGTGATAGCGACAGCAGCAATAGCAGTGACAGCAGTAATAGTAGTGACAGCAGCGATAGCAGCAACAGCAGTGATAGCAGCGACAGCAGCGATAGCAGTGACGGCAGTG
F5B      CAACGACAGCAGCAATAGCAGTGACAGCAGTGATAGCAGCAACAGCAGTGATAGCAGCAACAGCAGTGATAGCAGTGATAGCAGTGACAGCAGTGATAGCGACAGCAGCAATAGCAGTGACAGCAGTAATAGTAGTGACAGCAGCGATAGCAGCAACAGCAGTGATAGCAGCGACAGCAGCGATAGCAGTGACAGCAGTG
HAP1A    CAACGACAGCAGCAATAGCAGTGACAGCAGTGATAGCAGCAACAGCAGTGATAGCAGCAACAGCAGTGATAGCAGTGATAGCAGTGACAGCAGTGATAGCGACAGCAGCAATAGCAGTGACAGCAGTAATAGTAGTGACAGCAGCGATAGCAGCAACAGCAGTGATAGCAGCGACAGCAGCGATAGCAGTGACAGCAGTG
HAP2A    CAACGACAGCAGCAATAGCAGTGACAGCAGTGATAGCAGCAACAGCAGTGATAGCAGCAACAGCAGTGATAGCAGTGATAGCAGTGACAGCAGTGATAGCGACAGCAGCAATAGCAGTGACAGCAGTAATAGTAGTGACAGCAGCGATAGCAGCAACAGCAGTGATAGCAGCGACAGCAGCGATAGCAGTGACGGCAGTG
HAP3A    CAACGACAGCAGCAATAGCAGTGACAGCAGTGATAGCAGCAACAGCAGTGATAGCAGCAACAGCAGTGATAGCAGTGATAGCAGTGACAGCAGTGATAGCGACAGCAGCAATAGCAGTGACAGCAGTAATAGTAGTGACAGCAGCGATAGCAGCAACAGCAGTGATAGCAGCGACAGCAGCGATAGCAGTGACGGCAGTG
HAP15A   CAACGACAGCAGCAATAGCAGTGACAGCAGTGATAGCAGCAACAGCAGTGATAGCAGCAACAGCAGTGATAGCAGTGATAGCAGTGACAGCAGTGATAGCGACAGCAGCAATAGCAGTGACAGCAGTAATAGTAGTGACAGCAGCGATAGCAGCAACAGCAGTGATAGCAGCGACAGCAGCGATAGCAGTGACAGCAGTG
HAP17B   CAACGACAGCAGCAATAGCAGTGACAGCAGTGATAGCAGCAACAGCAGTGATAGCAGCAACAGCAGTGATAGCAGTGATAGCAGTGACAGCAGTGATAGCGACAGCAGCAATAGCAGTGACAGCAGTAATAGTAGTGACAGCAGCGATAGCA---ACAGCAGTGATAGCAGCGACAGCAGCGATAGCAGTGACAGCAGTG
HAP20A   CAACGACAGCAGCAATAGCAGTGACAGCAGTGATAGCAGCAACAGCAGTGATAGCAGCAACAGCAGTGATAGCAGTGATAGCAGTGACAGCAGTGATAGCGACAGCAGCAATAGCAGTGACAGCAGTAATAGTAGTGACAGCAGCGATAGCAGCAACAGCAGTGATAGCAGCGACAGCAGCGATAGCAGTGACGGCAGTG
HAP20B   CAACGACAGCAGCAATAGCAGTGACAGCAGTGATAGCAGCAACAGCAGTGATAGCAGCAACAGCAGTGATAGCAGTGATAGCAGTGACAGCAGTGATAGCGACAGCAGCAATAGCAGTGACAGCAGTAATAGTAGTGACAGCAGCGATAGCAGCAACAGCAGTGATAGCAGCGACAGCAGCGATAGCAGTGACGGCAGTG
HAP36A   CAACGACAGCAGCAATAGCAGTGACAGCAGTGATAGCAGCAACAGCAGTGATAGCAGCAACAGCAGTGATAGCAGTGATAGCAGTGACAGCAGTGATAGCGACAGCAGCAATAGCAGTGACAGCAGTAATAGTAGTGACAGCAGCGATAGCAGCAACAGCAGTGATAGCAGCGACAGCAGCGATAGCAGTGACGGCAGTG
HAP37A   CAACGACAGCAGCAATAGCAGTGACAGCAGTGATAGCAGCAACAGCAGTGATAGCAGCAACAGCAGTGATAGCAGTGATAGCAGTGACAGCAGTGATAGCGACAGCAGCAATAGCAGTGACAGCAGTAATAGTAGTGACAGCAGCGATAGCAGCAACAGCAGTGATAGCAGCGACAGCAGCGATAGCAGTGACAGCAGTG
HAP38A   CAACGACAGCAGCAATAGCAGTGACAGCAGTGATAGCAGCAACAGCAGTGATAGCAGCAACAGCAGTGATAGCAGTGATAGCAGTGACAGCAGTGATAGCGACAGCAGCAATAGCAGTGACAGCAGTAATAGTAGTGACAGCAGCGATAGCAGCAACAGCAGTGATAGCAGCGACAGCAGCGATAGCAGTGACGGCAGTG
SHAP1:   CAACGACAGCAGCAATAGCAGTGACAGCAGTGATAGCAGCAACAGCAGTGATAGCAGCAACAGCAGTGATAGCAGTGATAGCAGTGACAGCAGTGATAGCGACAGCAGCAATAGCAGTGACAGCAGTAATAGTAGTGACAGCAGCGATAGCAGCAACAGCAGTGATAGCAGCGACAGCAGCGATAGCAGTGACAGCAGTG
SHAP2:   CAACGACAGCAGCAATAGCAGTGACAGCAGTGATAGCAGCAACAGCAGTGATAGCAGCAACAGCAGTGATAGCAGTGATAGCAGTGACAGCAGTGATAGCGACAGCAGCAATAGCAGTGACAGCAGTAATAGTAGTGACAGCAGCGATAGCAGCAACAGCAGTGATAGCAGCGACAGCAGCGATAGCAGTGACGGCAGTG
SHAP3:   CAACGACAGCAGCAATAGCAGTGACAGCAGTGATAGCAGCAACAGCAGTGATAGCAGCAACAGCAGTGATAGCAGTGATAGCAGTGACAGCAGTGATAGCGACAGCAGCAATAGCAGTGACAGCAGTAATAGTAGTGACAGCAGCGATAGCAGCAACAGCAGTGATAGCAGCGACAGCAGCGATAGCAGTGACAGCAGTG
SHAP4:   CAACGACAGCAGCAATAGCAGTGACAGCAGTGATAGCAGCAACAGCAGTGATAGCAGCAACAGCAGTGATAGCAGTGATAGCAGTGACAGCAGTGATAGCGACAGCAGCAATAGCAGTGACAGCAGTAATAGTAGTGACAGCAGCGATAGCAGCAACAGCAGTGATAGCAGCGACAGCAGCGATAGCAGTGACGGCAGTG
SHAP5:   CAACGACAGCAGCAATAGCAGTGACAGCAGTGATAGCAGCAACAGCAGTGATAGCAGCAACAGCAGTGATAGCAGTGATAGCAGTGACAGCAGTGATAGCGACAGCAGCAATAGCAGTGACAGCAGTAATAGTAGTGACAGCAGCGATAGCA---ACAGCAGTGATAGCAGCGACAGCAGCGATAGCAGTGACAGCAGTG
SHAP6:   CAACGACAGCAGCAATAGCAGTGACAGCAGTGATAGCAGCAACAGCAGTGATAGCAGCAACAGCAGTGATAGCAGTGATAGCAGTGACAGCAGTGATAGCGACAGCAGCAATAGCAGTGACAGCAGTAATAGTAGTGACAGCAGCGATAGCAGCAACAGCAGTGATAGCAGCGACAGCAGCGATAGCAGTGACAGCAGTG
SHAP6(2) CAACGACAGCAGCAATAGCAGTGACAGCAGTGATAGCAGCAACAGCAGTGATAGCAGCAACAGCAGTGATAGCAGTGATAGCAGTGACAGCAGTGATAGCGACAGCAGCAATAGCAGTGACAGCAGTAATAGTAGTGACAGCAGCGATAGCA---ACAGCAGTGATAGCAGCGACAGCAGCGATAGCAGTGACAGCAGTG
SHAP7    CAACGACAGCAGCAATAGCAGTGACAGCAGTGATAGCAGCAACAGCAGTGATAGCAGCAACAGCAGTGATAGCAGTGATAGCAGTGACAGCAGTGATAGCGACAGCAGCAATAGCAGTGACAGCAGTAATAGTAGTGACAGCAGCGATAGCAGCAACAGCAGTGATAGCAGCGACAGCAGCGATAGCAGTGACGGCAGTG
SHAP102  CAACGACAGCAGCAATAGCAGTGACAGCAGTGATAGCAGCAACAGCAGTGATAGCAGCAACAGCAGTGATAGCAGTGATAGCAGTGACAGCAGTGATAGCGACAGCAGCAATAGCAGTGACAGCAGTAATAGTAGTGACAGCAGCGATAGCAGCAACAGCAGTGATAGCAGCGACAGCAGCGATAGCAGTGACAGCAGTG
SHAP130  CAACGACAGCAGCAATAGCAGTGACAGCAGTGATAGCAGCAACAGCAGTGATAGCAGCAACAGCAGTGATAGCAGTGATAGCAGTGACAGCAGTGATAGCGACAGCAGCAATAGCAGTGACAGCAGTAATAGTAGTGACAGCAGCGATAGCAGCAACAGCAGTGATAGCAGCGACAGCAGCGATAGCAGTGACAGCAGTG
SHAP106  CAACGACAGCAGCAATAGCAGTGACAGCAGTGATAGCAGCAACAGCAGTGATAGCAGCAACAGCAGTGATAGCAGTGATAGCAGTGACAGCAGTGATAGCGACAGCAGCAATAGCAGTGACAGCAGTAATAGTAGTGACAGCAGCGATAGCAGCAACAGCAGTGATAGCAGCGACAGCAGCGATAGCAGTGACAGCAGTG
SHAP72   CAACGACAGCAGCAATAGCAGTGACAGCAGTGATAGCAGCAACAGCAGTGATAGCAGCAACAGCAGTGATAGCAGTGATAGCAGTGACAGCAGTGATAGCGACAGCAGCAATAGCAGTGACAGCAGTAATAGTAGTGACAGCAGCGATAGCAGCAACAGCAGTGATAGCAGCGACAGCAGCGATAGCAGTGACAGCAGTG
SHAP110  CAACGACAGCAGCAATAGCAGTGACAGCAGTGATAGCAGCAACAGCAGTGATAGCAGCAACAGCAGTGATAGCAGTGATAGCAGTGACAGCAGTGATAGCGACAGCAGCAATAGCAGTGACAGCAGTAATAGTAGTGACAGCAGCGATAGCAGCAACAGCAGTGATAGCAGCGACAGCAGCGATAGCAGTGACGGCAGTG
MERGED   CAACGACAGCAGCAATAGCAGTGACAGCAGTGATAGCAGCAACAGCAGTGATAGCAGCAACAGCAGTGATAGCAGTGATAGCAGTGACAGCAGTGATAGCGACAGCAGCAATAGCAGTGACAGCAGTAATAGTAGTGACAGCAGCGATAGCAGCAACAGCAGTGATAGCAGCGACAGCAGCGATAGCAGTGACGGCAGTG
TRANSL     N  D  S  S  N  S  S  D  S  S  D  S  S  N  S  S  D  S  S  N  S  S  D  S  S  D  S  S  D  S  S  D  S  D  S  S  N  S  S  D  S  S  N  S  S  D  S  S  D  S  S  N  S  S  D  S  S  D  S S/R D  S  S  D G/S S    851
```

Notes:

SNP: c.2535C>A or p.Ser845Arg; c.2535C>A; c.2548G>A (p.Gly850Ser).

ID5: NM_014208.3:c.2507_2509delGCA.

ID6: NM_014208.3:c.2509_2526delAACAGCAGTGATAGCAGC.

Mutations:

13. g.11659delG; c.2525del**G**; p.(Ser842Thrfs*472); Fam 8&9 [2]

	2555 *ID7* 2593 *ID8* 2666 2684 2688 *ID9* 2745
REFSEQ	ATAGCGACAGCAGCAATAGAAGTGACAGTAGTAATAGT**A**GTGACAGCAGCGATAGCAGTGACAGCAGCAACAGCAGTGACAGCAGTGATAG---------CAGTGACAGCAACGAAAGCA**G**CAATAGCAGTGACAGCA**G**TGA**T**AGCAGCAACAGCAGTGATAGTGACAGCAGTGATAGCAGCAACAGCAGTGACAGCAGT
Fam8a	ATAGCGACAGCAGCAATAGAAGTGACAGTAGTAATAGTAGTGACAGCAGCGATAGCAGTGACAGCAGCAACAGCAGTGACAGCAGTGATAGTAGTGACAGCAGTGACAGCAACGAAAGCAGCAATAGCAGTGACAGCAGTGATAGCAGCAACAGCAGTGATAGTGACAGCAGTGATAGCAGCAACAGCAG**C**GACAGCAGT
Fam8b	ATAGCGACAGCAGCAATAGAAGTGACAGTAGTAATAGTAGTGACAGCAGCGATAGCAGTGACAGCAGCAACAGCAGTGACAGCAGTGATAG---------CAGTGACAGCAACGAAAGCAGCAATAGCAGTGACAGCAGTGATAGCAGCAACAGCAGTGACAGTGACAGCAGTGATAGCAGCAACAGCAGTGACAGCAGT
Fam9a	ATAGCGACAGCAGCAATAGAAGTGACAGTAGTAATAGTAGTGACAGCAGCGATAGCAGTGACAGCAGCAACAGCAGTGACAGCAGTGATAG---------CAGTGACAGCAACGAAAGCAGCAATAGCAGTGACAGCAGTGATAGCAGCAACAGCAGTGACAGTGACAGCAGTGATAGCAGCAACAGCAGTGACAGCAGT
Fam9b	ATAGCGACAGCAGCAATAGAAGTGACAGTAGTAATAGTAGTGACAGCAGCGATAGCAGTGACAGCAGCAACAGCAGTGACAGCAGTGATAGTAGTGACAGCAGTGACAGCAACGAAAGCAGCAATAGCAGTGACAGCAGTGATAGCAGCAACAGCAGTGATAGTGACAGCAGTGATAGCAGCAACAGCAGTGACAGCAGT
Fam10a	ATAGCGACAGCAGCAATAGAAGTGACAGTAGTAATAGTAGTGACAGCAGCGATAGCAGTGACAGCAGCAACAGCAGTGACAGCAGTGATAGTAGTGACAGCAGTGACAGCAACGAAAGCAGCAATAGCAGTGACAGCAGTGATAGCAGCAACAGCAGTGATAGTGACAGCAGTGATAGCAGCAACAGCAGTGACAGCAGT
Fam10b	ATAGCGACAGCAGCAATAGAAGTGACAGTAGTAATAGTAGTGACAGCAGCGATAGCAGTGACAGCAGCAACAGCAGTGACAGCAGTGATAGTAGTGACAGCAGTGACAGCAACGAAAGCAGCAATAGCAGTGACAGCAGTGATAGCAGCAACAGCAGTGATAGTGACAGCAGTGATAGCAGCAACAGCAG**C**GACAGCAGT
Fam11a	ATAGCGACAGCAGCAATAGAAGTGACAGTAGTAATAGTAGTGACAGCAGCGATAGCAGTGACAGCAGCAACAGCAGTGACAGCAGTGATAGTAGTGACAGCAGTGACAGCAACGAAAGCAGCAATAGCAGTGACAGCAGTGATAGCAGCAACAGCAGTGATAGTGACAGCAGTGATAGCAGCAACAGCAGCGACAGCAGT
Fam11b	ATAGCGACAGCAGCAATAGAAGTGACAGTAGTAATAGTAGTGACAGCAGCGATAGCAGTGACAGCAGCAACAGCAGTGACAGCAGTGATAGTAGTGACAGCAGTGACAGCAACGAAAGCAGCAATAGCAGTGACAGCAGTGATAGCAGCAACAGCAGTGATAGTGACAGCAGTGATAGCAGCAACAGCAGCGACAGCAGT
Fam12a	ATAGCGACAGCAGCAATAGAAGTGACAGTAGTAATAGTAGTGACAGCAGCGATAGCAGTGACAGCAGCAACAGCAGTGACAGCAGTGATAGTAGTGACAGCAGTGACAGCAACGAAAGCAGCAATAGCAGTGACAGCAGTGATAGCAGCAACAGCAGTGATAGTGACAGCAGTGATAGCAGCAACAGCAGTGACAGCAGT
Fam12b	ATAGCGACAGCAGCAATAGAAGTGACAGTAGTAATAGTAGTGACAGCAGCGATAGCAGTGACAGCAGCAACAGCAGTGACAGCAGTGATAGTAGTGACAGCAGTGACAGCAACGAAAGCAGCAATAGCAGTGACAGCAGTGATAGCAGCAACAGCAGTGATAGTGACAGCAGTGATAGCAGCAACAGCAG**C**GACAGCAGT
F1A	ATAGCGACAGCAGCAATAGAAGTGACAGTAGTAATAGTAGTGACAGCAGCGATAGCAGTGACAGCAGCAACAGCAGTGACAGCAGTGATAGTAGTGACAGCAGTGACAGCAACGAAAGCAGCAATAGCAGTGACAGCAGTGATAGCAGCAACAGCAGTGATAGTGACAGCAGTGATAGCAGCAACAGCAGTGACAGCAGT
F1B	ATAGCGACAGCAGCAATAGAAGTGACAGTAGTAATAGTAGTGACAGCAGCGATAGCAGTGACAGCAGCAACAGCAGTGACAGCAGTGATAGTAGTGACAGCAGTGACAGCAACGAAAGCAGCAATAGCAGTGACAGCAGTGATAGCAGCAACAGCAGTGATAGTGACAGCAGTGATAGCAGCAACAGCAGTGACAGCAGT
F2A	ATAGCGACAGCAGCAATAGAAGTGACAGTAGTAATAGTAGTGACAGCAGCGATAGCAGTGACAGCAGCAACAGCAGTGACAGCAGTGATAGTAGTGACAGCAGTGACAGCAACGAAAGCAGCAATAGCAGTGACAGCAGTGATAGCAGCAACAGCAGTGATAGTGACAGCAGTGATAGCAGCAACAGCAG**C**GACAGCAGT
F2B	ATAGCGACAGCAGCAATAGAAGTGACAGTAGTAATAGTAGTGACAGCAGCGATAGCAGTGACAGCAGCAACAGCAGTGACAGCAGTGATAG---------CAGTGACAGCAACGAAAGCAGCAATAGCAGTGACAGCAGTGATAGCAGCAACAGCAGTGACAGTGACAGCAGTGATAGCAGCAACAGCAGTGACAGCAGT
F3A	ATAGCGACAGCAGCAATAGAAGTGACAGTAGTAATAGTAGTGACAGCAGCGATAGCAGTGACAGCAGCAACAGCAGTGACAGCAGTGATAGTAGTGACAGCAGTGACAGCAACGAAAGCAGCAATAGCAGTGACAGCAGTGATAGCAGCAACAGCAGTGATAGTGACAGCAGTGATAGCAGCAACAGCAGCGACAGCAGT
F3B	ATAGCGACAGCAGCAATAGAAGTGACAGTAGTAATAGTAGTGACAGCAGCGATAGCAGTGACAGCAGCAACAGCAGTGACAGCAGTGATAG---------CAGTGACAGCAACGAAAGCAGCAATAGCAGTGACAGCAGTGATAGCAGCAACAGCAGTGACAGTGACAGCAGTGATAGCAGCAACAGCAGTGACAGCAGT
F4A	ATAGCGACAGCAGCAATAGAAGTGACAGTAGTAATAGTAGTGACAGCAGCGATAGCAGTGACAGCAGCAACAGCAGTGACAGCAGTGATAGTAGTGACAGCAGTGACAGCAACGAAAGCAGCAATAGCAGTGACAGCAGTGATAGCAGCAACAGCAGTGATAGTGACAGCAGTGATAGCAGCAACAGCAGTGACAGCAGT
F4B	ATAGCGACAGCAGCAATAGAAGTGACAGTAGTAATAGTAGTGACAGCAGCGATAGCAGTGACAGCAGCAACAGCAGTGACAGCAGTGATAG---------CAGTGACAGCAACGAAAGCAGCAATAGCAGTGACAGCAGTGATAGCAGCAACAGCAGTGACAGTGACAGCAGTGATAGCAGCAACAGCAGTGACAGCAGT
F5A	ATAGCGACAGCAGCAATAGAAGTGACAGTAGTAATAGTAGTGACAGCAGCGATAGCAGTGACAGCAGCAACAGCAGTGACAGCAGTGATAG---------CAGTGACAGCAACGAAAGCAGCAATAGCAGTGACAGCAGTGATAGCAGCAACAGCAGTGACAGTGACAGCAGTGATAGCAGCAACAGCAGTGACAGCAGT
F5B	ATAGCGACAGCAGCAATAGAAGTGACAGTAGTAATAGTAGTGACAGCAGCGATAGCAGTGACAGCAGCAACAGCAGTGACAGCAGTGATAGTAGTGACAGCAGTGACAGCAACGAAAGCAGCAATAGCAGTGACAGCAGTGATAGCAGCAACAGCAGTGATAGTGACAGCAGTGATAGCAGCAACAGCAG**C**GACAGCAGT
HAP1A	ATAGCGACAGCAGCAATAGAAGTGACAGTAGTAATAGTAGTGACAGCAGCGATAGCAGTGACAGCAGCAACAGCAGTGACAGCAGTGATAGTAGTGACAGCAGTGACAGCAACGAAAGCAGCAATAGCAGTGACAGCAGTGATAGCAGCAACAGCAGTGATAGTGACAGCAGTGATAGCAGCAACAGCAGTGACAGCAGT
HAP2A	ATAGCGACAGCAGCAATAGAAGTGACAGTAGTAATAGTAGTGACAGCAGCGATAGCAGTGACAGCAGCAACAGCAGTGACAGCAGTGATAG---------CAGTGACAGCAACGAAAGCAGCAATAGCAGTGACAGCAGTGATAGCAGCAACAGCAGTGACAGTGACAGCAGTGATAGCAGCAACAGCAGTGACAGCAGT
HAP3A	ATAGCGACAGCAGCAATAGAAGTGACAGTAGTAATAGTAGTGACAGCAGCGATAGCAGTGACAGCAGCAACAGCAGTGACAGCAGTGATAG---------CAGTGACAGCAACGAAAGCAGCAATAGCAGTGACAGCAGTGATAGCAGCAACAGCAGTGACAGTGACAGCAGTGATAGCAGCAACAGCAGTGACAGCAGT
HAP15A	ATAGCGACAGCAGCAATAGAAGTGACAGTAGTAATAGTAGTGACAGCAGCGATAGCAGTGACAGCAGCAACAGCAGTGACAGCAGTGATAGTAGTGACAGCAGTGACAGCAACGAAAGCAGCAATAGCAGTGACAGCAGTGATAGCAGCAACAGCAGTGATAGTGACAGCAGTGATAGCAGCAACAGCAG**C**GACAGCAGT
HAP17B	ATAGCGACAGCAGCAATAGAAGTGACAGTAGTAATAGTAGTGACAGCAGCGATAGCAGTGACAGCAGCAACAGCAGTGACAGCAGTGATAGTAGTGACAGCAGTGACAGCAACGAAAGCAGCAATAGCAGTGACAGCAGTGATAGCAGCAACAGCAGTGATAGTGACAGCAGTGATAGCAGCAACAGCAG**C**GACAGCAGT
HAP20A	ATAGCGACAGCAGCAATAGAAGTGACAGTAGTAATAGTAGTGACAGCAGCGATAGCAGTGACAGCAGCAACAGCAGTGACAGCAGTGATAGTAGTGACAGCAGTGACAGCAACGAAAGCAGCAATAGCAGTGACAGCAGTGATAGCAGCAACAGCAGTGATAGTGACAGCAGTGATAGCAGCAACAGCAGT---------
HAP20B	ATAGCGACAGCAGCAATAGAAGTGACAGTAGTAATAGTAGTGACAGCAGCGATAGCAGTGACAGCAGCAACAGCAGTGACAGCAGTGATAGTAGTGACAGCAGTGACAGCAACGAAAGCAGCAATAGCAGTGACAGCAGTGATAGCAGCAACAGCAGTGATAGTGACAGCAGTGATAGCAGCAACAGCAGTGACAGCAGT
HAP36A	ATAGCGACAGCAGCAATAGAAGTGACAGTAGTAATAGTAGTGACAGCAGCGATAGCAGTGACAGCAGCAACAGCAGTGACAGCAGTGATAGTAGTGACAGCAGTGACAGCAACGAAAGCAGCAATAGCAGTGACAGCAGTGATAGCAGCAACAGCAGTGATAGTGACAGCAGTGATAGCAGCAACAGCAGT---------
HAP37A	ATAGCGACAGCAGC------------------------------------GATAGCAGTGACAGCAGCAACAGCAGTGACAGCAGTGATAGTAGTGACAGCAGTGACAGCAACGAAAGCAGCAATAGCAGTGACAGCAGTGATAGCAGCAACAGCAGTGATAGTGACAGCAGTGATAGCAGCAACAGCAGTGACAGCAGT
HAP38A	ATAGC**A**ACAGCAGCAATAGAAGTGACAGTAGTAATAGTAGTGACAGCAGCGATAGCAGTGACAGCAGCAACAGCAGTGACAGCAGTGATAGTAGTGACAGCAGTGACAGCAACGAAAGCAGCAATAGCAGTGACAGCAGTGATAGCAGCAACAGCAGTGATAGTGACAGCAGTGATAGCAGCAACAGCAGT---------
SHAP1:	ATAGCGACAGCAGCAATAGAAGTGACAGTAGTAATAGTAGTGACAGCAGCGATAGCAGTGACAGCAGCAACAGCAGTGACAGCAGTGATAGTAGTGACAGCAGTGACAGCAACGAAAGCAGCAATAGCAGTGACAGCAGTGATAGCAGCAACAGCAGTGATAGTGACAGCAGTGATAGCAGCAACAGCAGTGACAGCAGT
SHAP2:	ATAGCGACAGCAGCAATAGAAGTGACAGTAGTAATAGTAGTGACAGCAGCGATAGCAGTGACAGCAGCAACAGCAGTGACAGCAGTGATAG---------CAGTGACAGCAACGAAAGCAGCAATAGCAGTGACAGCAGTGATAGCAGCAACAGCAGTGATAGTGACAGCAGTGATAGCAGCAACAGCAGTGACAGCAGT
SHAP3:	ATAGCGACAGCAGCAATAGAAGTGACAGTAGTAATAGTAGTGACAGCAGCGATAGCAGTGACAGCAGCAACAGCAGTGACAGCAGTGATAGTAGTGACAGCAGTGACAGCAACGAAAGCAGCAATAGCAGTGACAGCAGTGATAGCAGCAACAGCAGTGATAGTGACAGCAGTGATAGCAGCAACAGCAGTGACAGCAGT
SHAP4:	ATAGCGACAGCAGCAATAGAAGTGACAGTAGTAATAGTAGTGACAGCAGCGATAGCAGTGACAGCAGCAACAGCAGTGACAGCAGTGATAG---------CAGTGACAGCAACGAAAGCAGCAATAGCAGTGACAGCAGTGATAGCAGCAACAGCAGTGATAGTGACAGCAGTGATAGCAGCAACAGCAGTGACAGCAGT
SHAP5:	ATAGCGACAGCAGCAATAGAAGTGACAGTAGTAATAGTAGTGACAGCAGCGATAGCAGTGACAGCAGCAACAGCAGTGACAGCAGTGATAGTAGTGACAGCAGTGACAGCAACGAAAGCAGCAATAGCAGTGACAGCAGTGATAGCAGCAACAGCAGTGATAGTGACAGCAGTGATAGCAGCAACAGCAGTGACAGCAGT
SHAP6:	ATAGCGACAGCAGCAATAGAAG**G**GACAGTAGTAATAGTAGTGACAGCAGCGATAGCAGTGACAGCAGCAACAGCAGTGACAGCAGTGATAGTAGTGACAGCAGTGACAGCAACGAAAGCAGCAATAGCAGTGACAGCAGTGATAGCAGCAACAGCAGTGATAGTGACAGCAGTGATAGCAGCAACAGCAGTGACAGCAGT
SHAP6(2)	ATAGCGACAGCAGCAATAGAAGTGACAGTAGTAATAGTAGTGACAGCAGCGATAGCAGTGACAGCAGCAACAGCAGTGACAGCAGTGATAGTAGTGACAGCAGTGACAGCAACGAAAGCAGCAATAGCAGTGACAGCAGTGATAGCAGCAACAGCAGTGATAGTGACAGCAGTGATAGCAGCAACAGCAGTGACAGCAGT
SHAP7	ATAGCGACAGCAGCAATAGAAGTGACAGTAGTAATAGTAGTGACAGCAGCGATAGCAGTGACAGCAGCAACAGCAGTGACAGCAGTGATAG---------CAGTGACAGCAACGAAAGCAGCAATAGCAGTGACAGCAGTGATAGCAGCAACAGCAGTGATAGTGACAGCAGTGATAGCAGCAACAGCAGTGACAGCAGT
SHAP102	ATAGCGACAGCAGCAATAGAAGTGACAGTAGTAATAGTAGTGACAGCAGCGATAGCAGTGACAGCAGCAACAGCAGTGACAGCAGTGATAGTAGTGACAGCAGTGACAGCAACGAAAGCAGCAATAGCAGTGACAGCAGTGATAGCAGCAACAGCAGTGATAGTGACAGCAGTGATAGCAGCAACAGCAGTGACAGCAGT
SHAP130	ATAGCGACAGCAGCAATAGAAGTGACAGTAGTAATAGTAGTGACAGCAGCGATAGCAGTGACAGCAGCAACAGCAGTGACAGCAGTGATAGTAGTGACAGCAGTGACAGCAACGAAAGCAGCAATAGCAGTGACAGCAGTGATAGCAGCAACAGCAGTGATAGTGACAGCAGTGATAGCAGCAACAGCAGTGACAGCAGT
SHAP106	ATAGCGACAGCAGCAATAGAAGTGACAGTAGTAATAGTAGTGACAGCAGCGATAGCAGTGACAGCAGCAACAGCAGTGACAGCAGTGATAGTAGTGACAGCAGTGACAGCAACGAAAGCAGCAATAGCAGTGACAGCAGTGATAGCAGCAACAGCAGTGATAGTGACAGCAGTGATAGCAGCAACAGCAGTGACAGCAGT
SHAP72	ATAGCGACAGCAGCAATAGAAGTGACAGTAGTAATAGTAGTGACAGCAGCGATAGCAGTGACAGCAGCAACAGCAGTGACAGCAGTGATAGTAGTGACAGCAGTGACAGCAACGAAAGCAGCAATAGCAGTGACAGCAGTGATAGCAGCAACAGCAGTGATAGTGACAGCAGTGATAGCAGCAACAGCAGTGACAGCAGT
SHAP110	ATAGCGACAGCAGCAATAGAAGTGACAGTAGTAATAGTAGTGACAGCAGCGATAGCAGTGACAGCAGCAACAGCAGTGACAGCAGTGATAG---------CAGTGACAGCAACGAAAGCAGCAATAGCAGTGACAGCAGTGATAGCAGCAACAGCAGTGATAGTGACAGCAGTGATAGCAGCAACAGCAGTGACAGCAGT
HAP4	AGAAGTGACAGTAGTAATAGTAGTGACAGCAGCGATAGCAGTGACAGCAGCAACAGCAGTGACAGCAGTGATAGTAGTGACAGCAGTGACAGCAACGAAAGCAGCAATAGCAGTGACAGCAGTGATAGCAGCAACAGCAGTGATAGTGACAGCAGTGATAGCAGCAACAGCAG**C**GACAGCAGT
HAP5	AGAAGTGACAGTAGTAATAGTAGTGACAGCAGCGATAGCAGTGACAGCAGCAACAGCAGTGACAGCAGTGATAGTAGTGACAGCAGTGACAGCAACGAAAGCAGCAATAGCAGTGACAGCAGTGATAGCAGCAACAGCAGTGATAGTGACAGCAGTGATAGCAGCAACAGCAGT---------
HAP8	AGAAG**G**GACAGTAGTAATAGTAGTGACAGCAGCGATAGCAGTGACAGCAGCAACAGCAGTGACAGCAGTGATAGTAGTGACAGCAGTGACAGCAACGAAAGCAGCAATAGCAGTGACAGCAGTGATAGCAGCAACAGCAGTGATAGTGACAGCAGTGATAGCAGCAACAGCAGTGACAGCAGT
HAP13	AGAAGTGACAGTAGTAATAGTAGTGACAGCAGCGATAGCAGTGACAGCAGCAACAGCAGTGACAGCAGTGATAGTAGTGACAGCAGTGACAGCAACGAAAGCAGCAATAGCAGTGACAGCAGTGATAGCAGCAACAGCAGTGATAGTGACAGCAGTGATAGCAGCAACAGCAG**C**GACAGCAGT
HAP24	AGAAGTGACAGTAGTAATAGTAGTGACAGCAGCGATAGCAGTGACAGCAGCAACAGCAGTGACAGCAGTGATAG---------CAGTGACAGCAACGAAAGCAGCAATAGCAGTGACAGCAGTGATAGCAGCAACAGCAGTGA**C**AGTGACAGCAGTGATAGCAGCAACAGCAGTGACAGCAGT
HAP25	AGAAGTGACAGTAGTAATAGTAGTGACAGCAGCGATAGCAGTGACAGCAGCAACAGCAGTGACAGCAGTGATAGTAGTGACAGCAGTGACAGCAACGAAAGCAGCAATAGCAGTGACAGCAGTGATAGCAGCAACAGCAGTGATAGTGACAGCAGTGATAGCAGCAACAGCAGTGACAGCAGT
HAP26	AGAAGTGACAGTAGTAATAGTAGTGACAGCAGCGATAGCAGTGACAGCAGCAACAGCAGTGACAGCAGTGATAGTAGTGACAGCAGTGACAGCAACGAAAGCAGCAATAGCAGTGACAGCAGTGATAGCAGCAACAGCAGTGATAGTGACAGCAGTGATAGCAGCAACAGCAGTGACAGCAGT
HAP29	AGAAGTGACAGTAGTAATAGTAGTGACAGCAGCGATAGCAGTGACAGCAGCAACAGCAGTGACAGCAGTGATAGTAGTGACAGCAGTGACAGCAACGAAAGCAGCAATAGCAGTGACAGCAGTGATAGCAGCAACAGCAGTGATAGTGACAGCAGTGATAGCAGCAACAGCAGTGACAGCAGT
HAP32	AGAAGTGACAGTAGTAATAGTAGTGACAGCAGCGATAGCAGTGACAGCAGCAACAGCAGTGACAGCAGTGATAGTAGTGACAGCAGTGACAGCAACGAAAGCAGCAATAGCAGTGACAGCAGTGATAGCAGCAACAGCAGTGATAGTGACAGCAGTGATAGCAGCAACAGCAGT---------
HAP33	AGAAGTGACAGTAGTAATAGTAGTGACAGCAGCGATAGCAGTGACAGCAGCAACAGCAGTGACAGCAGTGATAGTAGTGACAGCAGTGACAGCAACGAAAGCAGCAATAGCAGTGACAGCAGTGATAGCAGCAACAGCAGTGATAGTGACAGCAGTGATAGCAGCAACAGCAGTGACAGCAGT
HAP35	AGAAGTGACAGTAGTAATAGTAGTGACAGCAGCGATAGCAGTGACAGCAGCAACAGCAGTGACAGCAGTGATAGTAGTGACAGCAGTGACAGCAACGAAAGCAGCAATAGCAGTGACAGCAGTGATAGCAGCAACAGCAGTGATAGTGACAGCAGTGATAGCAGCAACAGCAGTGACAGCAGT
MERGED	ATAGCGACAGCAGCAATAGAAGTGACAGTAGTAATAGTAGTGACAGCAGCGATAGCAGTGACAGCAGCAACAGCAGTGACAGCAGTGATAG**TAGTGACAG**CAGTGACAGCAACGAAAGCAGCAATAGCAGTGACAGCAGTGATAGCAGCAACAGCAGTGATAGTGACAGCAGTGATAGCAGCAACAGCAGTGACAGCAGT
TRANSL	D S D/**N** S S N R S/**R** D S S N S S D S S D S S D S S N S S D S S D S **S D S** S D S N E S S N S S D S S D S S N S S D S D S S D S S N S S D S S 915

Notes:

c.2571T is the beginning of the Hap3 to Hap38 sequences.

SNPs: c.2560G>A (p.Asp854Asn); c.2577T>G (p.Ser859Arg); c.2706T>C; c.2736T>C.

ID7: NM_014208.3:c.2569_2604delAATAGAAGTGACAGTAGTAATAGTAGTGACAGCAGC.

ID8: NM_014208.3:c.2645_2646insTAGTGACAG.

ID9: NM_014208.3:c.2737_2745delGACAGCAGT.

Mutations:

14. g.11727delA; c.2593delA; p.(Ser865Valfs*449)[4]
15. g.11800delG; c.2666delG; p.(Ser889Thrfs*425)[5]
16. g.11818delG; c.2684delG; p.(Ser895Metfs*419)[4,6]
17. g.11822delT; c.2688delT; p.(Asp896Glufs*418)[9]

```
          2746                                                                                    ID10                                                                     ID11                   2927
REFSEQ    GATAGCAGCAACAGCAGTGATAGCAGTGAAAGCAGTAATAGTAGTGACAACAGCAATAGCAGTGACAGCAGCAACAGCAGTGACAGCAGTGATAGCAGTGACAGCAGTAATAGTAGTGACAGCAGCAATAGCAGTGACAGCAGCAACAGCAGTGACAGCAG------------------TGATAGCAATAGCAGCGACAG
Fam8a     GATAGCAGCAACAGCAGTGATAGCAGTGAAAGCAGTAATAGTAGTGACAACAGCAATAGCAGTGACAGCAGCAACAGCAGTGACAGCAGTGATAGCAGTGACAGCAGTAATAGTAGTGACAGCAGCAATAGCGGTGACAGCAGCAACAGCAGTGACAGCAG------------------TGATAGCAATAGCAGCGACAG
Fam8b     GATAGCAGCAACAGCAGTGATAGCAGTGAAAGCAGTAATAGTAGTGACAACAGCAATAGCAGTGACAGCAGCAACAGCAGTGACAGCAGTGATAGCAGTGACAGCAGTAATAGTAGTGACAGCAGCAATAGCAGTGACAGCAGCAACAGCAGTGACAGCAG------------------TGATAGCAATAGCAGCGACAG
Fam9a     GATAGCAGCAACAGCAGTGATAGCAGTGAAAGCAGTAATAGTAGTGACAACAGCAATAGCAGTGACAGCAGCAACAGCAGTGACAGCAGTGATAGCAGTGACAGCAGTAATAGTAGTGACAGCAGCAATAGCAGTGACAGCAGCAACAGCAGTGACAGCAG------------------TGATAGCAATAGCAGCGACAG
Fam9b     GATAGCAGCAACAGCAGTGATAGCAGTGAAAGCAGTAATAGTAGTGACAACAGCAATAGCAGTGACAGCAGCAACAGCAGTGACAGCAGTGATAGCAGTGACAGCAGTAATAGTAGTGACAGCAGCAATAGCGGTGACAGCAGCAACAGCAGTGACAGCAG------------------TGATAGCAATAGCAGCGACAG
Fam10a    GATAGCAGCAACAGCAGTGATAGCAGTGAAAGCAGTAATAGTAGTGACAACAGCAATAGCAGTGACAGCAGCAACAGCAGTGACAGCAGTGATAGCAGTGACAGCAGTAATAGTAGTGACAGCAGCAATAGCGGTGACAGCAGCAACAGCAGTGACAGCAG------------------TGATAGCAATAGCAGCGACAG
Fam10b    GATAGCAGCAACAGCAGTGATAGCAGTGAAAGCAGTAATAGTAGTGACAACAGCAATAGCAGTGACAGCAGCAACAGCAGTGACAGCAGTGATAGCAGTGACAGCAGTAATAGTAGTGACAGCAGCAATAGCGGTGACAGCAGCAACAGCAGTGACAGCAG------------------TGATAGCAATAGCAGCGACAG
Fam11a    GATAGCAGCAACAGCAGTGATAGCAGTGAAAGCAGTAATAGTAGTGACAACAGCAATAGCAGTGACAGCAGCAACAGCAGTGACAGCAGTGATAGCAGTGACAGCAGTAATAGTAGTGACAGCAGCAATAGCGGTGACAGCAGCAACAGCAGTGACAGCAG------------------TGATAGCAATAGCAGCGACAG
Fam11b    GATAGCAGCAACAGCAGTGATAGCAGTGAAAGCAGTAATAGTAGTGACAACAGCAATAGCAGTGACAGCAGCAACAGCAGTGACAGCAGTGATAGCAGTGACAGCAGTAATAGTAGTGACAGCAGCAATAGCGGTGACAGCAGCAACAGCAGTGACAGCAG------------------TGATAGCAATAGCAGCGACAG
Fam12a    GATAGCAGCAACAGCAGTGATAGCAGTGAAAGCAGTAATAGTAGTGACAACAGCAATAGCAGTGACAGCAGCAACAGCAGTGACAGCAGTGATAGCAGTGACAGCAGTAATAGTAGTGACAGCAGCAATAGCGGTGACAGCAGCAACAGCAGTGACAGCAG------------------TGATAGCAATAGCAGCGACAG
Fam12b    GATAGCAGCAACAGCAGTGATAGCAGTGAAAGCAGTAATAGTAGTGACAACAGCAATAGCAGTGACAGCAGCAACAGCAGTGACAGCAGTGATAGCAGTGACAGCAGTAATAGTAGTGACAGCAGCAATAGCGGTGACAGCAGCAACAGCAGTGACAGCAG------------------TGATAGCAATAGCAGCGACAG
F1A       GATAGCAGCAACAGCAGTGATAGCAGTGAAAGCAGTAATAGTAGTGACAACAGCAATAGCAGTGACAGCAGCAACAGCAGTGACAGCAGTGATAGCAGTGACAGCAGTAATAGTAGTGACAGCAGCAATAGCGGTGACAGCAGCAACAGCAGTGACAGCAG------------------TGATAGCAATAGCAGCGACAG
F1B       GATAGCAGCAACAGCAGTGATAGCAGTGAAAGCAGTAATAGTAGTGACAACAGCAATAGCAGTGACAGCAGCAACAGCAGTGACAGCAGTGATAGCAGTGACAGCAGTAATAGTAGTGACAGCAGCAATAGCGGTGACAGCAGCAACAGCAGTGACAGCAG------------------TGATAGCAATAGCAGCGACAG
F2A       GATAGCAGCAACAGCAGTGATAGCAGTGAAAGCAGTAATAGTAGTGACAACAGCAATAGCAGTGACAGCAGCAACAGCAGTGACAGCAGTGATAGCAGTGACAGCAGTAATAGTAGTGACAGCAGCAATAGCGGTGACAGCAGCAACAGCAGTGACAGCAG------------------TGATAGCAATAGCAGCGACAG
F2B       GATAGCAGCAACAGCAGTGATAGCAGTGAAAGCAGTAATAGTAGTGACAACAGCAATAGCAGTGACAGCAGCAACAGCAGTGACAGCAGTGATAGCAGTGACAGCAGTAATAGTAGTGACAGCAGCAATAGCAGTGACAGCAGCAACAGCAGTGACAGCAGCAACAGCAGTGACAGCAGTGATAGCAATAGCAGCGACAG
F3A       GATAGCAGCAACAGCAGTGATAGCAGTGAAAGCAGTAATAGTAGTGACAACAGCAATAGCAGTGACAGCAGCAACAGCAGTGACAGCAGTGATAGCAGTGACAGCAGTAATAGTAGTGACAGCAGCAATAGCGGTGACAGCAGCAACAGCAGTGACAGCAG------------------TGATAGCAATAGCAGCGACAG
F3B       GATAGCAGCAACAGCAGTGATAGCAGTGAAAGCAGTAATAGTAGTGACAACAGCAATAGCAGTGACAGCAGCAACAGCAGTGACAGCAGTGATAGCAGTGACAGCAGTAATAGTAGTGACAGCAGCAATAGCAGTGACAGCAGCAACAGCAGTGACAGCAG------------------TGATAGCAATAGCAGCGACAG
F4A       GATAGCAGCAACAGCAGTGATAGCAGTGAAAGCAGTAATAGTAGTGACAACAGCAATAGCAGTGACAGCAGCAACAGCAGTGACAGCAGTGATAGCAGTGACAGCAGTAATAGTAGTGACAGCAGCAATAGCAGTGACAGCAGCAACAGCAGTGACAGCAG------------------TGATAGCAATAGCAGCGACAG
F4B       GATAGCAGCAACAGCAGTGATAGCAGTGAAAGCAGTAATAGTAGTGACAACAGCAATAGCAGTGACAGCAGCAACAGCAGTGACAGCAGTGATAGCAGTGACAGCAGTAATAGTAGTGACAGCAGCAATAGCAGTGACAGCAGCAACAGCAGTGACAGCAG------------------TGATAGCAATAGCAGCGACAG
F5A       GATAGCAGCAACAGCAGTGATAGCAGTGAAAGCAGTAATAGTAGTGACAACAGCAATAGCAGTGACAGCAGCAACAGCAGTGACAGCAGTGATAGCAGTGACAGCAGTAATAGTAGTGACAGCAGCAATAGCAGTGACAGCAGCAACAGCAGTGACAGCAG------------------TGATAGCAATAGCAGCGACAG
F5B       GATAGCAGCAACAGCAGTGATAGCAGTGAAAGCAGTAATAGTAGTGACAACAGCAATAGCAGTGACAGCAGCAACAGCAGTGACAGCAGTGATAGCAGTGACAGCAGTAATAGTAGTGACAGCAGCAATAGCGGTGACAGCAGCAACAGCAGTGACAGCAG------------------TGATAGCAATAGCAGCGACAG
HAP1A     GATAGCAGCAACAGCAGTGATAGCAGTGAAAGCAGTAATAGTAGTGACAACAGCAATAGCAGTGACAGCAGCAACAGCAGTGACAGCAGTGATAGCAGTGACAGCAGTAATAGTAGTGACAGCAGCAATAGCGGTGACAGCAGCAACAGCAGTGACAGCAG------------------TGATAGCAATAGCAGCGACAG
HAP2A     GATAGCAGCAACAGCAGTGATAGCAGTGAAAGCAGTAATAGTAGTGACAACAGCAATAGCAGTGACAGCAGCAACAGCAGTGACAGCAGTGATAGCAGTGACAGCAGTAATAGTAGTGACAGCAGCAATAGCAGTGACAGCAGCAACAGCAGTGACAGCAG------------------TGATAGCAATAGCAGCGACAG
HAP3A     GATAGCAGCAACAGCAGTGATAGCAGTGAAAGCAGTAATAGTAGTGACAACAGCAATAGCAGTGACAGCAGCAACAGCAGTGACAGCAGTGATAGCAGTGACAGCAGTAATAGTAGTGACAGCAGCAATAGCAGTGACAGCAGCAACAGCAGTGACAGCAG------------------TGATAGCAATAGCAGCGACAG
HAP15A    GATAGCAGCAACAGCAGTGATAGCAGTGAAAGCAGTAATAGTAGTGACAACAGCAATAGCAGTGACAGCAGCAACAGCAGTGACAGCAGTGATAGCAGTGACAGCAGTAATAGTAGTGACAGCAGCAATAGCGGTGACAGCAGCAACAGCAGTGACAGCAG------------------TGATAGCAATAGCAGCGACAG
HAP17B    GATAGCAGCAACAGCAGTGATAGCAGTGAAAGCAGTAATAGTAGTGACAACAGCAATAGCAGTGACAGCAGCAACAGCAGTGACAGCAGTGATAGCAGTGACAGCAGTAATAGTAGTGACAGCAGCAATAGCGGTGACAGCAGCAACAGCAGTGACAGCAG------------------TGATAGCAATAGCAGCGACAG
HAP20A    GATAGCAGCAACAGCAGTGATAGCAGTGAAAGCAGTAATAGTAGTGACAACAGCAATAGCAGTGACAGCAGCAACAGCAGTGACAGCAGTGATAGCAGTGACAGCAGTAATAGTAGTGACAGCAGCAATAGCAGTGACAGCAGCAACAGCAGTGACAGCAG------------------TGATAGCAATAGCAGCGACAG
HAP20B    GATAGCAGCAACAGCAGTGATAGCAGTGAAAGCAGTAATAGTAGTGACAACAGCAATAGCAGTGACAGCAGCAACAGCAGTGACAGCAGTGATAGCAGTGACAGCAGTAATAGTAGTGACAGCAGCAATAGCGGTGACAGCAGCAACAGCAGTGACAGCAG------------------TGATAGCAATAGCAGCGACAG
HAP36A    GATAGCAGCAACAGCAGTGATAGCAGTGAAAGCAGTAATAGTAGTGACAACAGCAATAGCAGTGACAGCAGCAACAGCAGTGACAGCAGTGATAGCAGTGACAGCAGTAATAGTAGTGACAGCAGCAATAGCAGTGACAGCAGCAACAGCAGTGACAGCAG------------------TGATAGCAATAGCAGCGACAG
HAP37A    GATAGCAGCAACAGCAGTGATAGCAGTGAAAGCAGTAATAGTAGTGACAACAGCAATAGCAGTGACAGCAGCAACAGCAGTGACAGCAGT---------GACAGCAGTAATAGTAGTGACAGCAGCAATAGCAGTGACAGCAGCAACAGCAGTGACAGCAG------------------TGATAGCAATAGCAGCGACAG
HAP38A    GATAGCAGCAACAGCAGTGATAGCAGTGAAAGCAGTAATAGTAGTGACAACAGCAATAGCAGTGACAGCAGCAACAGCAGTGACAGCAGTGATAGCAGTGACAGCAGTAATAGTAGTGACAGCAGCAATAGCAGTGACAGCAGCAACAGCAGTGACAGCAG------------------TGATAGCAATAGCAGCGACAG
SHAP1:    GATAGCAGCAACAGCAGTGATAGCAGTGAAAGCAGTAATAGTAGTGACAACAGCAATAGCAGTGACAGCAGCAACAGCAGTGACAGCAGTGATAGCAGTGACAGCAGTAATAGTAGTGACAGCAGCAATAGCGGTGACAGCAGCAACAGCAGTGACAGCAG------------------TGATAGCAATAGCAGCGACAG
SHAP2:    GATAGCAGCAACAGCAGTGATAGCAGTGAAAGCAGTAATAGTAGTGACAACAGCAATAGCAGTGACAGCAGCAACAGCAGTGACAGCAGTGATAGCAGTGACAGCAGTAATAGTAGTGACAGCAGCAATAGCAGTGACAGCAGCAACAGCAGTGACAGCAG------------------TGATAGCAATAGCAGCGACAG
SHAP3:    GATAGCAGCAACAGCAGTGATAGCAGTGAAAGCAGTAATAGTAGTGACAACAGCAATAGCAGTGACAGCAGCAACAGCAGTGACAGCAGTGATAGCAGTGACAGCAGTAATAGTAGTGACAGCAGCAATAGCGGTGACAGCAGCAACAGCAGTGACAGCAG------------------TGATAGCAATAGCAGCGACAG
SHAP4:    GATAGCAGCAACAGCAGTGATAGCAGTGAAAGCAGTAATAGTAGTGACAACAGCAATAGCAGTGACAGCAGCAACAGCAGTGACAGCAGTGATAGCAGTGACAGCAGTAATAGTAGTGACAGCAGCAATAGCAGTGACAGCAGCAACAGCAGTGACAGCAG------------------TGATAGCAATAGCAGCGACAG
SHAP5:    GATAGCAGCAACAGCAGTGATAGCAGTGAAAGCAGTAATAGTAGTGACAACAGCAATAGCAGTGACAGCAGCAACAGCAGTGACAGCAGTGATAGCAGTGACAGCAGTAATAGTAGTGACAGCAGCAATAGCGGTGACAGCAGCAACAGCAGTGACAGCAG------------------TGATAGCAATAGCAGCGACAG
SHAP6:    GATAGCAGCAACAGCAGTGATAGCAGTGAAAGCAGTAATAGTAGTGACAACAGCAATAGCAGTGACAGCAGCAACAGCAGTGACAGCAGTGATAGCAGTGACAGCAGTAATAGTAGTGACAGCAGCAATAGCGGTGACAGCAGCAACAGCAGTGACAGCAG------------------TGATAGCAATAGCAGCGACAG
SHAP6(2)  GATAGCAGCAACAGCAGTGATAGCAGTGAAAGCAGTAATAGTAGTGACAACAGCAATAGCAGTGACAGCAGCAACAGCAGTGACAGCAGTGATAGCAGTGACAGCAGTAATAGTAGTGACAGCAGCAATAGCGGTGACAGCAGCAACAGCAGTGACAGCAG------------------TGATAGCAATAGCAGCGACAG
SHAP7     GATAGCAGCAACAGCAGTGATAGCAGTGAAAGCAGTAATAGTAGTGACAACAGCAATAGCAGTGACAGCAGCAACAGCAGTGACAGCAGTGATAGCAGTGACAGCAGTAATAGTAGTGACAGCAGCAATAGCAGTGACAGCAGCAACAGCAGTGACAGCAG------------------TGATAGCAATAGCAGCGACAG
SHAP102   GATAGCAGCAACAGCAGTGATAGCAGTGAAAGCAGTAATAGTAGTGACAACAGCAATAGCAGTGACAGCAGCAACAGCAGTGACAGCAGTGATAGCAGTGACAGCAGTAATAGTAGTGACAGCAGCAATAGCAGTGACAGCAGCAACAGCAGTGACAGCAG------------------TGATAGCAATAGCAGCGACAG
SHAP130   GATAGCAGCAACAGCAGTGATAGCAGTGAAAGCAGTAATAGTAGTGACAACAGCAATAGCAGTGACAGCAGCAACAGCAGTGACAGCAGTGATAGCAGTGACAGCAGTAATAGTAGTGACAGCAGCAATAGCGGTGACAGCAGCAACAGCAGTGACAGCAG------------------TGATAGCAATAGCAGCGACAG
SHAP106   GATAGCAGCAACAGCAGTGATAGCAGTGAAAGCAGTAATAGTAGTGACAACAGCAATAGCAGTGACAGCAGCAACAGCAGTGACAGCAGTGATAGCAGTGACAGCAGTAATAGTAGTGACAGCAGCAATAGCGGTGACAGCAGCAACAGCAGTGACAGCAG------------------TGATAGCAATAGCAGCGACAG
SHAP72    GATAGCAGCAACAGCAGTGATAGCAGTGAAAGCAGTAATAGTAGTGACAACAGCAATAGCAGTGACAGCAGCAACAGCAGTGACAGCAGTGATAGCAGTGACAGCAGTAATAGTAGTGACAGCAGCAATAGCGGTGACAGCAGCAACAGCAGTGACAGCAG------------------TGATAGCAATAGCAGCGACAG
SHAP110   GATAGCAGCAACAGCAGTGATAGCAGTGAAAGCAGTAATAGTAGTGACAACAGCAATAGCAGTGACAGCAGCAACAGCAGTGACAGCAGTGATAGCAGTGACAGCAGTAATAGTAGTGACAGCAGCAATAGCAGTGACAGCAGCAACAGCAGTGACAGCAG------------------TGATAGCAATAGCAGCGACAG
HAP4      GATAGCAGCAACAGCAGTGATAGCAGTGAAAGCAGTAATAGTAGTGACAACAGCAATAGCAGTGACAGCAGCAACAGCAGTGACAGCAGTGATAGCAGTGACAGCAGTAATAGTAGTGACAGCAGCAATAGCAGTGACAGCAGCAACAGCAGTGACAGCAG------------------TGATAGCAATAGCAGCGACAG
HAP5      GATAGCAGCAACAGCAGTGATAGCAGTGAAAGCAGTAATAGTAGTGACAACAGCAATAGCAGTGACAGCAGCAACAGCAGTGACAGCAGTGATAGCAGTGACAGCAGTAATAGTAGTGACAGCAGCAATAGCAGTGACAGCAGCAACAGCAGTGACAGCAG------------------TGATAGCAATAGCAGCGACAG
HAP8      GATAGCAGCAACAGCAGTGATAGCAGTGAAAGCAGTAATAGTAGTGACAACAGCAATAGCAGTGACAGCAGCAACAGCAGTGACAGCAGTGATAGCAGTGACAGCAGTAATAGTAGTGACAGCAGCAATAGCGGTGACAGCAGCAACAGCAGTGACAGCAG------------------TGATAGCAATAGCAGCGACAG
HAP13     GATAGCAGCAACAGCAGTGATAGCAGTGAAAGCAGTAATAGTAGTGACAACAGCAATAGCAGTGACAGCAGCAACAGCAGTGACAGCAGTGATAGCAGTGACAGCAGTAATAGTAGTGACAGCAGCAATAGCGGTGACAGCAGCAACAGCAGTGACAGCAG------------------TGATAGCAATAGCAGCGACAG
HAP24     GATAGCAGCAACAGCAGTGATAGCAGTGAAAGCAGTAATAGTAGTGACAACAGCAATAGCAGTGACAGCAGCAACAGCAGTGACAGCAGTGATAGCAGTGACAGCAGTAATAGTAGTGACAGCAGCAATAGCAGTGACAGCAGCAACAGCAGTGACAGCAG------------------TGATAGCAATAGCAGCGACAG
HAP25     GATAGCAGCAACAGCAGTGATAGCAGTGAAAGCAGTAATAGTAGTGACAACAGCAATAGCAGTGACAGCAGCAACAGCAGTGACAGCAGTGATAGCAGTGACAGCAGTAATAGTAGTGACAGCAGCAATAGCGGTGACAGCAGCAACAGCAGTGACAGCAG------------------TGATAGCAATAGCAGCGACAG
HAP26     GATAGCAGCAACAGCAGTGATAGCAGTGAAAGCAGTAATAGTAGTGACAACAGCAATAGCAGTGACAGCAGCAACAGCAGTGACAGCAGT---------GACAGCAGTAATAGTAGTGACAGCAGCAATAGCAGTGACAGCAGCAACAGCAGTGACAGCAG------------------TGATAGCAATAGCAGCGACAG
HAP29     GATAGCAGCAACAGCAGTGATAGCAGTGAAAGCAGTAATAGTAGTGACAACAGCAATAGCAGTGACAGCAGCAACAGCAGTGACAGCAGTGATAGCAGTGACAGCAGTAATAGTAGTGACAGCAGCAATAGCAGTGACAGCAGCAACAGCAGTGACAGCAG------------------TGATAGCAATAGCAGCGACAG
HAP32     GATAGCAGCAACAGCAGTGATAGCAGTGAAAGCAGTAATAGTAGTGACAACAGCAATAGCAGTGACAGCAGCAACAGCAGTGACAGCAGTGATAGCAGTGACAGCAGTAATAGTAGTGACAGCAGCAATAGCAGTGACAGCAGCAACAGCAGTGACAGCAG------------------TGATAGCAATAGCAGCGACAG
HAP33     GATAGCAGCAACAGCAGTGATAGCAGTGAAAGCAGTAATAGTAGTGACAACAGCAATAGCAGTGACAGCAGCAACAGCAGTGACAGCAGTGATAGCAGTGACAGCAGTAATAGTAGTGACAGCAGCAATAGCGGTGACAGCAGCAACAGCAGTGACAGCAG------------------TGATAGCAATAGCAGCGACAG
HAP35     GATAGCAGCAACAGCAGTGATAGCAGTGAAAGCAGTAATAGTAGTGACAACAGCAATAGCAGTGACAGCAGCAACAGCAGTGACAGCAGTGATAGCAGTGACAGCAGTAATAGTAGTGACAGCAGCAATAGCGGTGACAGCAGCAACAGCAGTGACAGCAG------------------TGATAGCAATAGCAGCGACAG
MERGED    GATAGCAGCAACAGCAGTGATAGCAGTGAAAGCAGTAATAGTAGTGACAACAGCAATAGCAGTGACAGCAGCAACAGCAGTGACAGCAGTGATAGCAGTGACAGCAGTAATAGTAGTGACAGCAGCAATAGCAGTGACAGCAGCAACAGCAGTGACAGCAG------------------TGATAGCAATAGCAGCGACAG
TRANSL     D  S  S  N  S  S  D  S  S  E  S  S  N  S  S  D  N  S  N  S  S  D  S  S  N  S  S  D  S  S  D  S  S  D  S  S  N  S  S  D  S  S  N  S  S/G D  S  S  N  S  S  D  S  S                   D  S  N  S  S  D  S  976
```

Notes:

SNP: c.2878A>G (p.Ser960Gly).

ID10: NM_014208.3:c.2836_2844delGATAGCAGT.

ID11: NM_014208.3:c.2877_2906dup (Same as designation: NM_014208.3:c.2906_2907insCAACAGCAGTGACAGCAG).

Mutations: No disease-causing frameshifts have been observed in this region.

2928	ID12 ID13 ID14 3100
REFSEQ	CAGTGACAGCAGCAACAGCAGCGATAGCAGTGACAGCAGTGATAGCAGTGACAGCAGTGACAGCAGTGATAGCAG---------------------------CAACAGCAGTGATAGCAGTGACAGCAGTGACAGCAGTGATAGCAGTAATAGTAGTGACAGCAGCAATAGCAGTGACAGCAGCAACAGCAGTGACAGCA
Fam8a	CAGTGACAGCAGCAACAGCAGCGATAGCAGTGACAGCAGTGATAGCAGTGACAGCAGTGACAGCAGTGATAGCAG---------------------------CAACAGCAGTGATAGCAGTGACAGCAGTGACAGCAGTGATAGCAGTAATAGTAGTGACAGCAGCAA------------------CAGCAGTGACAGCA
Fam8b	CAGTGACAGCAGCAACAGCAGCGATAGCAGTGACAGCAGTGATAGCAGTGACAGCAGTGACAGCAGTGATAGCAG---------------------------C**G**ACAGCAGTGATAGCAGTGACAGCAGTGACAGCAGTGATAGCAGTAATAGTAGTGACAGCAGCAA------------------CAGCAGTGACAGCA
Fam9a	CAGTGACAGCAGCAACAGCAGCGATAGCAGTGACAGCAGTGATAGCAGTGACAGCAGTGACAGCAGTGATAGCAG---------------------------C**G**ACAGCAGTGATAGCAGTGACAGCAGTGACAGCAGTGATAGCAGTAATAGTAGTGACAGCAGCAA------------------CAGCAGTGACAGCA
Fam9b	CAGTGACAGCAGCAACAGCAGCGATAGCAGTGACAGCAGTGATAGCAGTGACAGCAGTGACAGCAGTGATAGCAG---------------------------CAACAGCAGTGATAGCAGTGACAGCAGTGACAGCAGTGATAGCAGTAATAGTAGTGACAGCAGCAA------------------CAGCAGTGACAGCA
Fam10a	CAGTGACAGCAGCAACAGCAGCGATAGCAGTGACAGCAGTGATAGCAGTGACAGCAGTGACAGCAGTGATAGCAG---------------------------CAACAGCAGTGATAGCAGTGACAGCAGTGACAGCAGTGATAGCAGTAATAGTAGTGACAGCAGCAA------------------CAGCAGTGACAGCA
Fam10b	CAGTGACAGCAGCAACAGCAGCGATAGCAGTGACAGCAGTGATAGCAGTGACAGCAGTGACAGCAGTGATAGCAG---------------------------CAACAGCAGTGATAGCAGTGACAGCAGTGACAGCAGTGATAGCAGTAATAGTAGTGACAGCAGCAA------------------CAGCAGTGACAGCA
Fam11a	CAGTGACAGCAGCAACAGCAGCGATAGCAGTGACAGCAGTGATAGCAGTGACAGCAGTGACAGCAGTGATAGCAG---------------------------CAACAGCAGTGATAGCAGTGACAGCAGTGACAGCAGTGATAGCAGTAATAGTAGTGACAGCAGCAA------------------CAGCAGTGACAGCA
Fam11b	CAGTGACAGCAGCAACAGCAGCGATAGCAGTGACAGCAGTGATAGCAGTGACAGCAGTGACAGCAGTGATAGCAG---------------------------CAACAGCAGTGATAGCAGTGACAGCAGTGACAGCAGTGATAGCAGTAATAGTAGTGACAGCAGCAA------------------CAGCAGTGACAGCA
Fam12a	CAGTGACAGCAGCAACAGCAGCGATAGCAGTGACAGCAGTGATAGCAGTGACAGCAGTGACAGCAGTGATAGCAG---------------------------CAACAGCAGTGATAGCAGTGACAGCAGTGACAGCAGTGATAGCAGTAATAGTAGTGACAGCAGCAA-----------------ACAGCAGTGACAGCA
Fam12b	CAGTGACAGCAGCAACAGCAGCGATAGCAGTGACAGCAGTGATAGCAGTGACAGCAGTGACAGCAGTGATAGCAG---------------------------CAACAGCAGTGATAGCAGTGACAGCAGTGACAGCAGTGATAGCAGTAATAGTAGTGACAGCAGCAA------------------CAGCAGTGACAGCA
F1A	CAGTGACAGCAGCAACAGCAGCGATAGCAGTGACAGCAGTGATAGCAGTGACAGCAGTGACAGCAGTGATAGCAG---------------------------CAACAGCAGTGATAGCAGTGACAGCAGTGACAGCAGTGATAGCAGTAATAGTAGTGACAGCAGCAA------------------CAGCAGTGACAGCA
F1B	CAGTGACAGCAGCAACAGCAGCGATAGCAGTGACAGCAGTGATAGCAGTGACAGCAGTGACAGCAGTGATAGCAG---------------------------CAACAGCAGTGATAGCAGTGACAGCAGTGACAGCAGTGATAGCAGTAATAGTAGTGACAGCAGCAA------------------CAGCAGTGACAGCA
F2A	CAGTGACAGCAGCAACAGCAGCGATAGCAGTGACAGCAGTGATAGCAGTGACAGCAGTGACAGCAGTGATAGCAG---------------------------CAACAGCAGTGATAGCAGTGACAGCAGTGACAGCAGTGATAGCAGTAATAGTAGTGACAGCAGCAA------------------CAGCAGTGACAGCA
F2B	CAGTGACAGCAGCAACAGCAGCGATAGCAGTGACAGCAGTGATAGCAGTGACAGCAGTGACAGCAGTGATAGCAG---------------------------CAACAGCAGTGATAGCAGTGACAGCAGTGACAGCAGTGATAGCAGTAATAGTAGTGACAGCAGCAATAGCAGTGACAGCAGCAACAGCAGTGACAGCA
F3A	CAGTGACAGCAGCAACAGCAGCGATAGCAGTGACAGCAGTGATAGCAGTGACAGCAGTGACAGCAGTGATAGCAG---------------------------CAACAGCAGTGATAGCAGTGACAGCAGTGACAGCAGTGATAGCAGTAATAGTAGTGACAGCAGCAA------------------CAGCAGTGACAGCA
F3B	CAGTGACAGCAGCAACAGCAGCGATAGCAGTGACAGCAGTGATAGCAGTGACAGCAGTGACAGCAGTGATAGCAG---------------------------C**G**ACAGCAGTGATAGCAGTGACAGCAGTGACAGCAGTGATAGCAGTAATAGTAGTGACAGCAGCAA------------------CAGCAGTGACAGCA
F4A	CAGTGACAGCAGCAACAGCAGCGATAGCAGTGACAGCAGTGATAGCAGTGACAGCAGTGACAGCAGTGATAGCAGTGACAGCAGTGACAGCAGTGATAGCAGCAACAGCAGTGATAGCAGTGACAGCAGTGACAGCAGTGATAGCAGTAATAGTAGTGACAGCAGCAATAGCAGTGACAGCAGCAACAGCAGTGACAGCA
F4B	CAGTGACAGCAGCAACAGCAGCGATAGCAGTGACAGCAGTGATAGCAGTGACAGCAGTGACAGCAGTGATAGCAG---------------------------CAACAGCAGTGATAGCAGTGACAGCAGTGACAGCAGTGATAGCAGTAATAGTAGTGACAGCAGCAATAGCAGTGACAGCAGCAACAGCAGTGACAGCA
F5A	CAGTGACAGCAGCAACAGCAGCGATAGCAGTGACAGCAGTGATAGCAGTGACAGCAGTGACAGCAGTGATAGCAG---------------------------C**G**ACAGCAGTGATAGCAGTGACAGCAGTGACAGCAGTGATAGCAGTAATAGTAGTGACAGCAGCAA------------------CAGCAGTGACAGCA
F5B	CAGTGACAGCAGCAACAGCAGCGATAGCAGTGACAGCAGTGATAGCAGTGACAGCAGTGACAGCAGTGATAGCAG---------------------------CAACAGCAGTGATAGCAGTGACAGCAGTGACAGCAGTGATAGCAGTAATAGTAGTGACAGCAGCAA------------------CAGCAGTGACAGCA
HAP1A	CAGTGACAGCAGCAACAGCAGCGATAGCAGTGACAGCAGTGATAGCAGTGACAGCAGTGACAGCAGTGATAGCAG---------------------------CAACAGCAGTGATAGCAGTGACAGCAGTGACAGCAGTGATAGCAGTAATAGTAGTGACAGCAGCAA------------------CAGCAGTGACAGCA
HAP2A	CAGTGACAGCAGCAACAGCAGCGATAGCAGTGACAGCAGTGATAGCAGTGACAGCAGTGACAGCAGTGATAGCAG---------------------------C**G**ACAGCAGTGATAGCAGTGACAGCAGTGACAGCAGTGATAGCAGTAATAGTAGTGACAGCAGCAA------------------CAGCAGTGACAGCA
HAP3A	CAGTGACAGCAGCAACAGCAGCGATAGCAGTGACAGCAGTGATAGCAGTGACAGCAGTGACAGCAGTGATAGCAG---------------------------CAACAGCAGTGATAGCAGTGACAGCAGTGACAGCAGTGATAGCAGTAATAGTAGTGACAGCAGCAATAGCAGTGACAGCAGCAACAGCAGTGACAGCA
HAP15A	CAGTGACAGCAGCAACAGCAGCGATAGCAGTGACAGCAGTGATAGCAGTGACAGCAGTGACAGCAGTGATAGCAG---------------------------CAACAGCAGTGATAGCAGTGACAGCAGTGACAGCAGTGATAGCAGTAATAGTAGTGACAGCAGCAA------------------CAGCAGTGACAGCA
HAP17B	CAGTGACAGCAGCAACAGCAGCGATAGCAGTGACAGCAGTGATAGCAGTGACAGCAGTGACAGCAGTGATAGCAG---------------------------CAACAGCAGTGATAGCAGTGACAGCAGTGACAGCAGTGATAGCAGTAATAGTAGTGACAGCAGCAA------------------CAGCAGTGACAGCA
HAP20A	CAGTGACAGCAGCAACAGCAGCGATAGCAGTGACAGCAGTGATAGCAGTGACAGCAGTGACAGCAGTGATAGCAG---------------------------C**G**ACAGCAGTGATAGCAGTGACAGCAGTGACAGCAGTGATAGCAGTAATAGTAGTGACAGCAGCAA------------------CAGCAGTGACAGCA
HAP20B	CAGTGACAGCAGCAACAGCAGCGATAGCAGTGACAGCAGTGATAGCAGTGACAGCAGTGACAGCAGTGATAGCAG---------------------------CAACAGCAGTGATAGCAGTGACAGCAGTGACAGCAGTGATAGCAGTAATAGTAGTGACAGCAGCAA------------------CAGCAGTGACAGCA
HAP36A	CAGTGACAGCAGCAACAGCAGCGATAGCAGTGACAGCAGTGATAGCAGTGACAGCAGTGACAGCAGTGATAGCAG---------------------------CAACAGCAGTGATAGCAGTGACAGCAGTGACAGCAGTGATAGCAGTAATAGTAGTGACAGCAGCAA**C**AGCAGTGACAGCAGCAACAGCAGTGACAGCA
HAP37A	CAGTGACAGCAGCAACAGCAGCGATAGCAG------------------TGACAGCAGTGACAGCAGTGATAGCAG---------------------------CAACAGCAGTGATAGCAGTGACAGCAGTGACAGCAGTGATAGCAGTAATAGTAGTGACAGCAGCAA------------------CAGCAGTGACAGCA
HAP38A	CAGTGACAGCAGCAACAGCAGCGATAGCAGTGACAGCAGTGATAGCAGTGACAGCAGTGACAGCAGTGATAGCAG---------------------------CAACAGCAGTGATAGCAGTGACAGCAGTGACAGCAGTGATAGCAGTAATAGTAGTGACAGCAGCAATAGCAGTGACAGCAGCAACAGCAGTGACAGCA
SHAP1:	CAGTGACAGCAGCAACAGCAGCGATAGCAGTGACAGCAGTGATAGCAGTGACAGCAGTGACAGCAGTGATAGCAG---------------------------CAACAGCAGTGATAGCAGTGACAGCAGTGACAGCAGTGATAGCAGTAATAGTAGTGACAGCAGCAA------------------CAGCAGTGACAGCA
SHAP2:	CAGTGACAGCAGCAACAGCAGCGATAGCAGTGACAGCAGTGATAGCAGTGACAGCAGTGACAGCAGTGATAGCAG---------------------------CAACAGCAGTGATAGCAGTGACAGCAGTGACAGCAGTGATAGCAGTAATAGTAGTGACAGCAGCAATAGCAGTGACAGCAGCAACAGCAGTGACAGCA
SHAP3:	CAGTGACAGCAGCAACAGCAGCGATAGCAGTGACAGCAGTGATAGCAGTGACAGCAGTGACAGCAGTGATAGCAG---------------------------C**G**ACAGCAGTGATAGCAGTGACAGCAGTGACAGCAGTGATAGCAGTAATAGTAGTGACAGCAGCAA------------------CAGCAGTGACAGCA
SHAP4:	CAGTGACAGCAGCAACAGCAGCGATAGCAGTGACAGCAGTGATAGCAGTGACAGCAGTGACAGCAGTGATAGCAG---------------------------C**G**ACAGCAGTGATAGCAGTGACAGCAGTGACAGCAGTGATAGCAGTAATAGTAGTGACAGCAGCAA------------------CAGCAGTGACAGCA
SHAP5:	CAGTGACAGCAGCAACAGCAGCGATAGCAGTGACAGCAGTGATAGCAGTGACAGCAGTGACAGCAGTGATAGCAG---------------------------C**G**ACAGCAGTGATAGCAGTGACAGCAGTGACAGCAGTGATAGCAGTAATAGTAGTGACAGCAGCAA------------------CAGCAGTGACAGCA
SHAP6:	CAGTGACAGCAGCAACAGCAGCGATAGCAGTGACAGCAGTGATAGCAGTGACAGCAGTGACAGCAGTGATAGCAG---------------------------CAACAGCAGTGATAGCAGTGACAGCAGTGACAGCAGTGATAGCAGTAATAGTAGTGACAGCAGCAA------------------CAGCAGTGACAGCA
SHAP6(2)	CAGTGACAGCAGCAACAGCAGCGATAGCAGTGACAGCAGTGATAGCAGTGACAGCAGTGACAGCAGTGATAGCAG---------------------------CAACAGCAGTGATAGCAGTGACAGCAGTGACAGCAGTGATAGCAGTAATAGTAGTGACAGCAGCAA------------------CAGCAGTGACAGCA
SHAP7	CAGTGACAGCAGCAACAGCAGCGATAGCAGTGACAGCAGTGATAGCAGTGACAGCAGTGACAGCAGTGATAGCAG---------------------------CAACAGCAGTGATAGCAGTGACAGCAGTGACAGCAGTGATAGCAGTAATAGTAGTGACAGCAGCAA------------------CAGCAGTGACAGCA
SHAP102	CAGTGACAGCAGCAACAGCAGCGATAGCAGTGACAGCAGTGATAGCAGTGACAGCAGTGACAGCAGTGATAGCAG---------------------------C**G**ACAGCAGTGATAGCAGTGACAGCAGTGACAGCAGTGATAGCAGTAATAGTAGTGACAGCAGCAA------------------CAGCAGTGACAGCA
SHAP130	CAGTGACAGCAGCAACAGCAGCGATAGCAGTGACAGCAGTGATAGCAGTGACAGCAGTGACAGCAGTGATAGCAG---------------------------CAACAGCAGTGATAGCAGTGACAGCAGTGACAGCAGTGATAGCAGTAATAGTAGTGACAGCAGCAA------------------CAGCAGTGACAGCA
SHAP106	CAGTGACAGCAGCAACAGCAGCGATAGCAGTGACAGCAGTGATAGCAGTGACAGCAGTGACAGCAGTGATAGCAG---------------------------CAACAGCAGTGATAGCAGTGACAGCAGTGACAGCAGTGATAGCAGTAATAGTAGTGACAGCAGCAA------------------CAGCAGTGACAGCA
SHAP72	CAGTGACAGCAGCAACAGCAGCGATAGCAGTGACAGCAGTGATAGCAGTGACAGCAGTGACAGCAGTGATAGCAG---------------------------CAACAGCAGTGATAGCAGTGACAGCAGTGACAGCAGTGATAGCAGTAATAGTAGTGACAGCAGCAA------------------CAGCAGTGACAGCA
SHAP110	CAGTGACAGCAGCAACAGCAGCGATAGCAGTGACAGCAGTGATAGCAGTGACAGCAGTGACAGCAGTGATAGCAG---------------------------CAACAGCAGTGATAGCAGTGACAGCAGTGACAGCAGTGATAGCAGTAATAGTAGTGACAGCAGCAA------------------CAGCAGTGACAGCA
HAP4	CAGTGACAGCAGCAACAGCAGCGATAGCAGTGACAGCAGTGATAGCAGTGACAGCAGTGACAGCAGTGATAGCAG---------------------------C**G**ACAGCAGTGATAGCAGTGACAGCAGTGACAGCAGTGATAGCAGTAATAGTAGTGACAGCAGCAA------------------CAGCAGTGACAGCA
HAP5	CAGTGACAGCAGCAACAGCAGCGATAGCAGTGACAGCAGTGATAGCAGTGACAGCAGTGACAGCAGTGATAGCAG---------------------------C**G**ACAGCAGTGATAGCAGTGACAGCAGTGACAGCAGTGATAGCAGTAATAGTAGTGACAGCAGCAA------------------CAGCAGTGACAGCA
HAP8	CAGTGACAGCAGCAACAGCAGCGATAGCAGTGACAGCAGTGATAGCAGTGACAGCAGTGACAGCAGTGATAGCAG---------------------------CAACAGCAGTGATAGCAGTGACAGCAGTGACAGCAGTGATAGCAGTAATAGTAGTGACAGCAGCAA------------------CAGCAGTGACAGCA
HAP13	CAGTGACAGCAGCAACAGCAGCGATAGCAGTGACAGCAGTGATAGCAGTGACAGCAGTGACAGCAGTGATAGCAG---------------------------CAACAGCAGTGATAGCAGTGACAGCAGTGACAGCAGTGATAGCAGTAATAGTAGTGACAGCAGCAA------------------CAGCAGTGACAGCA
HAP24	CAGTGACAGCAGCAACAGCAGCGATAGCAGTGACAGCAGTGATAGCAGTGACAGCAGTGACAGCAGTGATAGCAG---------------------------CAACAGCAGTGATAGCAGTGACAGCAGTGACAGCAGTGATAGCAGTAATAGTAGTGACAGCAGCAATAGCAGTGACAGCAGCAACAGCAGTGACAGCA
HAP25	CAGTGACAGCAGCAACAGCAGCGATAGCAGTGACAGCAG**CA**ATAGCAGTGACAGCAGTGACAGCAGTGATAGCAG---------------------------CAACAGCAGTGATAGCAGTGACAGCAGTGACAGCAGTGATAGCAGTAATAGTAGTGACAGCAGCAATAGCAGTGACAGCAGCA**G**CAGCAGTGACAGCA
HAP26	CAGTGACAGCAGCAACAGCAGCGATAGCAG------------------TGACAGCAGTGACAGCAGTGATAGCAG---------------------------CAACAGCAGTGATAGCAGTGACAGCAGTGACAGCAGTGATAGCAGTAATAGTAGTGACAGCAGCAA------------------CAGCAGTGACAGCA
HAP29	CAGTGACAGCAGCAACAGCAGCGATAGCAG------------------TGACAGCAGTGACAGCAGTGATAGCAG---------------------------CAACAGCAGTGATAGCAGTGACAGCAGTGACAGCAGTGATAGCAGTAATAGTAGTGACAGCAGCAA------------------CAGCAGTGACAGCA
HAP32	CAGTGACAGCAGCAACAGCAGCGATAGCAGTGACAGCAGTGATAGCAGTGACAGCAGTGACAGCAGTGATAGCAG---------------------------CAACAGCAGTGATAGCAGTGACAGCAGTGACAGCAGTGATAGCAGTAATAGTAGTGACAGCAGCAATAGCAGTGACAGCAGCAACAGCAGTGACAGCA
HAP33	CAGTGACAGCAGCAACAGCAGCGATAGCAGTGACAGCAGTGATAGCAGTGACAGCAGTGACAGCAGTGATAGCAG---------------------------CAACAGCAGTGATAGCAGTGACAGCAGTGACAGCAGTGATAGCAGTAATAGTAGTGACAGCAGCAA------------------CAGCAGTGACAGCA
HAP35	CAGTGACAGCAGCAACAGCAGCGATAGCAGTGACAGCAGTGATAGCAGTGACAGCAGTGACAGCAGTGATAGCAG---------------------------CAACAGCAGTGATAGCAGTGACAGCAGTGACAGCAGTGATAGCAGTAATAGTAGTGACAGCAGCAA------------------CAGCAGTGACAGCA
MERGED	CAGTGACAGCAGCAACAGCAGCGATAGCAGTGACAGCAGTGATAGCAGTGACAGCAGTGACAGCAGTGATAGCAG**TGACAGCAGTGACAGCAGTGATAGCAG**CAACAGCAGTGATAGCAGTGACAGCAGTGACAGCAGTGATAGCAGTAATAGTAGTGACAGCAGCAATAGCAGTGACAGCAGCAACAGCAGTGACAGCA
TRANSL	S D S S N S S D S S D S S D/**N** S S D S S D S S D S S **D S S D S S D S S** N/**D** S S D S S D S S D S S D S S N S S D S S N S S D S S N/**S** S S D S

Notes:

SNPs: c.2967T>C; c.2968G>A (p.Asp990Asn); c.3004A>G (p.Asn1002Asp); c.3069T>C; c.3085A>G (p.Asn1029Asp); c.3086A>G (p.Asn1029Ser); c.3087C>T.

ID12: NM_014208.3:c.2958_2975delTGACAGCAGTGATAGCAG.

ID13: NM_014208.3:c.2976_3002dup (Same as designation: NM_014208.3:c.3002_3003insTGACAGCAGTGACAGCAGTGATAGCAG).
ID14: NM_014208.3:c.3069_3086delTAGCAGTGACAGCAGCAA.

Mutations: No disease-causing frameshifts have been observed in this region.

```
      3101                                3135       ID15             ID16                                          ID17                                            ID18                                      ID19 3265  ID20
REFSEQ    GCGATAGCAGTGACAGCAGCGATAGCAGTGACAGCAGCGATAGCAGTGACAGCAGTGACAGCAGCAATAGCAGTGACAGCAGTGACAGCAGCGACA---------------------------GCAGTGATAGCAGTGACAGCAGTGACAGCAGCGACAGCAGTGATAGCAGTGAAAGCAGTGATAGCAGTG[------]
Fam8a     GCGATAGCAGTGACAGCAGCGATAGCAGTGACAGCAGCGATAGCAGTGACAGCAGTGACAGCAGCAATAGCAGTGACAGCAGTGACAGCAGCGACA---------------------------GCAGTGATAGCAGTGACAGCAGTGACAGCAGCGACAGCAGTGATAGCAGTGACAGCAGTGATAGCAGCG[------]
Fam8b     GCGATAGCAGTGACAGCAGCGATAGCAGTGACAGCAGCGATAGCAGTGACAGCAGTGACAGCAGCAATAGCAGTGACAGCAGTGACAGCAGCGACA---------------------------GCAGTGATAGCAGTGACAGCAGTGACAGCAGCGACAGCAGTGATAGCAGTGAAAGCAGTGATAGCAGCG[------]
Fam9a     GCGATAGCAGTGACAGCAGCGATAGCAGTGACAGCAGCGATAGCAGTGACAGCAGTGACAGCAGCAATAGCAGTGACAGCAGTGACAGCAGCGACA---------------------------GCAGTGATAGCAGTGACAGCAGTGACAGCAGCGACAGCAGTGATAGCAGTGAAAGCAGTGATAGCAGCG[------]
Fam9b     GCGATAGCAGTGACAGCAGCGATAGCAGTGACAGCAGCGATAGCAGTGACAGCAGTGACAGCAGCAATAGCAGTGACAGCAGTGACAGCAGCGACA---------------------------GCAGTGATAGCAGTGACAGCAGTGACAGCAGCGACAGCAGTGATAGCAGTGAAAGCAGTGATAGCAGCG[------]
Fam10a    GCGATAGCAGTGACAGCAGCGATAGCAGTGACAG-------------------AGTGACAGCAGCAATAGCAGTGACAGCAGTGACAGCAGCGACA---------------------------GCAGTGATAGCAGTGACAGCAGTGGCAGCAGCGACAGCAGTGATAGCAGTGACAGCAGTGATAGCAGCG[------]
Fam10b    GCGATAGCAGTGACAGCAGCGATAGCAGTGACAGCAGCGATAGCAGTGACAGCAGTGACAGCAGCAATAGCAGTGACAGCAGTGACAGCAGCGACA---------------------------GCAGTGATAGCAGTGACAGCAGTGACAGCAGCGACAGCAGTGATAGCAGTGACAGCAGTGATAGCAGCG[------]
Fam11a    GCGATAGCAGTGACAGCAGCGATAGCAGTGACAGCAGCGATAGCAGTGACAGCAGTGACAGCAGCAATAGCAGTGACAGCAGTGACAGCAGCGACA---------------------------GCAGTGATAGCAGTGACAGCAGTGACAGCAGCGACAGCAGTGATAGCAGTGACAGCAGTGATAGCAGCG[------]
Fam11b    GCGATAGCAGTGACAGCAGCGATAGCAGTGACAGCAGCGATAGCAGTGACAGCAGTGACAGCAGCAATAGCAGTGACAGCAGTGACAGCAGCGACA---------------------------GCAGTGATAGCAGTGACAGCAGTGACAGCAGCGACAGCAGTGATAGCAGTGACAGCAGTGATAGCAGCG[------]
Fam12a    GCGATAGCAGTGACAGCAGCGATAGCAGTGACAGC------------------AGTGACAGCAGCAATAGCAGTGACAGCAGTGACAGCAGCGACA---------------------------GCAGTGATAGCAGTGACAGCAGTGGCAGCAGCGACAGCAGTGATAGCAGTGACAGCAGTGATAGCAGCG[------]
Fam12b    GCGATAGCAGTGACAGCAGCGATAGCAGTGACAGCAGCGATAGCAGTGACAGCAGTGACAGCAGCAATAGCAGTGACAGCAGTGACAGCAGCGACA---------------------------GCAGTGATAGCAGTGACAGCAGTGACAGCAGCGACAGCAGTGATAGCAGTGACAGCAGTGATAGCAGCG[------]
F1A       GCGATAGCAGTGACAGCAGCGATAGCAGTGACAG-------------------AGTGACAGCAGCAATAGCAGTGACAGCAGTGACAGCAGCGACA---------------------------GCAGTGATAGCAGTGACAGCAGTGGCAGCAGCGACAGCAGTGATAGCAGTGACAGCAGTGATAGCAGCG[------]
F1B       GCGATAGCAGTGACAGCAGCGATAGCAGTGACAGC------------------AGTGACAGCAGCAATAGCAGTGACAGCAGTGACAGCAGCGACA---------------------------GCAGTGATAGCAGTGACAGCAGTGGCAGCAGCGACAGCAGTGATAGCAGTGACAGCAGTGATAGCAGCG[------]
F2A       GCGATAGCAGTGACAGCAGCGATAGCAGTGACAGCAGCGATAGCAGTGACAGCAGTGACAGCAGCAATAGCAGTGACAGCAGTGACAGCAGCGACA---------------------------GCAGTGATAGCAGTGACAGCAGTGACAGCAGCGACAGCAGTGATAGCAGTGACAGCAGTGATAGCAGCG[------]
F2B       GCGATAGCAGTGACAGCAGCGATAGCAGTGACAGCAGCGATAGCAGTGACAGCAGTGACAGCAGCAATAGCAGTGACAGCAGTGACAGCAGCGACA---------------------------GCAGTGATAGCAGTGACAGCAGTGACAGCAGCGACAGCAGTGATAGCAGTGAAAGCAGTGATAGCAGTG[------]
F3A       GCGATAGCAGTGACAGCAGCGATAGCAGTGACAGCAGCGATAGCAGTGACAGCAGTGACAGCAGCAATAGCAGTGACAGCAGTGACAGCAGCGACA---------------------------GCAGTGATAGCAGTGACAGCAGTGACAGCAGCGACAGCAGTGATAGCAGTGACAGCAGTGATAGCAGCG[------]
F3B       GCGATAGCAGTGACAGCAGCGATAGCAGTGACAGCAGCGATAGCAGTGACAGCAGTGACAGCAGCAATAGCAGTGACAGCAGTGACAGCAGCGACA---------------------------GCAGTGATAGCAGTGACAGCAGTGACAGCAGCGACAGCAGTGATAGCAGTGAAAGCAGTGATAGCAGCG[------]
F4A       GCGATAGCAGTGACAGCAGCGATAGCAGTGACAGC------------------AGCGACAGCAGCAATAGCAGTGACAGCAGTGACAGCAGCGACA---------------------------GCAGTGATAGCAGTGACAGCAG---------CGACAGCAGTGATAGCAGTGACAGCAGTG---------[------]
F4B       GCGATAGCAGTGACAGCAGCGATAGCAGTGACAGCAGCGATAGCAGTGACAGCAGTGACAGCAGCAATAGCAGTGACAGCAGTGACAGCAGCGACA---------------------------GCAGTGATAGCAGTGACAGCAGTGACAGCAGCGACAGCAGTGATAGCAGTGAAAGCAGTGATAGCAGTG[------]
F5A       GCGATAGCAGTGACAGCAGCGATAGCAGTGACAGCAGCGATAGCAGTGACAGCAGTGACAGCAGCAATAGCAGTGACAGCAGTGACAGCAGCGACA---------------------------GCAGTGATAGCAGTGACAGCAGTGACAGCAGCGACAGCAGTGATAGCAGTGAAAGCAGTGATAGCAGCG[------]
F5B       GCGATAGCAGTGACAGCAGCGATAGCAGTGACAGCAGCGATAGCAGTGACAGCAGTGACAGCAGCAATAGCAGTGACAGCAGTGACAGCAGCGACA---------------------------GCAGTGATAGCAGTGACAGCAGTGACAGCAGCGACAGCAGTGATAGCAGTGACAGCAGTGATAGCAGCG[------]
HAP1A     GCGATAGCAGTGACAGCAGCGATAGCAGTGACAGC------------------AGTGACAGCAGCAATAGCAGTGACAGCAGTGACAGCAGCGACA---------------------------GCAGTGATAGCAGTGACAGCAGTGGCAGCAGCGACAGCAGTGATAGCAGTGACAGCAGTGATAGCAGCG[------]
HAP2A     GCGATAGCAGTGACAGCAGCGATAGCAGTGACAGCAGCGATAGCAGTGACAGCAGTGACAGCAGCAATAGCAGTGACAGCAGTGACAGCAGCGACA---------------------------GCAGTGATAGCAGTGACAGCAGTGACAGCAGCGACAGCAGTGATAGCAGTGAAAGCAGTGATAGCAGCG[------]
HAP3A     GCGATAGCAGTGACAGCAGCGATAGCAGTGACAGCAGCGATAGCAGTGACAGCAGTGACAGCAGCAATAGCAGTGACAGCAGTGACAGCAGCGACA---------------------------GCAGTGATAGCAGTGACAGCAGTGACAGCAGCGACAGCAGTGATAGCAGTGAAAGCAGTGATAGCAGTG[------]
HAP15A    GCGATAGCAGTGACAGCAGCGATAGCAGTGACAGCAGCGATAGCAGTGACAGCAGTGACAGCAGCAATAGCAGTGACAGCAGTGACAGCAGCGACA---------------------------GCAGTGATAGCAGTGACAGCAGTGACAGCAGCGACAGCAGTGATAGCAGTGACAGCAGTGATAGCAGCG[------]
HAP17B    GCGATAGCAGTGACAGCAGCGATAGCAGTGACAGCAGCGATAGCAGTGACAGCAGTGACAGCAGCAATAGCAGTGACAGCAGTGACAGCAGCGACA---------------------------GCAGTGATAGCAGTGACAGCAGTGACAGCAGCGACAGCAGTGATAGCAGTGACAGCAGTGATAGCAGCG[------]
HAP20A    GCGATAGCAGTGACAGCAGCGATAGCAGTGACAGCAGCGATAGCAGTGACAGCAGTGACAGCAGCAATAGCAGTGACAGCAGTGACAGCAGCGACA---------------------------GCAGTGATAGCAGTGACAGCAGTGACAGCAGCGACAGCAGTGATAGCAGTGAAAGCAGTGATAGCAGCG[------]
HAP20B    GCGATAGCAGTGACAGCAGCGATAGCAGTGACAGCAGCGATAGCAGTGACAGCAGTGACAGCAGCAATAGCAGTGACAGCAGTGACAGCAGCGACA---------------------------GCAGTGATAGCAGTGACAGCAGTGACAGCAGCGACAGCAGTGATAGCAGTGACAGCAGTGATAGCAGCG[------]
HAP36A    GCGATAGCAGTGACAGCAGCGATAGCAGTGACAGCAGCGATAGCAGTGACAGCAGTGACAGCAGCAATAGCAGTGACAGCAGTGACAGCAGCGACA---------------------------GCAGTGATAGCAGTGACAGCAGTGACAGCAGCGACAGCAGTGATAGCAGTGAAAGCAGTGATAGCAGCG[------]
HAP37A    GCGATAGCAGTGACAGCAGCGATAGCAGTGACAGC------------------AGTGACAGCAGCAATAGCAGTGACAGCAGTGACAGCAGCGACA---------------------------GCAGTGATAGCAGTGACAGCAGTGACAGCAGCGACAGCAGTGATAGCAGTGACAGCAGTGATAGCAGCG[------]
HAP38A    GCGATAGCAGTGACAGCAGCGATAGCAGTGACAGC------------------AGTGACAGCAGCAATAGCAGTGACAGCAGTGACAGCAGCGACA---------------------------GCAGTGATAGCAGTGACAGCAGTGACAGCAGCGACAGCAGTGATAGCAGTGAAAGCAGTGATAGCAGCG[------]
SHAP1:    GCGATAGCAGTGACAGCAGCGATAGCAGTGACAGCAGCGATAGCAGTGACAGCAGTGACAGCAGCAATAGCAGTGACAGCAGTGACAGCAGCGACA---------------------------GCAGTGATAGCAGTGACAGCAGTGACAGCAGCGACAGCAGTGATAGCAGTGACAGCAGTGATAGCAGCG[------]
SHAP2:    GCGATAGCAGTGACAGCAGCGATAGCAGTGACAGCAGCGATAGCAGTGACAGCAGTGACAGCAGCAATAGCAGTGACAGCAGTGACAGCAGCGACA---------------------------GCAGTGATAGCAGTGACAGCAGTGACAGCAGCGACAGCAGTGATAGCAGTGAAAGCAGTGATAGCAGTG[------]
SHAP3:    GCGATAGCAGTGACAGCAGCGATAGCAGTGACAGCAGCGATAGCAGTGACAGCAGTGACAGCAGCAATAGCAGTGACAGCAGTGACAGCAGCGACA---------------------------GCAGTGATAGCAGTGACAGCAGTGACAGCAGCGACAGCAGTGATAGCAGTGACAGCAGTGATAGCAGCG[------]
SHAP4:    GCGATAGCAGTGACAGCAGCGATAGCAGTGACAGCAGCGATAGCAGTGACAGCAGTGACAGCAGCAATAGCAGTGACAGCAGTGACAGCAGCGACA---------------------------GCAGTGATAGCAGTGACAGCAGTGACAGCAGCGACAGCAGTGATAGCAGTGAAAGCAGTGATAGCAGCG[------]
SHAP5:    GCGATAGCAGTGACAGCAGCGATAGCAGTGACAGC------------------AGTGACAGCAGCAATAGCAGTGACAGCAGTGACAGCAGCGACA---------------------------GCAGTGATAGCAGTGACAGCAGTGGCAGCAGCGACAGCAGTGATAGCAGTGACAGCAGTGATAGCAGCG[------]
SHAP6:    GCGATAGCAGTGACAGCAGCGATAGCAGTGACAGC------------------AGTGACAGCAGCAATAGCAGTGACAGCAGTGACAGCAGCGACA---------------------------GCAGTGATAGCAGTGACAGCAGTGGCAGCAGCGACAGCAGTGATAGCAGTGACAGCAGTGATAGCAGCG[------]
SHAP6(2)  GCGATAGCAGTGACAGCAGCGATAGCAGTGACAGC------------------AGTGACAGCAGCAATAGCAGTGACAGCAGTGACAGCAGCGACA---------------------------GCAGTGATAGCAGTGACAGCAGTGGCAGCAGCGACAGCAGTGATAGCAGTGACAGCAGTGATAGCAGCG[------]
SHAP7     GCGATAGCAGTGACAGCAGCGATAGCAGTGACAGC------------------AGTGACAGCAGCAATAGCAGTGACAGCAGTGACAGCAGCGACA---------------------------GCAGTGATAGCAGTGACAGCAGTGACAGCAGCGACAGCAGTGATAGCAGTGAAAGCAGTGATAGCAGCG[------]
SHAP102   GCGATAGCAGTGACAGCAGCGATAGCAGTGACAGCAGCGATAGCAGTGACAGCAGTGACAGCAGCAATAGCAGTGACAGCAGTGACAGCAGCGACA---------------------------GCAGTGATAGCAGTGACAGCAGTGACAGCAGCGACAGCAGTGATAGCAGTGAAAGCAGTGATAGCAGCG[------]
SHAP130   GCGATAGCAGTGACAGCAGCGATAGCAGTGACAGC------------------AG---------CAATAGCAGTGACAGCAGTGACAGCAGCGACA---------------------------GCAGTGATAGCAGTGACAGCAGTGGCAGCAGCGACAGCAGTGATAGCAGTGACAGCAGTGATAGCAGCG[------]
SHAP106   GCGATAGCAGTGACAGCAGCGATAGCAGTGACAGC------------------AGTGACAGCAGCAATAGCAGTGACAGCAGTGACAGCAGCGACAGCAGTGACAGCAGTGACAGCAGCGACAGCAGTGATAGCAGTGACAGCAGTGGCAGCAGCGACAGCAGTGATAGCAGTGACAGCAGTGATAGCAGCG[------]
SHAP72    GCGATAGCAGTGACAGCAGCGATAGCAGTGACAGCAGCGATAGCAGTGACAGCAGTGACAGCAGCAATAGCAGTGACAGCAGTGACAGCAGCGACA---------------------------GCAGTGACAGCAGTGACAGCAGTGACAGCAGCGACAGCAGTGATAGCAGTGACAGCAGTGATAGCAGCG[------]
SHAP110   GCGATAGCAGTGACAGCAGCGATAGCAGTGACAGCAGCGATAGCAGTGACAGCAGTGACAGCAGCAATAGCAGTGACAGCAGTGACAGCAGCGACA---------------------------GCAGTGATAGCAGTGACAGCAGTGACAGCAGCGACAGCAGTGATAGCAGTGAAAGCAGTGATAGCAGCG[------]
HAP4      GCGATAGCAGTGACAGCAGCGATAGCAGTGACAGCAGCGATAGCAGTGACAGCAGTGACAGCAGCAATAGCAGTGACAGCAGTGACAGCAGCGACA---------------------------GCAGTGATAGCAGTGACAGCAGTGACAGCAGCGACAGCAGTGATAGCAGTGAAAGCAGTGATAGCAGCG[------]
HAP5      GCGATAGCAGTGACAGCAGCGATAGCAGTGACAGCAGCGATAGCAGTGACAGCAGTGACAGCAGCAATAGCAGTGACAGCAGTGACAGCAGCGACA---------------------------GCAGTGATAGCAGTGACAGCAGTGACAGCAGCGACAGCAGTGATAGCAGTGAAAGCAGTGATAGCAGCG[------]
HAP8      GCGATAGCAGTGACAGCAGCGATAGCAGTGACAGCAGCGATAGCAGTGACAGCAGTGACAGCAGCAATAGCAGTGACAGCAGTGACAGCAGCGACA---------------------------GCAGTGATAGCAGTGACAGCAGTGACAGCAGCGACAGCAGTGATAGCAGTGACAGCAGTGATAGCAGCG[------]
HAP13     GCGATAGCAGTGACAGCAGCGATAGCAGTGACAGCAGCGATAGCAGTGACAGCAGTGACAGCAGCAATAGCAGTGACAGCAGTGACAGCAGCGACA---------------------------GCAGTGATAGCAGTGACAGCAGTGACAGCAGCGACAGCAGTGATAGCAGTGACAGCAGTGATAGCAGCG[------]
HAP24     GCGATAGCAGTGACAGCAGCGATAGCAGTGACAGCAGCGATAGCAGTGACAGCAGTGACAGCAGCAATAGCAGTGACAGCAGTGACAGCAGCGACA---------------------------GCAGTGATAGCAGTGACAGCAGTGACAGCAGCGACAGCAGTGATAGCAGTGAAAGCAGTGATAGCAGTG[189 BP]
HAP25     GCGATAGCAGTGACAGCAGCGATAGCAGTGACAGCAGCGATAGCAGTGACAGCAGTGACAGCAGCAATAGCAGTGACAGCAGTGACAGCAGCGACA---------------------------GCAGTGACAGCAGTGACAGCAGTGACAGCAGCGACAGCAGTGATAGCAGTGAAAGCAGTGATAGCAGTG[------]
HAP26     GCGATAGCAGTGACAGCAGCGATAGCAGTGACAGC------------------AGTGACAGCAGCAATAGCAGTGACAGCAGTGACAGCAGCGACA---------------------------GCAGTGATAGCAGTGACAGCAGTGACAGCAGCGACAGCAGTGATAGCAGTGACAGCAGTGATAGCAGCG[------]
HAP29     GCGATAGCAGTGACAGCAGCGATAGCAGTGACAGC------------------AGTGACAGCAGCAATAGCAGTGACAGCAGTGACAGCAGCGACA---------------------------GCAGTGATAGCAGTGACAGCAGTGACAGCAGCGACAGCAGTGATAGCAGTGACAGCAGTGATAGCAGCG[------]
HAP32     GCGATAGCAGTGACAGCAGCGATAGCAGTGACAGC------------------AGTGACAGCAGCAATAGCAGTGACAGCAGTGACAGCAGCGACA---------------------------GCAGTGATAGCAGTGACAGCAGTGACAGCAGCGACAGCAGTGATAGCAGTGAAAGCAGTGATAGCAGCG[------]
HAP33     GCGATAGCAGTGACAGCAGCGATAGCAGTGACAGC------------------AGTGACAGCAGCAATAGCAGTGACAGCAGTGACAGCAGCGACA---------------------------GCAGTGATAGCAGTGACAGCAGTGGCAGCAGCGACAGCAGTGATAGCAGTGACAGCAGTGATAGCAGCG[------]
HAP35     GCGATAGCAGTGACAGCAGCGATAGCAGTGACAGC------------------AGTGACAGCAGCAATAGCAGTGACAGCAGTGACAGCAGCGACA---------------------------GCAGTGATAGCAGTGACAGCAGTGACAGCAGCGACAGCAGTGATAGCAGTGACAGCAGTGATAGCAGCG[------]
MERGED    GCGATAGCAGTGACAGCAGCGATAGCAGTGACAGCAGCGATAGCAGTGACAGCAGTGACAGCAGCAATAGCAGTGACAGCAGTGACAGCAGCGACAGCAGTGACAGCAGTGACAGCAGCGACAGCAGTGATAGCAGTGACAGCAGTGACAGCAGCGACAGCAGTGATAGCAGTGAAAGCAGTGATAGCAGTG[189 BP]
TRANSL    S  D  S  S  D  S  S  D  S  S  D  S  S  D  S  S  D  S  S  D  S  S  N  S  S  D  S  S  D  S  S  D  S  S  D  S  S  D  S  S  D  S  S  D  S  S  D  S  S D/G S  S  D  S  S  D  S  S E/D S  S  D  S  S   [63 AA]

HAP24     ACAGCAGCAATAGCAGTGACAGCAGTGACAGCAGCGACAGCAGTGATAGCAGTGACAGCAGCGATAGCAGTGACAGCAGTGACAGCAGCAATAGCAGTGACAGCAGTGACAGCAGCGACAGCAGTGATAGCAGTGACAGCAGTGACAGCAGCGACAGCAGTGATAGCAGTGAAAGCAGTGATAGCAGTG
MERGED    ACAGCAGCAATAGCAGTGACAGCAGTGACAGCAGCGACAGCAGTGATAGCAGTGACAGCAGCGATAGCAGTGACAGCAGTGACAGCAGCAATAGCAGTGACAGCAGTGACAGCAGCGACAGCAGTGATAGCAGTGACAGCAGTGACAGCAGCGACAGCAGTGATAGCAGTGAAAGCAGTGATAGCAGTG
MERGED    D  S  S  N  S  S  D  S  S  D  S  S  D  S  S  D  S  S  D  S  S  D  S  S  D  S  S  D  S  S  N  S  S  D  S  S  D  S  S  D  S  S  D  S  S  D  S  S  D  S  S  D  S  S  E  S  S  D  S  S
```

Notes:

The [189 bp] at the end of the alignment for Hap24 refers to the sequence immediately above these notes.

SNPs: multiple haplotypes: c.3221A>G (p.Asp1074Gly); c.3249A>C (p.Glu1083Asp); c.3264T>C.

ID15: NM_014208.3:c.3136_3153delAGCGATAGCAGTGACAGC.

ID16: NM_014208.3:c.3156_3164delTGACAGCAG.

ID17: NM_014208.3:c.3170_3196dup (Same as designation: NM_014208.3:c.3196_3197insGCAGTGACAGCAGTGACAGCAGCGACA).

ID18: NM_014208.3:c.3219_3227delTGACAGCAG.

ID19: NM_014208.3:c.3257_3445delATAGCAGTGATAGCAGTGACAGCAGCAACAGCAGTGACAGCAGTGACAGCAGTG.

ID20: NM_014208.3:c.3265_3266insACAGCAGCAATAGCAGTGACAGCAGTGACAGCAGCGACAGCAGTGATAGCAGTGACAGCAGCGATAGCAGTGACAGCAGTGACAG CAGCAATAGCAGTGACAGCAGTGACAGCAGCGACAGCAGTGATAGCAGTGACAGCAGTGACAGCAGCGACAGCAGTGATAGCAGTGAAAGCAGTGATAGCAGT.

Mutations:

18. g.12269delC; c.3135delC; p.(Ser1045Argfs*269); Family 10 [1,10]

```
          3266                                                                  ID21                                                                                                           ID19 continued   ID22 3438   3447        ID23
REFSEQ    ACAGCAGCAATAGCAGTGACAGCAGCGATAGCAGCGACAGCAGCGACAGCAGCGATAGCAGTGACAGCAGCGATAGCAGTGACAGCAGTGACAGCAGCAATAGCAGTGACAGCAGTGACAGCAGCGACAGCAGTGATAGCAGTGACAGCAGCAACAGCAGTGACAGCAGTGACAGCAGTGAA------------------
Fam8a     ---------------------------------------------------------------------------------------------------------------------------------------ATAGCAGTGACAGCAGCGACAGCAGTGACAGCAGTGACAGCAGTGAAAGCAGCGACAGCAGCGAT
Fam8b     ---------------------------------------------------------------------------------------------------------------------------------------ATAGCAGTGACAGCAGCGACAGCAGTGACAGCAGTGACAGCAGTGAA------------------
Fam9a     ---------------------------------------------------------------------------------------------------------------------------------------ATAGCAGTGACAGCAGCGACAGCAGTGACAGCAGTGACAGCAGTGAA------------------
Fam9b     ---------------------------------------------------------------------------------------------------------------------------------------ATAGCAGTGACAGCAGCGACAGCAGTGACAGCAGTGACAGCAGTGAA------------------
Fam10a    ---------------------------------------------------------------------------------------------------------------------------------------ATAGCAGTGACAGCAGCGACAGCAGTGACAGCAGTGACAGCAGTGAAAGCAGCGACAGCAGCGAT
Fam10b    ---------------------------------------------------------------------------------------------------------------------------------------ATAGCAGTGACAGCAGCGACAGCAGTGACAGCAGTGACAGCAGTGAAAGCAGCGACAGCAGCGAT
Fam11a    ---------------------------------------------------------------------------------------------------------------------------------------ATAGCAGTGACAGCAGCGACAGCAGTGACAGCAGTGACAGCAGTGAAAGCAGCGACAGCAGCGAT
Fam11b    ---------------------------------------------------------------------------------------------------------------------------------------ATAGCAGTGACAGCAGCGACAGCAGTGACAGCAGTGACAGCAGTGAAAGCAGCGACAGCAGCGAT
Fam12a    ---------------------------------------------------------------------------------------------------------------------------------------ATAGCAGTGACAGCAGCGACAGCAGTGACAGCAGTGACAGCAGTGAAAGCAGCGACAGCAGCGAT
Fam12b    ---------------------------------------------------------------------------------------------------------------------------------------ATAGCAGTGACAGCAGCGACAGCAGTGACAGCAGTGACAGCAGTGAAAGCAGCGACAGCAGCGAT
F1A       ---------------------------------------------------------------------------------------------------------------------------------------ATAGCAGTGACAGCAGCGACAGCAGTGACAGCAGTGACAGCAGTGAAAGCAGCGACAGCAGCGAT
F1B       ---------------------------------------------------------------------------------------------------------------------------------------ATAGCAGTGACAGCAGCGACAGCAGTGACAGCAGTGACAGCAGTGAAAGCAGCGACAGCAGCGAT
F2A       ---------------------------------------------------------------------------------------------------------------------------------------ATAGCAGTGACAGCAGCGACAGCAGTGACAGCAGTGACAGCAGTGAAAGCAGCGACAGCAGCGAT
F2B       ACAGCAGCAATAGCAGTGACAGCAGCGATAGCAGCGACAGCAGCGACAGCAGCGATAGCAGTGACAGCAGCGATAGCAGTGACAGCAGTGACAGCAGCAATAGCAGTGACAGCAGTGACAGCAGCGACAGCAGTGATAGCAGTGACAGCAGCAACAGCAGTGACAGCAGTGACAGCAGTGAA------------------
F3A       ---------------------------------------------------------------------------------------------------------------------------------------ATAGCAGTGACAGCAGCGACAGCAGTGACAGCAGTGACAGCAGTGAAAGCAGCGACAGCAGCGAT
F3B       ---------------------------------------------------------------------------------------------------------------------------------------ATAGCAGTGACAGCAGCGACAGCAGTGACAGCAGTGACAGCAGTGAA------------------
F4A       ------------------------------------------------------------------------------------------------------------------------------------------------------------------------------------AAAGCAGCGACAGCAGCGAT
F4B       ACAGCAGCAATAGCAGTGACAGCAGCGATAGCAGCGACAGCAGCGACAGCAGCGATAGCAGTGACAGCAGCGATAGCAGTGACAGCAGTGACAGCAGCAATAGCAGTGACAGCAGTGACAGCAGCGACAGCAGTGATAGCAGTGACAGCAGCAACAGCAGTGACAGCAGTGACAGCAGTGAA------------------
F5A       ---------------------------------------------------------------------------------------------------------------------------------------ATAGCAGTGACAGCAGCGACAGCAGTGACAGCAGTGACAGCAGTGAA------------------
F5B       ---------------------------------------------------------------------------------------------------------------------------------------ATAGCAGTGACAGCAGCGACAGCAGTGACAGCAGTGACAGCAGTGAAAGCAGCGACAGCAGCGAT
HAP1A     ---------------------------------------------------------------------------------------------------------------------------------------ATAGCAGTGACAGCAGCGACAGCAGTGACAGCAGTGACAGCAGTGAAAGCAGCGACAGCAGCGAT
HAP2A     ---------------------------------------------------------------------------------------------------------------------------------------ATAGCAGTGACAGCAGCGACAGCAGTGACAGCAGTGACAGCAGTGAA------------------
HAP3A     ACAGCAGCAATAGCAGTGACAGCAGCGATAGCAGCGACAGCAGCGACAGCAGCGATAGCAGTGACAGCAGCGATAGCAGTGACAGCAGTGACAGCAGCAATAGCAGTGACAGCAGTGACAGCAGCGACAGCAGTGATAGCAGTGACAGCAGCAACAGCAGTGACAGCAGTGACAGCAGTGAA------------------
HAP15A    ---------------------------------------------------------------------------------------------------------------------------------------ATAGCAGTGACAGCAGCGACAGCAGTGACAGCAGTGACAGCAGTGAAAGCAGCGACAGCAGCGAT
HAP17B    ---------------------------------------------------------------------------------------------------------------------------------------ATAGCAGTGACAGCAGCGACAGCAGTGACAGCAGTGACAGCAGTGAAAGCAGCGACAGCAGCGAT
HAP20A    ---------------------------------------------------------------------------------------------------------------------------------------ATAGCAGTGACAGCAGCGACAGCAGTGACAGCAGTGACAGCAGTGAA------------------
HAP20B    ---------------------------------------------------------------------------------------------------------------------------------------ATAGCAGTGACAGCAGCGACAGCAGTGACAGCAGTGACAGCAGTGAAAGCAGCGACAGCAGCGAT
HAP36A    ---------------------------------------------------------------------------------------------------------------------------------------ATAGCAGTGACAGCAGCGACAGCAGTGACAGCAGTGACAGCAGTGAA------------------
HAP37A    ---------------------------------------------------------------------------------------------------------------------------------------ATAGCAGTGACAGCAGCGACAGCAGTGACAGCAGTGACAGCAGTGAAAGCAGCGACAGCAGCGAT
HAP38A    ---------------------------------------------------------------------------------------------------------------------------------------ATAGCAGTGACAGCAGCGACAGCAGTGACAGCAGTGACAGCAGTGAA------------------
SHAP1:    ---------------------------------------------------------------------------------------------------------------------------------------ATAGCAGTGACAGCAGCGACAGCAGTGACAGCAGTGACAGCAGTGAAAGCAGCGACAGCAGCGAT
SHAP2:    ACAGCAGCAATAGCAGTGACAGCAGCGATAGCAGCGACAGCAGCGACAGCAGCGATAGCAGTGACAGCAGCGATAGCAGTGACAGCAGTGACAGCAGCAATAGCAGTGACAGCAGTGACAGCAGCGACAGCAGTGATAGCAGTGACAGCAGCAACAGCAGTGACAGCAGTGACAGCAGTGAA------------------
SHAP3:    ---------------------------------------------------------------------------------------------------------------------------------------ATAGCAGTGACAGCAGCGACAGCAGTGACAGCAGTGACAGCAGTGAAAGCAGCGACAGCAGCGAT
SHAP4:    ---------------------------------------------------------------------------------------------------------------------------------------ATAGCAGTGACAGCAGCGACAGCAGTGACAGCAGTGACAGCAGTGAAAGCAGCGACAGCAGCGAT
SHAP5:    ---------------------------------------------------------------------------------------------------------------------------------------ATAGCAGTGACAGCAGCGACAGCAGTGACAGCAGTGACAGCAGTGAAAGCAGCGACAGCAGCGAT
SHAP6:    ---------------------------------------------------------------------------------------------------------------------------------------ATAGCAGTGACAGCAGCGACAGCAGTGACAGCAGTGACAGCAGTGAAAGCAGCGACAGCAGCGAT
SHAP6(2)  ---------------------------------------------------------------------------------------------------------------------------------------ATAGCAGTGACAGCAGCGACAGCAGTGACAGCAGTGACAGCAGTGAAAGCAGCGACAGCAGCGAT
SHAP7     ---------------------------------------------------------------------------------------------------------------------------------------ATAGCAGTGACAGCAGCGACAGCAGTGACAGCAGTGACAGCAGTGAAAGCAGCGACAGCAGCGAT
SHAP102   ---------------------------------------------------------------------------------------------------------------------------------------ATAGCAGTGACAGCAGCGACAGCAGTGACAGCAGTGACAGCAGTGAAAGCAGCGACAGCAGCGAT
SHAP130   ---------------------------------------------------------------------------------------------------------------------------------------ATAGCAGTGACAGCAGCGACAGCAGTGACAGCAGTGACAGCAGTGAAAGCAGCGACAGCAGCGAT
SHAP106   ---------------------------------------------------------------------------------------------------------------------------------------ATAGCAGTGACAGCAGCGACAGCAGTGACAGCAGTGACAGCAGTGAAAGCAGCGACAGCAGCGAT
SHAP72    ---------------------------------------------------------------------------------------------------------------------------------------ATAGCAGTGACAGCAGCGACAGCAGTGACAGCAGTGACAGCAGTGAA------------------
SHAP110   ---------------------------------------------------------------------------------------------------------------------------------------ATAGCAGTGACAGCAGCGACAGCAGTGACAGCAGTGACAGCAGTGAAAGCAGCGACAGCAGCGAT
HAP4      ---------------------------------------------------------------------------------------------------------------------------------------ATAGCAGTGACAGCAGCGACAGCAGTGACAGCAGTGACAGCAGTGAA------------------
HAP5      ---------------------------------------------------------------------------------------------------------------------------------------ATAGCAGTGACAGCAGCGACAGCAGTGACAGCAGTGACAGCAGTGAA------------------
HAP8      ---------------------------------------------------------------------------------------------------------------------------------------ATAGCAGTGACAGCAGCGACAGCAGTGACAGCAGTGACAGCAGTGAAAGCAGCGACAGCAGCGAT
HAP13     ---------------------------------------------------------------------------------------------------------------------------------------ATAGCAGTGACAGCAGCGACAGCAGTGAC------------------AGCAGCGACAGCAGCGAT
HAP24     ACAGCAGCAATAGCAGTGACAGCAGCGATAGCAGCGACAGCAGCGACAGCAGCGATAGCAGTGACAGCAGCGATAGCAGTGACAGCAGTGACAGCAGCAATAGCAGTGACAGCAGTGACAGCAGCGACAGCAGTGATAGCAGTGACAGCAGCAACAGCAGTGACAGCAGTGACAGCAGTGAA------------------
HAP25     ACAGCAGCAATAGCAGTGACAGCAGCGATAGCAGCGACAGCAGCGACAGCAGCGATAGCAGTGACAGCAGCGATAGCAGTGACAGCAGTGACAGCAGCAATAGCAGTGACAGCAGTGACAGCAGCGACAGCAGTGATAGCAGTGACAGCAGCAACAGCAGTGACAGCAGTGACAGCAGTGAA------------------
HAP26     ---------------------------------------------------------------------------------------------------------------------------------------ATAGCAGTGACAGCAGCGACAGCAGTGACAGCAGTGACAGCAGTGAAAGCAGCGACAGCAGCGAT
HAP29     ---------------------------------------------------------------------------------------------------------------------------------------ATAGCAGTGACAGCAGCGACAGCAGTGACAGCAGTGACAGCAGTGAAAGCAGCGACAGCAGCGAT
HAP32     ---------------------------------------------------------------------------------------------------------------------------------------ATAGCAGTGACAGCAGCGACAGCAGTGACAGCAGTGACAGCAGTGAA------------------
HAP33     ---------------------------------------------------------------------------------------------------------------------------------------ATAGCAGTGACAGCAGCGACAGCAGTGACAGCAGTGACAGCAGTGAAAGCAGCGACAGCAGCGAT
HAP35     ---------------------------------------------------------------------------------------------------------------------------------------ATAGCAGTGACAGCAGCGACAGCAGTGACAGCAGTGACAGCAGTGAAAGCAGCGACAGCAGCGAT
MERGED    ACAGCAGCAATAGCAGTGACAGCAGCGATAGCAGCGACAGCAGCGACAGCAGCGATAGCAGTGACAGCAGCGATAGCAGTGACAGCAGTGACAGCAGCAATAGCAGTGACAGCAGTGACAGCAGCGACAGCAGTGATAGCAGTGACAGCAGCAACAGCAGTGACAGCAGTGACAGCAGTGAAAGCAGCGACAGCAGCGAT
TRANSL    D  S  S  N  S  S  D  S  S  D  S  S  D  S  S  D  S  S  D  S  S  D  S  S  D  S  S  D  S  S  N  S  S  D  S  S  D  S  S  D  S  S  D  S  S  D  S  S N/D S  S  D  S  S  D  S  S  E  S  S  D  S  S  D
```

Notes:

SNP: c.3418A>G (p.(Asn1140Asp);

ID21: NM_014208.3:c.3266_3400delACAGCAGCAATAGCAGTGACAGCAGCGATAGCAGCGACAGCAGCGACAGCAGCGATAGC AGTGACAGCAGCGATAGCAGTGACAGCAGTGACAGCAGCAATAGCAGTGACAGCAGTGACAGCAGCGACAGCAGTG.

ID22: NM_014208.3:c.3430_3447delAGCAGTGACAGCAGTGAA.

ID23: NM_014208.3:c.3447_3448insAGCAGCGACAGCAGCGAT.

Mutations:

20. g.12572delC; c.3438delC; p.(Asp1146Glufs*168) [4]

```
          3448          3461        ID24   3480/3481                    3504_3508   ID25   3509_3521                          3546-3550     3560           ID26     3582-3591                              ID27               3624
REFSEQ    AGCAGCGACAGCAGTGACAGCAGCGACAGCAGTGACAGCAGCGATAGCAGCGACAGCAGCG-----ACAGCAGCGATAGCAGTGACAGCAGCAATAGCAGTGATAGCAGCGACAGCAGTGATAGCAGTGACAGCAGCGACAGCAGCGATAGCAGCGACAGCAGCGATAG------------------TAGTGATAGCAGT
Fam8a     AGCAGCGACAGCAGTGACAGC------------------------AGCAGCGACAGCAGCG-----ACAGCAGCGATAGCAGTGACAGCAGCAATAGCAGCGATAGCAGCGACAGCAGTGATAGCAGCGACAGCAGCGACAGCAGCGACAGCAGCGACAGCAGTGATAG------------------TAGTGATAGCAGT
Fam8b     AGCAGCAACAGCAGTGACAGCAGCGACAGCAGTGACAGCAGCGATAGCAGCGACAGCAGCG-----ACAGCAGCGATAGCAGTGACAGCAGCAATAGCAGTGATAGCAGCGACAGCAGTGATAGCAGTGACAGCAGCGACAGCAGCGATAGCAGCGACAGCAGCGATAG------------------TAGTGATAGCAGT
Fam9a     AGCAGCAACAGCAGTGACAGCAGCGACAGCAGTGACAGCAGCGATAGCAGCGACAGCAGCG-----ACAGCAGCGATAGCAGTGACAGCAGCAATAGCAGTGATAGCAGCGACAGCAGTGATAGCAGTGACAGCAGCGACAGCAGCGATAGCAGCGACAGCAGCGATAG------------------TAGTGATAGCAGT
Fam9b     AGCAGCAACAGCAGTGACAGCAGCGACAGCAGTGACAGCAGCGATAGCAGCGACAGCAGCG-----ACAGCAGCGATAGCAGTGACAGCAGCAATAGCAGTGATAGCAGCGACAGCAGTGATAGCAGTGACAGCAGCGACAGCAGCGATAGCAGCGACAGCAGCGATAG------------------TAGTGATAGCAGT
Fam10a    AGCAGCGACAGCAGTGACAGCAGCGACAGCAGTGACAGCAGCGATAGCAGCGACAGCAGCG-----ACAGCAGCGATAGCAGTGACAGCAGCAATAGCAGTGATAGCAGCGACAGCAGTGATAGCAGTGACAGCAGCGACAGCAGCGATAGCAGCGACAGCAGTGATAG------------------TAGTGATAGCAGT
Fam10b    AGCAGCGACAGCAGTGAC---------------------------AGCAGCGACAGCAGCG-----ACAGCAGCGATAGCAGTGACAGCAGCAATAGCAGTGATAGCAGCGACAGCAGTGATAGCAGCGACAGCAGCGACAGCAGCGACAGCAGCGACAGCAGTGATAG------------------TAGTGATAGCAGT
Fam11a    AGCAGCGACAGCAGTGAC---------------------------AGCAGCGACAGCAGCG-----ACAGCAGCGATAGCAGTGACAGCAGCAATAGCAGCGATAGCAGCGACAGCAGTGATAGCAGCGACAGCAGCGACAGCAGCGACAGCAGCGACAGCAGTGATAG------------------TAGTGATAGCAGT
Fam11b    AGCAGCGACAGCA-TGAC---------------------------AGCAGCGACAGCAGCG-----ACAGCAGCGATAGCAGTGACAGCAGCAATAGCAGTGATAGCAGCGACAGCAGTGATAGCAGCGACAGCAGCGACAGCAGCGACAGCAGCGACAGCAGTGATAG------------------TAGTGATAGCAGT
Fam12a    AGCAGCGACAGCAGTGACAGCAGCGACAGCAGTGACAGCAGCGATAGCAGCGACAGCAGCG-----ACAGCAGCGATAGCAGTGACAGCAGCAATAGCAGTGATAGCAGCGACAGCAGTGATAGCAGTGACAGCAGCGACAGCAGCGATAGCAGCGACAGCAGTGATAG------------------TAGTGATAGCAGT
Fam12b    AGCAGCGACAGCAGTGAC---------------------------AGCAGCGACAGCAGCG-----ACAGCAGCGATAGCAGTGACAGCAGCAATAGCAGCGATAGCAGCGACAGCAGTGATAGCAGCGACAGCAGCGACAGCAGCGACAGCAGCGACAGCAGTGATAG------------------TAGTGATAGCAGT
F1A       AGCAGCGACAGCAGTGACAGCAGCGACAGCAGTGACAGCAGCGATAGCAGCGACAGCAGCG-----ACAGCAGCGATAGCAGTGACAGCAGCAATAGCAGTGATAGCAGCGACAGCAGTGATAGCAGTGACAGCAGCGACAGCAGCGATAGCAGCGACAGCAGTGATAG------------------TAGTGATAGCAGT
F1B       AGCAGCGACAGCAGTGACAGCAGCGACAGCAGTGACAGCAGCGATAGCAGCGACAGCAGCG-----ACAGCAGCGATAGCAGTGACAGCAGCAATAGCAGTGATAGCAGCGACAGCAGTGATAGCAGTGACAGCAGCGACAGCAGCGATAGCAGCGACAGCAGTGATAG------------------TAGTGATAGCAGT
F2A       AGCAGCGACAGCAGTGACAGCAGCGACAGCAGCGACAGCAGCGATAGCAGTGACAGCAGCGCAGCGACAGCAGCGATAGCAGTGACAGCAGCAATAGCAGCGATAGCAGCGACAGCAGTGATAGCAGCGACAGCAGCGACAGCAGCGACAGCAGCGACAGCAGTGATAG------------------TAGTGATAGCAGT
F2B       AGCAGCGACAGCAGTGACAGCAGCGACAGCAGTGACAGCAGCGATAGCAGCGACAGCAGCG-----ACAGCAGCGATAGCAGTGACAGCAGCAATAGCAGTGATAGCAGCGACAGCAGTGATAGCAGTGACAGCAGCGACAGCAGCGATAGCAGCGACAGCAGCGATAG------------------TAGTGATAGCAGT
F3A       AGCAGCGACAGCAGTGAC---------------------------AGCAGCGACAGCAGCG-----ACAGCAGCGATAGCAGTGACAGCAGCAATAGCAGCGATAGCAGCGACAGCAGTGATAGCAGCGACAGCAGCGACAGCAGCGACAGCAGCGACAGCAGTGATAG------------------TAGTGATAGCAGT
F3B       AGCAGCAACAGCAGTGACAGCAGCGACAGCAGTGACAGCAGCGATAGCAGCGACAGCAGCG-----ACAGCAGCGATAGCAGTGACAGCAGCAATAGCAGTGATAGCAGCGACAGCAGTGATAGCAGTGACAGCAGCGACAGCAGCGATAGCAGCGACAGCAGCGATAG------------------TAGTGATAGCAGT
F4A       AGCAGCGACAGCAGTGACAGCAGCGACAGCAGTGACAGCAGCGATAGCAGCGACAGCAGCG--------------ATAGCAGTGACAGCAGCAATAGCAGTGATAGCAGCGACAGCAGTGATAGCAGTGACAGCAGCGACAGCAGCGATAGCAGCGACAGCAGTGATAG------------------TAGTGATAGCAGT
F4B       AGCAGCGACAGCAGTGACAGCAGCGACAGCAGTGACAGCAGCGATAGCAGCGACAGCAGCG-----ACAGCAGCGATAGCAGTGACAGCAGCAATAGCAGTGATAGCAGCGACAGCAGTGATAGCAGTGACAGCAGCGACAGCAGCGATAGCAGCGACAGCAGCGATAG------------------TAGTGATAGCAGT
F5A       AGCAGCAACAGCAGTGACAGCAGCGACAGCAGTGACAGCAGCGATAGCAGCGACAGCAGCG-----ACAGCAGCGATAGCAGTGACAGCAGCAATAGCAGTGATAGCAGCGACAGCAGTGATAGCAGTGACAGCAGCGACAGCAGCGATAGCAGCGACAGCAGCGATAG------------------TAGTGATAGCAGT
F5B       AGCAGCGACAGCAGTGAC---------------------------AGCAGCGACAGCAGCG-----ACAGCAGCGATAGCAGTGACAGCAGCAATAGCAGCGATAGCAGCGACAGCAGTGATAGCAGCGACAGCAGCGACAGCAGCGACAGCAGCGACAGCAGTGATAG------------------TAGTGATAGCAGT
HAP1A     AGCAGCGACAGCAGTGACAGCAGCGACAGCAGTGACAGCAGCGATAGCAGCGACAGCAGCG-----ACAGCAGCGATAGCAGTGACAGCAGCAATAGCAGTGATAGCAGCGACAGCAGTGATAGCAGTGACAGCAGCGACAGCAGCGATAGCAGCGACAGCAGTGATAG------------------TAGTGATAGCAGT
HAP2A     AGCAGCAACAGCAGTGACAGCAGCGACAGCAGTGACAGCAGCGATAGCAGCGACAGCAGCG-----ACAGCAGCGATAGCAGTGACAGCAGCAATAGCAGTGATAGCAGCGACAGCAGTGATAGCAGTGACAGCAGCGACAGCAGCGATAGCAGCGACAGCAGCGATAG------------------TAGTGATAGCAGT
HAP3A     AGCAGCGACAGCAGTGACAGCAGCGACAGCAGTGACAGCAGCGATAGCAGCGACAGCAGCG-----ACAGCAGCGATAGCAGTGACAGCAGCAATAGCAGTGATAGCAGCGACAGCAGTGATAGCAGCGACAGCAGTGACAGCAGCGATAGCAGCGACAGCAGCGATAGCAGCGACAGCAGCGATAGTAGTGATAGCAGT
HAP15A    AGCAGCGACAGCAGTGAC---------------------------AGCAGCGACAGCAGCG-----ACAGCAGCGATAGCAGTGACAGCAGCAATAGCAGCGATAGCAGCGACAGCAGTGATAGCAGCGACAGCAGCGACAGCAGCGACAGCAGCGACAGCAGTGATAG------------------TAGTGATAGCAGT
HAP17B    AGCAGCGACAGCAGTGAC---------------------------AGCAGCGACAGCAGCG-----ACAGCAGCGATAGCAGTGACAGCAGCAATAGCAGTGATAGCAGCGACAGCAGTGATAGCAGCGACAGCAGCGACAGCAGCGACAGCAGCGACAGCAGTGATAG------------------TAGTGATAGCAGT
HAP20A    AGCAGCGACAGCAGTGACAGCAGCGACAGCAGTGACAGCAGCGATAGCAGCGACAGCAGCG-----ACAGCAGCGATAGCAGTGACAGCAGCAATAGCAGTGATAGCAGCGACAGCAGTGACAGCAGTGACAGCAGCGACAGCAGCGATAGCAGCGACAGCAGTGACAG------------------TAGTGATAGCAG-
HAP20B    AGCAGCGACAGCAGTGACAGCAGCGACAGCAGTGACAGCAGCGATAGCAGCGACAGCAGCG-----ACAGCAGCGATAGCAGTGACAGCAGCAATAGCAGTGATAGCAGCGACAGCAGTGATAGCAGTGACAGCAGCGACAGCAGCGATAGCAGCGACAGCAGTGATAG------------------TAGTGATAGCAGT
HAP36A    AGCAGCGACAGCAGTGACAGCAGCGACAGCAGTGACAGCAGCGATAGCAGCGACAGCAGCG-----ACAGCAGCGATAGCAGTGACAGCAGCGACAGCAGTGATAGCAGCGACAGCAGTGACAGCAGTGACAGCAGCGACAGCAGCGATAGCAGCGACAGCAGTGACAG------------------TAGTGATAGCAG-
HAP37A    AGCAGCGACAGCAGTGACAGCAGCGACAGCAGTGACAGCAGCGATAGCAGCGACAGCAGCG-----ACAGCAGCGATAGCAGTGACAGCAGCAATAGCAGTGATAGCAGCGACAGCAGTGATAGCAGTGACAGCAGCGACAGCAGCGATAGCAGCGACAGCAGTGATAG------------------TAGTGATAGCAGT
HAP38A    AGCAGCGACAGCAGTGACAGCAGCGACAGCAGTGACAGCAGCGATAGCAGCGACAGCAGCG-----ACAGCAGCGATAGCAGTGACAGCAGCAATAGCAGTGATAGCAGCGACAGCAGTGATAGCAGTGACAGCAGCGACAGCAGCGATAGCAGCGACAGCAGTGATAG------------------TAGTGATAGCAG-
SHAP1:    AGCAGCGACAGCAGTGACAGCAGCGACAGCAGTGACAGCAGCGATAGCAGCGACAGCAGCG-----ACAGCAGCGATAGCAGTGACAGCAGCAATAGCAGTGATAGCAGCGACAGCAGTGATAGCAGTGACAGCAGCGACAGCAGCGATAGCAGCGACAGCAGTGATAG------------------TAGTGATAGCAGT
SHAP2:    AGCAGCGACAGCAGTGACAGCAGCGACAGCAGTGACAGCAGCGATAGCAGCGACAGCAGCG-----ACAGCAGCGATAGCAGTGACAGCAGCAATAGCAGTGATAGCAGCGACAGCAGTGATAGCAGTGACAGCAGCGACAGCAGCGATAGCAGCGACAGCAGCGATAG------------------TAGTGATAGCAGT
SHAP3:    AGCAGCGACAGCAGTGACAGCAGCGACAGCAGTGACAGCAGCGATAGCAGCGACAGCAGCG-----ACAGCAGCGATAGCAGTGACAGCAGCAATAGCAGTGATAGCAGCGACAGCAGTGATAGCAGCGACAGCAGCGACAGCAGCGACAGCAGCGACAGCAGTGATAG------------------TAGTGATAGCAGT
SHAP4:    AGCAGCAACAGCAGTGACAGCAGCGACAGCAGTGACAGCAGCGATAGCAGCGACAGCAGCG-----ACAGCAGCGATAGCAGTGACAGCAGCAATAGCAGTGATAGCAGCGACAGCAGTGATAGCAGTGACAGCAGCGACAGCAGCGATAGCAGCGACAGCAGCGATAG------------------TAGTGATAGCAGT
SHAP5:    AGCAGCGACAGCAGTGACAGCAGCGACAGCAGTGACAGCAGCGATAGCAGCGACAGCAGCG-----ACAGCAGCGATAGCAGTGACAGCAGCAATAGCAGTGATAGCAGCGACAGCAGTGATAGCAGTGACAGCAGCGACAGCAGCGATAGCAGCGACAGCAGTGATAG------------------TAGTGATAGCAG-
SHAP6:    AGCAGCGACAGCAGTGACAGCAGCGACAGCAGTGACAGCAGCGATAGCAGCGACAGCAGCG-----ACAGCAGCGATAGCAGTGACAGCAGCAATAGCAGTGATAGCAGCGACAGCAGTGATAGCAGTGACAGCAGCGACAGCAGCGATAGCAGCGACAGCAGTGATAG------------------TAGTGATAGCAGT
SHAP6(2)  AGCAGCGACAGCAGTGACAGCAGCGACAGCAGTGACAGCAGCGATAGCAGCGACAGCAGCG-----ACAGCAGCGATAGCAGTGACAGCAGCAATAGCAGTGATAGCAGCGACAGCAGTGATAGCAGTGACAGCAGCGACAGCAGCGATAGCAGCGACAGCAGTGATAG------------------TAGTGATAGCAGT
SHAP7     AGCAGCAACAGCAGTGACAGCAGCGACAGCAGTGACAGCAGCGATAGCAGCGACAGCAGCG-----ACAGCAGCGATAGCAGTGACAGCAGCAATAGCAGTGATAGCAGCGACAGCAGTGATAGCAGTGACAGCAGCGACAGCAGCGATAGCAGCGACAGCAGCGATAG------------------TAGTGATAGCAGT
SHAP102   AGCAGCGACAGCAGTGACAGCAGCGACAGCAGTGACAGCAGCGATAGCAGCGACAGCAGCG-----ACAGCAGCGATAGCAGTGACAGCAGCAATAGCAGTGATAGCAGCGACAGCAGTGATAGCAGTGACAGCAGCGACAGCAGCGATAGCAGCGACAGCAGTGATAG------------------TAGTGATAGCAG-
SHAP130   AGCAGCGACAGCAGTGACAGCAGCGACAGCAGTGACAGCAGCGATAGCAGCGACAGCAGCG-----ACAGCAGCGATAGCAGTGACAGCAGCAATAGCAGTGATAGCAGCGACAGCAGTGATAGCAGTGACAGCAGCGACAGCAGCGATAGCAGCGACAGCAGTGATAG------------------TAGTGATAGCAGT
SHAP106   AGCAGCGACAGCAGTGACAGCAGCGACAGCAGTGACAGCAGCGATAGCAGCGACAGCAGCG-----ACAGCAGCGATAGCAGTGACAGCAGCAATAGCAGTGATAGCAGCGACAGCAGTGATAGCAGTGACAGCAGCGACAGCAGCGATAGCAGCGACAGCAGTGATAG------------------TAGTGATAGCAGT
SHAP72    AGCAGCGACAGCAGTGAC---------------------------AGCAGCGACAGCAGCG-----ACAGCAGCGATAGCAGTGACAGCAGCAATAGCAGTGATAGCAGCGACAGCAGTGATAGCAGTG---------ACAGCAGCGACAGCAGCGACAGCAGTGATAG------------------TAGTGATAGCAGT
SHAP110   AGCAGCAACAGCAGTGACAGCAGCGACAGCAGTGACAGCAGCGATAGCAGCGACAGCAGCG-----ACAGCAGCGATAGCAGTGACAGCAGCAATAGCAGTGATAGCAGCGACAGCAGTGATAGCAGTGACAGCAGCGACAGCAGCGATAGCAGCGACAGCAGCGATAG------------------TAGTGATAGCAG-
HAP4      AGCAGCGACAGCAGTGACAGCAGCGACAGCAGTGACAGCAGCGATAGCAGCGACAGCAGCG-----ACAGCAGCGATAGCAGTGACAGCAGCAATAGCAGTGATAGCAGCGACAGCAGTGACAGCAGTG---------ACAGCAGCGATAGCAGCGACAGCAGTGACAG------------------TAGTGATAGCAG-
HAP5      AGCAGCGACAGCAGTGACAGCAGCGACAGCAGTGACAGCAGCGATAGCAGCGACAGCAGCG-----ACAGCAGCGATAGCAGTGACAGCAGCAATAGCAGTGATAGCAGCGACAGCAGTGACAGCAGTG---------ACAGCAGCGATAGCAGCGACAGCAGTGACAG------------------TAGTGATAGCAG-
HAP8      AGCAGCGACAGCAGTGACAGCAGCGACAGCAGTGACAGCAGCGATAGCAGCGACAGCAGCG-----ACAGCAGCGATAGCAGTGACAGCAGCAATAGCAGTGATAGCAGCGACAGCAGTGATAGCAGTGACAGCAGCGACAGCAGCGATAGCAGCGACAGCAGTGATAG------------------TAGTGATAGCAG-
HAP13     AGCAGCGACAGCAGTGAC---------------------------AGCAGCGACAGCAGCG-----ACAGCAGCGATAGCAGTGACAGCAGCAATAGCAGTGATAGCAGCGACAGCAGTGATAGCAGCGACAGCAGCGACAGCAGCGACAGCAGCGACAGCAGTGATAG------------------TAGTGATAGCAGT
HAP24     AGCAGCGACAGCAGTGACAGCAGCGACAGCAGTGACAGCAGCGATAGCAGCGACAGCAGCG-----ACAGCAGCGATAGCAGTGACAGCAGCAATAGCAGTGATAGCAGCGACAGCAGTGATAGCAGTGACAGCAGCGACAGCAGCGATAGCAGCGACAGCAGCGATAG------------------TAGTGATAGCAGT
HAP25     AGCAGCGACAGCAGTGACAGCAGCGACAGCAGTGACAGCAGCGATAGCAGCGACAGCAGCG-----ACAGCAGCGATAGCAGTGACAGCAGCAATAGCAGTGATAGCAGCGACAGCAGTGATAGCAGTGACAGCAGCGACAGCAGCGATAGCAGCGACAGCAGCGATAG------------------TAGTGATAGCAGT
HAP26     AGCAGCGACAGCAGTGACAGCAGCGACAGCAGTGACAGCAGCGATAGCAGCGACAGCAGCG-----ACAGCAGCGATAGCAGTGACAGCAGCAATAGCAGTGATAGCAGCGACAGCAGTGATAGCAGTGACAGCAGCGACAGCAGCGATAGCAGCGACAGCAGTGATAG------------------TAGTGATAGCAGT
HAP29     AGCAGCGACAGCAGTGACAGCAGCGACAGCAGTGACAGCAGCGATAGCAGCGACAGCAGCG-----ACAGCAGCGATAGCAGTGACAGCAGCAATAGCAGTGATAGCAGCGACAGCAGTGATAGCAGTGACAGCAGCGACAGCAGCGATAGCAGCGACAGCAGTGATAG------------------TAGTGATAGCAGT
HAP32     AGCAGCGACAGCAGTGACAGCAGCGACAGCAGTGACAGCAGCGATAGCAGCGACAGCAGCG-----ACAGCAGCGATAGCAGTGACAGCAGCAATAGCAGTGATAGCAGCGACAGCAGTGATAGCAGTGACAGCAGCGACAGCAGCGATAGCAGCGACAGCAGTGATAG------------------TAGTGATAGCAG-
HAP33     AGCAGCGACAGCAGTGACAGCAGCGACAGCAGTGACAGCAGCGATAGCAGCGACAGCAGCG-----ACAGCAGCGATAGCAGTGACAGCAGCAATAGCAGTGATAGCAGCGACAGCAGTGATAGCAGTGACAGCAGCGACAGCAGCGATAGCAGCGACAGCAGTGATAG------------------TAGTGATAGCAGT
HAP35     AGCAGCGACAGCAGTGACAGCAGCGACAGCAGTGACAGCAGCGATAGCAGCGACAGCAGCG-----ACAGCAGCGATAGCAGTGACAGCAGCAATAGCAGTGATAGCAGCGACAGCAGTGATAGCAGTGACAGCAGCGACAGCAGCGATAGCAGCGACAGCAGTGATAG------------------TAGTGATAGCAGT
MERGED    AGCAGCGACAGCAGTGACAGCAGCGACAGCAGTGACAGCAGCGATAGCAGCGACAGCAGCG-----ACAGCAGCGATAGCAGTGACAGCAGCAATAGCAGTGATAGCAGCGACAGCAGTGATAGCAGTGACAGCAGCGACAGCAGCGATAGCAGCGACAGCAGCGATAGCAGCGACAGCAGCGATAGTAGTGATAGCAGT
TRANSL     S  S D/N S  S  D  S  S  D  S  S  D  S  S  D  S  S  D  S  S        D  S  S  D  S  S  D  S  S  N  S  S  D  S  S  D  S  S  D  S  S  D  S  S  D  S  S  D  S  S  D  S  S  D  S  S  D  S  S  D  S  S  D  S  S  D  S  S  1208
```

Notes:

Snp8: c.3454G>A; p.D1152N

ID24: NM_014208.3:c.3466_3492delAGCAGCGACAGCAGTGACAGCAGCGAT

ID25: NM_014208.3:c.3509_3517delACAGCAGCG.

ID26: NM_014208.3:c.3591_3599delTAGCAGCGA.

ID27: NM_014208.3:c.3594_3611dupCAGCGACAGCAGCGATAG.

Mutations: 21. g.12595delG; c.3461delG; p.(Ser1154Metfs*160) [This paper, Family 11]

22. g.12614_12615insCTGCT; c.3480_3481insCTGCT; p.(Asp1161Leufs*155) [8]

23. g.12638_12642dupCAGCG; c.3504_3508dupCAGCG; p.(Asp1170Alafs*146) [1]
24. g.12643_12655del13; c.3509_3521delACAGCAGCGATAG; p.(Asp1170Alafs*140)[6]
25. g.12680_12684delinsG; c.3546_3550delTAGCAinsG; p.(Asp1182Glufs*131)[4]
26. g.12694delG; c.3560delG; p.(Ser1187Metfs*127) [9]
27. g.12716_12725del; c.3582_3591delCAGCAGCGAT; p.(Asp1194Glufs*117)[5]

```
         3625 ID28  ID29         ID30          3625-3700del76                3700                ID31                                ID32                                                                   3806
REFSEQ   GACAGCAGTGACAGCAGCGACAGCAGTGACAGCAGCGACAGCAGTGACAGCAGCGACAGCAGTGACAGCAATGAAAGCAGCGACAGCAGTGACAGCAGCGATAGCAGTGACAGCAGC------------------AACAGCAGTGACAGCAGCGACAGCAGTGATAGCAGTGACAGCACATCTGACAGCAATGATGAGAG
Fam8a    GACAGCAGTGACAGCAGCGACAGCAGTGACAGCAGCGACAGCAGTGACAGCAGCGACAGCAGTGACAGCAATGAAAGCAGCGACAGCAGTGACAGCAGCGATAGCAGTGACAGCAGC------------------AACAGCAGTGACAGCAGCGACAGCAGTGATAGCAGTGACAGCACATCTGACAGCAATGATGAGAG
Fam8b    GACAGCAGTGACAGCAGCGACAGCAGTGACAGCAGCGACAGCAGTGACAGCAGCGACAGCAGTGACAGCAATGAAAGCAGCGACAGCAGTGACAGCAGCGATAGCAGTGACAGCAGC------------------AACAGCAGTGACAGCAGCGACAGCAGTGATAGCAGTGACAGCACATCTGACAGCAATGATGAGAG
Fam9a    GACAGCAGTGACAGCAGCGACAGCAGTGACAGCAGCGACAGCAGTGACAGCAGCGACAGCAGTGACAGCAATGAAAGCAGCGACAGCAGTGACAGCAGCGATAGCAGTGACAGCAGC------------------AACAGCAGTGACAGCAGCGACAGCAGTGATAGCAGTGACAGCACATCTGACAGCAATGATGAGAG
Fam9b    GACAGCAGTGACAGCAGCGACAGCAGTGACAGCAGCGACAGCAGTGACAGCAGCGACAGCAGTGACAGCAATGAAAGCAGCGACAGCAGTGACAGCAGCGATAGCAGTGACAGCAGC------------------AACAGCAGTGACAGCAGCGACAGCAGTGATAGCAGTGACAGCACATCTGACAGCAATGATGAGAG
Fam10a   GACAGCAGTGACAGCAGCGACAGCAGTGACAGCAGCGACAGCAGTGACAGCAGCGACAGCAGTGACAGCAATGAAAGCAGCGACAGCAGTGACAGCAGCGATAGCAGTGACAGCAGC------------------AACAGCAGTGACAGCAGCGACAGCAGTGATAGCAGTGACAGCACATCTGACAGCAATGATGAGAG
Fam10b   GACAGCAGTGACAGCAGCGACAGCAGTGACAGCAGCGACAGCAGTGACAGCAGCGACAGCAGTGACAGCAATGAAAGCAGCGACAGCAGTGACAGCAGCGATAGCAGTGACAGCAGC------------------AACAGCAGTGACAGCAGCGACAGCAGTGATAGCAGTGACAGCACATCTGACAGCAATGATGAGAG
Fam11a   GACAGCAGTGACAGCAGCGACAGCAGTGACAGCAGCGACAGCAGTGACAGCAGCGACAGCAGTGACAGCAATGAAAGCAGCGACAGCAGTGACAGCAGCGATAGCAGTGACAGCAGC------------------AACAGCAGTGACAGCAGCGACAGCAGTGATAGCAGTGACAACACATCTGACAGCAATGATGAGAG
Fam11b   GACAGCAGTGACAGCAGCGACAGCAGTGACAGCAGCGACAGCAGTGACAGCAGCGACAGCAGTGACAGCAATGAAAGCAGCGACAGCAGTGACAGCAGCGATAGCAGTGACAGCAGC------------------AACAGCAGTGACAGCAGCGACAGCAGTGATAGCAGTGACAGCACATCTGACAGCAATGATGAGAG
Fam12a   GACAGCAGTGACAGCAGCGACAGCAGTGACAGCAGCGACAGCAGTGACAGCAGCGACAGCAGTGACAGCAATGAA-GCAGCGACAGCAGTGACAGCAGCGATAGCAGTGACAGCAGC------------------AACAGCAGTGACAGCAGCGACAGCAGTGATAGCAGTGACAGCACATCTGACAGCAATGATGAGAG
Fam12b   GACAGCAGTGACAGCAGCGACAGCAGTGACAGCAGCGACAGCAGTGACAGCAGCGACAGCAGTGACAGCAATGAAAGCAGCGACAGCAGTGACAGCAGCGATAGCAGTGACAGCAGC------------------AACAGCAGTGACAGCAGCGACAGCAGTGATAGCAGTGACAGCACATCTGACAGCAATGATGAGAG
F1A      GACAGCAGTGACAGCAGCGACAGCAGTGACAGCAGCGACAGCAGTGACAGCAGCGACAGCAGTGACAGCAATGAAAGCAGCGACAGCAGTGACAGCAGCGATAGCAGTGACAGCAGC------------------AACAGCAGTGACAGCAGCGACAGCAGTGATAGCAGTGACAGCACATCTGACAGCAATGATGAGAG
F1B      GACAGCAGTGACAGCAGCGACAGCAGTGACAGCAGCGACAGCAGTGACAGCAGCGACAGCAGTGACAGCAATGAAAGCAGCGACAGCAGTGACAGCAGCGATAGCAGTGACAGCAGC------------------AACAGCAGTGACAGCAGCGACAGCAGTGATAGCAGTGACAGCACATCTGACAGCAATGATGAGAG
F2A      GACAGCAGTGACAGCAGCGACAGCAGTGACAGCAGCGACAGCAGTGACAGCAGCGACAGCAGTGACAGCAATGAAAGCAGCGACAGCAGTGACAGCAGCGATAGCAGTGACAGCAGC------------------AACAGCAGTGACAGCAGCGACAGCAGTGATAGCAGTGACAGCACATCTGACAGCAATGATGAGAG
F2B      GACAGCAGTGACAGCAGCGACAGCAGTGACAGCAGCGACAGCAGTGACAGCAGCGACAGCAGTGACAGCAATGAAAGCAGCGACAGCAGTGACAGCAGCGATAGCAGTGACAGCAGC------------------AACAGCAGTGACAGCAGCGACAGCAGTGATAGCAGTGACAGCACATCTGACAGCAATGATGAGAG
F3A      GACAGCAGTGACAGCAGCGACAGCAGTGACAGCAGCGACAGCAGTGACAGCAGCGACAGCAGTGACAGCAATGAAAGCAGCGACAGCAGTGACAGCAGCGATAGCAGTGACAGCAGC------------------AACAGCAGTGACAGCAGCGACAGCAGTGATAGCAGTGACAGCACATCTGACAGCAATGATGAGAG
F3B      GACAGCAGTGACAGCAGCGACAGCAGTGACAGCAGCGACAGCAGTGACAGCAGCGACAGCAGTGACAGCAATGAAAGCAGCGACAGCAGTGACAGCAGCGATAGCAGTGACAGCAGC------------------AACAGCAGTGACAGCAGCGACAGCAGTGATAGCAGTGACAGCACATCTGACAGCAATGATGAGAG
F4A      GACAGCAGTGACAGCAGCGACAGCAGTGACAGCAGCGACAGCAGTGACAGCAGCGACAGCAGTGACAGCAATGAAAGCAGCGACAGCAGTGACAGCAGCGATAGCAGTGACAGCAGC------------------AACAGCAGTGACAGCAGCGACAGCAGTGATAGCAGTGACAGCACATCTGACAGCAATGATGAGAG
F4B      GACAGCAGTGACAGCAGCGACAGCAGTGACAGCAGCGACAGCAGTGACAGCAGCGACAGCAGTGACAGCAATGAAAGCAGCGACAGCAGTGACAGCAGCGATAGCAGTGACAGCAGC------------------AACAGCAGTGACAGCAGCGACAGCAGTGATAGCAGTGACAGCACATCTGACAGCAATGATGAGAG
F5A      GACAGCAGTGACAGCAGCGACAGCAGTGACAGCAGCGACAGCAGTGACAGCAGCGACAGCAGTGACAGCAATGAAAGCAGCGACAGCAGTGACAGCAGCGATAGCAGTGACAGCAGC------------------AACAGCAGTGACAGCAGCGACAGCAGTGATAGCAGTGACAGCACATCTGACAGCAATGATGAGAG
F5B      GACAGCAGTGACAGCAGCGACAGCAGTGACAGCAGCGACAGCAGTGACAGCAGCGACAGCAGTGACAGCAATGAAAGCAGCGACAGCAGTGACAGCAGCGATAGCAGTGACAGCAGC------------------AACAGCAGTGACAGCAGCGACAGCAGTGATAGCAGTGACAGCACATCTGACAGCAATGATGAGAG
HAP1A    GACAGCAGTGACAGCAGCGACAGCAGTGACAGCAGCGACAGCAGTGACAGCAGCGACAGCAGTGACAGCAATGAAAGCAGCGACAGCAGTGACAGCAGCGATAGCAGTGACAGCAGC------------------AACAGCAGTGACAGCAGCGACAGCAGTGATAGCAGTGACAGCACATCTGACAGCAATGATGAGAG
HAP2A    GACAGCAGTGACAGCAGCGACAGCAGTGACAGCAGCGACAGCAGTGACAGCAGCGACAGCAGTGACAGCAATGAAAGCAGCGACAGCAGTGACAGCAGCGATAGCAGTGACAGCAGC------------------AACAGCAGTGACAGCAGCGACAGCAGTGATAGCAGTGACAGCACATCTGACAGCAATGATGAGAG
HAP3A    GACAGCAGTGACAGCAGCGACAGCAGTGACAGCAGCGACAGCAGTGACAGCAGCGACAGCAGTGACAGCAATGAAAGCAGCGACAGCAGTGACAGCAGCGATAGCAGTGACAGCAGC------------------AACAGCAGTGACAGCAGCGACAGCAGTGATAGCAGTGACAGCACATCTGACAGCAATGATGAGAG
HAP15A   GACAGCAGTGACAGCAGCGACAGCAGTGACAGCAGCGACAGCAGTGACAGCAGCGACAGCAGTGACAGCAATGAAAGCAGCGACAGCAGTGACAGCAGCGATAGCAGTGACAGCAGC------------------AACAGCAGTGACAGCAGCGACAGCAGTGATAGCAGTGACAGCACATCTGACAGCAATGATGAGAG
HAP17B   GACAGCAGTGACAGCAGCGACAGCAGTGACAGCAGCGACAGCAGTGACAGCAGCGACAGCAGTGACAGCAATGAAAGCAGCGACAGCAGTGACAGCAGCGATAGCAGTGACAGCAGC------------------AACAGCAGTGACAGCAGCGACAGCAGTGATAGCAGTGACAGCACATCTGACAGCAATGATGAGAG
HAP20A   -----------------CGACAGCAGTGACAGCAGCGACAGCAGTGACAGCAGCGACAGCAGTGACAGCAATGAAAGCAGCGACAGCAGTGACAGCAGCGATAGCAGTGACAGCAGCGATAGCAGTGACAGCAGCAACAGCAGTGACAGCAGCGACAGCAGTGATAGCAGTGACAGCACATCTGACAGCAATGATGAGAG
HAP20B   GACAGCAGTGACAGCAGCGACAGCAGTGACAGCAGCGACAGCAGTGACAGCAGCGACAGCAGTGACAGCAATGAAAGCAGCGACAGCAGTGACAGCAGCGATAGCAGTGACAGCAGC------------------AACAGCAGTGACAGCAGCGACAGCAGTGATAGCAGTGACAGCACATCTGACAGCAATGATGAGAG
HAP36A   -----------------CGACAGCAGTGACAGCAGCGACAGCAGTGACAGCAGCGACAGCAGTGACAGCAATGAAAGCAGCGACAGCAGTGACAGCAGCGATAGCAGTGACAGCAGCGATAGCAGTGACAGCAGCAACAGCAGTGACAGCAGCGACAGCAGTGATAGCAGTGACAGCACATCTGACAGCAATGATGAGAG
HAP37A   GACAGCAGTGACAGCAGCGACAGCAGTGACAGCAGCGACAGCAGTGACAGCAGCGACAGCAGTGACAGCAATGAAAGCAGCGACAGCAGTGACAGCAGCGATAGCAGTGACAGCAGC------------------AACAGCAGTGACAGCAGCGACAGCAGTGATAGCAGTGACAGCACATCTGACAGCAATGATGAGAG
HAP38A   -----------------------------------CGACAGCAGTGACAGCAGCGACAGCAGTGACAGCAATGAAAGCAGCGACAGCAGTGACAGCAGCGATAGCAGTGACAGCAGCGATAGCAGTGACAGCAGCAACAGCAGTGACAGCAGCGACAGCAGTGATAGCAGTGACAGCACATCTGACAGCAATGATGAGAG
SHAP1:   GACAGCAGTGACAGCAGCGACAGCAGTGACAGCAGCGACAGCAGTGACAGCAGCGACAGCAGTGACAGCAATGAAAGCAGCGACAGCAGTGACAGCAGCGATAGCAGTGACAGCAGC------------------AACAGCAGTGACAGCAGCGACAGCAGTGATAGCAGTGACAGCACATCTGACAGCAATGATGAGAG
SHAP2:   GACAGCAGTGACAGCAGCGACAGCAGTGACAGCAGCGACAGCAGTGACAGCAGCGACAGCAGTGACAGCAATGAAAGCAGCGACAGCAGTGACAGCAGCGATAGCAGTGACAGCAGC------------------AACAGCAGTGACAGCAGCGACAGCAGTGATAGCAGTGACAGCACATCTGACAGCAATGATGAGAG
SHAP3:   GACAGCAGTGACAGCAGCGACAGCAGTGACAGCAGCGACAGCAGTGACAGCAGCGACAGCAGTGACAGCAATGAAAGCAGCGACAGCAGTGACAGCAGCGATAGCAGTGACAGCAGC------------------AACAGCAGTGACAGCAGCGACAGCAGTGATAGCAGTGACAGCACATCTGACAGCAATGATGAGAG
SHAP4:   GACAGCAGTGACAGCAGCGACAGCAGTGACAGCAGCGACAGCAGTGACAGCAGCGACAGCAGTGACAGCAATGAAAGCAGCGACAGCAGTGACAGCAGCGATAGCAGTGACAGCAGC------------------AACAGCAGTGACAGCAGCGACAGCAGTGATAGCAGTGACAGCACATCTGACAGCAATGATGAGAG
SHAP5:   -----------------TGACAGCAGTGACAGCAGCGACAGCAGTGACAGCAGCGACAGCAGTGACAGCAATGAAAGCAGCGACAGCAGTGACAGCAGCGATAGCAGTGACAGCAGC------------------AACAGCAGTGACAGCAGCGACAGCAGTGATAGCAGTGACAGCACATCTGACAGCAATGATGAGAG
SHAP6:   GACAGCAGTGACAGCAGCGACAGCAGTGACAGCAGCGACAGCAGTGACAGCAGCGACAGCAGTGACAGCAATGAAAGCAGCGACAGCAGTGACAGCAGCGATAGCAGTGACAGCAGC------------------AACAGCAGTGACAGCAGCGACAGCAGTGATAGCAGTGACAGCACATCTGACAGCAATGATGAGAG
SHAP6(2) GACAGCAGTGACAGCAGCGACAGCAGTGACAGCAGCGACAGCAGTGACAGCAGCGACAGCAGTGACAGCAATGAAAGCAGCGACAGCAGTGACAGCAGCGATAGCAGTGACAGCAGC------------------AACAGCAGTGACAGCAGCGACAGCAGTGATAGCAGTGACAGCACATCTGACAGCAATGATGAGAG
SHAP7    GACAGCAGTGACAGCAGCGACAGCAGTGACAGCAGCGACAGCAGTGACAGCAGCGACAGCAGTGACAGCAATGAAAGCAGCGACAGCAGTGACAGCAGCGATAGCAGTGACAGCAGC------------------AACAGCAGTGACAGCAGCGACAGCAGTGATAGCAGTGACAGCACATCTGACAGCAATGATGAGAG
SHAP102  -----------------CGACAGCAGTGACAGCAGCGACAGCAGTGACAGCAGCGACAGCAGTGACAGCAATGAAAGCAGCGACAGCAGTGACAGCAGCGATAGCAGTGACAGCAGCGATAGCAGTGACAGCAGCAACAGCAGTGACAGCAGCGACAGCAGTGATAGCAGTGACAGCACATCTGACAGCAATGATGAGAG
SHAP130  GACAGCAGTGACAGCAGCGACAGCAGTGACAGCAGCGACAGCAGTGACAGCAGCGACAGCAGTGACAGCAATGAAAGCAGCGACAGCAGTGACAGCAGCGATAGCAGTGACAGCAGC------------------AACAGCAGTGACAGCAGCGACAGCAGTGATAGCAGTGACAGCACATCTGACAGCAATGATGAGAG
SHAP106  GACAGCAGTGACAGCAGCGACAGCAGTGACAGCAGCGACAGCAGTGACAGCAGCGACAGCAGTGACAGCAATGAAAGCAGCGACAGCAGTGACAGCAGCGATAGCAGTGACAGCAGC------------------AACAGCAGTGACAGCAGCGACAGCAGTGATAGCAGTGACAGCACATCTGACAGCAATGATGAGAG
SHAP72   GACAGCAGTGACAGCAGCGACAGCAGTGACAGCAGCGACAGCAGTGACAGCAGCGACAGCAGTGACAGCAATGAAAGCAGCGACAGCAGTGACAGCAGCGATAGCAGTGACAGCAGC------------------AACAGCAGTGACAGCAGCGACAGCAGTGATAGCAGTGACAGCACATCTGACAGCAATGATGAGAG
SHAP110  -----------------TGACAGCAGTGACAGCAGCGACAGCAGTGACAGCAGCGACAGCAGTGACAGCAATGAAAGCAGCGACAGCAGTGACAGCAGCGATAGCAGTGACAGCAGC------------------AACAGCAGTGACAGCAGCGACAGCAGTGATAGCAGTGACAGCACATCTGACAGCAATGATGAGAG
HAP4     -----------------CGACAGCAGTGACAGCAGCGACAGCAGTGACAGCAGCGACAGCAGTGACAGCAATGAAAGCAGCGACAGCAGTGACAGCAGCGATAGCAGTGACAGCAGCGATAGCAGTGACAGCAGCAACAGCAGTGACAGCAGCGACAGCAGTGATAGCAGTGACAGCACATCTGACAGCAATGATGAGAG
HAP5     -----------------CGACAGCAGTGACAGCAGCGACAGCAGTGACAGCAGCGACAGCAGTGACAGCAATGAAAGCAGCGACAGCAGTGACAGCAGCGATAGCAGTGACAGCAGCGATAGCAGTGACAGCAGCAACAGCAGTGACAGCAGCGACAGCAGTGATAGCAGTGACAGCACATCTGACAGCAATGATGAGAG
HAP8     -----------------TGACAGCAGTGACAGCAGCGACAGCAGTGACAGCAGCGACAGCAGTGACAGCAATGAAAGCAGCGACAGCAGTGACAGCAGCGATAGCAGTGACAGCAGC------------------AACAGCAGTGACAGCAGCGACAGCAGTGATAGCAGTGACAGCACATCTGACAGCAATGATGAGAG
HAP13    GACAGCAG---------TGACAGCAGTGACAGCAGCGACAGCAGTGACAGCAGCGACAGCAGTGGCAGCAATGAAAGCAGCGACAGCAGTGACAGCAGCGATAGCAGTGACAGCAGC------------------AACAGCAGTGACAGCAGCGACAGCAGTGATAGCAGTGACAGCACATCTGACAGCAATGATGAGAG
HAP24    GACAGCAGTGACAGCAGCGACAGCAGTGACAGCAGCGACAGCAGTGACAGCAGCGACAGCAGTGACAGCAATGAAAGCAGCGACAGCAGTGACAGCAGCGATAGCAGTGACAGCAGC------------------AACAGCAGTGACAGCAGCGACAGCAGTGATAGCAGTGACAGCACATCTGACAGCAATGATGAGAG
HAP25    GACAGCAGTGACAGCAGCGACAGCAGTGACAGCAGCAACAGCAGTGACAGCAGCGACAGCAGTGACAGCAATGAAAGCAGCGACAGCAGTGACAGCAGCGATAGCAGTGACAGCAGC------------------AACAGCAGTGACAGCAGCGACAGCAGTGATAGCAGTGACAGCACATCTGACAGCAATGATGAGAG
HAP26    GACAGCAG---------TGACAGCAGTGACAGCAGCGACAGCAGTGACAGCAGCGACAGCAGTGACAGCAATGAAAGCAGCGACAGCAGTGACAGCAGCGATAGCAGTGACAGCAGC------------------AACAGCAGTGACAGCAGCGACAGCAGTGATAGCAGTGACAGCACATCTGACAGCAATGATGAGAG
HAP29    GACAGCAGTGACAGCAGCGACAGCAGTGACAGCAGCGACAGCAGTGACAGCAGCGACAGCAGTGACAGCAATGAAAGCAGCGACAGCAGTGACAGCAGCGATAGCAGTGACAGCAGC------------------AACAGCAGTGACAGCAGCGACAGCAGTGATAGCAGTGACAGCACATCTGACAGCAATGATGAGAG
HAP32    -----------------CGACAGCAGTGACAGCAGCGACAGCAGTGACAGCAGCGACAGCAGTGACAGCAATGAAAGCAGCGACAGCAGTGACAGCAGCGATAGCAGTGACAGCAGCGATAGCAGTGACAGCAGCAACAGCAGTGACAGCAGCGACAGCAGTGATAGCAGTGACAGCACATCTGACAGCAATGATGAGAG
HAP33    GACAGCAGTGACAGCAGCGACAGCAGTGA---------------------------------------------------------------------------------------------------------------------CAGCAGCGACAGCAGTGATAGCAGTGACAGCACATCTGACAGCAATGATGAGAG
HAP35    GACAGCAGTGACAGCAGCGACAGCAGTGACAGCAGCGACAGCAGTGACAGCAGCGACAGCAGTGACAGCAATGAAAGCAGCGACAGCAGTGACAGCAGCGATAGCAGTGACAGCAGC------------------AACAGCAGTGACAGCAGCGACAGCAGTGATAGCAGTGACAGCACATCTGACAGCAATGATGAGAG
MERGED   GACAGCAGTGACAGCAGCGACAGCAGTGACAGCAGCGACAGCAGTGACAGCAGCGACAGCAGTGACAGCAATGAAAGCAGCGACAGCAGTGACAGCAGCGATAGCAGTGACAGCAGCGATAGCAGTGACAGCAGCAACAGCAGTGACAGCAGCGACAGCAGTGATAGCAGTGACAGCACATCTGACAGCAATGATGAGAG
TRANSL    D  S  S  D  S  S  D  S  S  D  S  S  D  S  S  D  S  S  D  S  S  D  S  N  E  S  S  D  S  S  D  S  S  D  S  S  D  S  S  D  S  S  D  S  S  N  S  S  D  S  S  D  S  S  D  S  S  D  S  T  S  D  S  N  D  E  S
```

Notes:

ID28: NM_014208.3:c.3624_3641delTGACAGCAGTGACAGCAG.

ID29: NM_014208.3:c.3633_3641delTGACAGCAG.

ID30: NM_014208.3:c.3624_3659delTGACAGCAGTGACAGCAGCGACAGCAGTGACAGCAG

ID31: NM_014208.3:c.3654_3752delCAGCAGCGACAGCAGTGACAGCAGCGACAGCAGTGACAGCAATGAAAGCAG CGACAGCAGTGACAGCAGCGATAGCAGTGACAGCAGCAACAGCAGTGA.

ID32: NM_014208.3:c.3724_3741dup (same as: NM_014208.3:c.3741_3742insGATAGCAGTGACAGCAGC).

Mutations:

28. g.12810delA; c.3676delA; p.(Ser1226Alafs*88) [11]
29. g.12759_12834del76; c.3625-3700del76; p.(Asp1209Alafs*80) [5]
30. g.12834delA; c.3700del**A**; p.(Ser1234Alafs*80); [This paper, Family 12]

```
      3807                                                                                           3906
REFSEQ    TGACAGCCAGAGCAAGTCTGGTAACGGTAACAACAATGGAAGTGACAGTGACAGTGACAGTGAAGGCAGTGACAGTAACCACTCAACCAGTGATGATTAG
Fam8a     TGACAGCCAGAGCAAGTCTGGTAACGGTAACAACAATGGAAGTGACAGTGACAGTGACAGTGAAGGCAGTGACAGTAACCACTCAACCAGTGATGA
Fam8b     TGACAGCCAGAGCAAGTCTGGTAACGGTAACAACAATGGAAGTGACAGTGACAGTGACAGTGAAGGCAGTGACAGTAACCACTCAACCAGTGATGA
Fam9a     TGACAGCCAGAGCAAGTCTGGTAACGGTAACAACAATGGAAGTGACAGTGACAGTGACAGTGAAGGCAGTGACAGTAACCACTCAACCAGTGATGA
Fam9b     TGACAGCCAGAGCAAGTCTGGTAACGGTAACAACAATGGAAGTGACAGTGACAGTGACAGTGAAGGCAGTGACAGTAACCACTCAACCAGTGATGA
Fam10a    TGACAGGCAGAGCAAGTCTGGTAACGGTAACAACAATGGAAGTGACAGTGACAGTGACAGTGAAGGCAGTGACAGTAACCACTCAACCAGTGATGA
Fam10b    TGACAGCCAGAGCAAGTCTGGTAACGGTAACAACAATGGAAGTGACAGTGACAGTGACAGTGAAGGCAGTGACAGTAACCACTCAACCAGTGATGA
Fam11a    TGACAGCCAGAGCAAGTCTGGTAACGGTAACAACAATGGAAGTGACAGTGACAGTGACAGTGAAGGCAGTGACAGTAACCACTCAACCAGTGATGA
Fam11b    TGACAGCCAGAGCAAGTCTGGTAACGGTAACAACAATGGAAGTGACAGTGACAGTGACAGTGAAGGCAGTGACAGTAACCACTCAACCAGTGATG
Fam12a    TGACAGCCAGAGCAAGTCTGGTAACGGTAACAACAATGGAAGTGACAGTGACAGTGACAGTGAAGGCAGTGACAGTAACCACTCAACCAGTGATGA
Fam12b    TGACAGCCAGAGCAAGTCTGGTAACGGTAACAACAATGGAAGTGACAGTGACAGTGACAGTGAAGGCAGTGACAGTAACCACTCAACCAGTGATGA
F1A       TGACAGCCAGAGCAAGTCTGGTAACGGTAACAACAATGGAAGTGACAGTGACAGTGACAGTGAAGGCAGTGACAGTAACCACTCAACCAGTGATGATTAG
F1B       TGACAGCCAGAGCAAGTCTGGTAACGGTAACAACAATGGAAGTGACAGTGACAGTGACAGTGAAGGCAGTGACAGTAACCACTCAACCAGTGATGATTAG
F2A       TGACAGCCAGAGCAAGTCTGGTAACGGTAACAACAATGGAAGTGACAGTGACAGTGACAGTGAAGGCAGTGACAGTAACCACTCAACCAGTGATGATTAG
F2B       TGACAGCCAGAGCAAGTCTGGTAACGGTAACAACAATGGAAGTGACAGTGACAGTGACAGTGAAGGCAGTGACAGTAACCACTCAACCAGTGATGATTAG
F3A       TGACAGCCAGAGCAAGTCTGGTAACGGTAACAACAATGGAAGTGACAGTGACAGTGACAGTGAAGGCAGTGACAGTAACCACTCAACCAGTGATGATTAG
F3B       TGACAGCCAGAGCAAGTCTGGTAACGGTAACAACAATGGAAGTGACAGTGACAGTGACAGTGAAGGCAGTGACAGTAACCACTCAACCAGTGATGATTAG
F4A       TGACAGCCAGAGCAAGTCTGGTAACGGTAACAACAATGGAAGTGACAGTGACAGTGACAGTGAAGGCAGTGACAGTAACCACTCAACCAGTGATGATTAG
F4B       TGACAGCCAGAGCAAGTCTGGTAACGGTAACAACAATGGAAGTGACAGTGACAGTGACAGTGAAGGCAGTGACAGTAACCACTCAACCAGTGATGATTAG
F5A       TGACAGCCAGAGCAAGTCTGGTAACGGTAACAACAATGGAAGTGACAGTGACAGTGACAGTGAAGGCAGTGACAGTAACCACTCAACCAGTGATGATTAG
F5B       TGACAGCCAGAGCAAGTCTGGTAACGGTAACAACAATGGAAGTGACAGTGACAGTGACAGTGAAGGCAGTGACAGTAACCACTCAACCAGTGATGATTAG
HAP1A     TGACAGCCAGAGCAAGTCTGGTAACGGTAACAACAATGGAAGTGACAGTGACAGTGACAGTGAAGGCAGTGACAGTAACCACTCAACCAGTGATGATTAG
HAP2A     TGACAGCCAGAGCAAGTCTGGTAACGGTAACAACAATGGAAGTGACAGTGACAGTGACAGTGAAGGCAGTGACAGTAACCACTCAACCAGTGATGATTAG
HAP3A     TGACAGCCAGAGCAAGTCTGGTAACGGTAACAACAATGGAAGTGACAGTGACAGTGACAGTGAAGGCAGTGACAGTAACCACTCAACCAGTGATGATTAG
HAP15A    TGACAGCCAGAGCAAGTCTGGTAACGGTAACAACAATGGAAGTGACAGTGACAGTGACAGTGAAGGCAGTGACAGTAACCACTCAACCAGTGATGATTAG
HAP17B    TGACAGCCAGAGCAAGTCTGGTAACGGTAACAACAATGGAAGTGACAGTGACAGTGACAGTGAAGGCAGTGACAGTAACCACTCAACCAGTGATGATTAG
HAP20A    TGACAGCCAGAGCAAGTCTGGTAACGGTAACAACAATGGAAGTGACAGTGACAGTGACAGTGAAGGCAGTGACAGTAACCACTCAACCAGTGATGATTAG
HAP20B    TGACAGCCAGAGCAAGTCTGGTAACGGTAACAACAATGGAAGTGACAGTGACAGTGACAGTGAAGGCAGTGACAGTAACCACTCAACCAGTGATGATTAG
HAP36A    TGACAGCCAGAGCAAGTCTGGTAACGGTAACAACAATGGAAGTGACAGTGACAGTGACAGTGAAGGCAGTGACAGTAACCACTCAACCAGTGATGATTAG
HAP37A    TGACAGCCAGAGCAAGTCTGGTAACGGTAACAACAATGGAAGTGACAGTGACAGTGACAGTGAAGGCAGTGACAGTAACCACTCAAGCAGTGATGATTAG
HAP38A    TGACAGCCAGAGCAAGTCTGGTAACGGTAACAACAATGGAAGTGACAGTGACAGTGACAGTGAAGGCAGTGACAGTAACCACTCAACCAGTGATGATTAG
SHAP1:    TGACAGCCAGAGCAAGTCTGGTAACGGTAACAACAATGGAAGTGACAGTGACAGTGACAGTGAAGGCAGTGACAGTAACCACTCAACCAGTGATGATTAG
SHAP2:    TGACAGCCAGAGCAAGTCTGGTAACGGTAACAACAATGGAAGTGACAGTGACAGTGACAGTGAAGGCAGTGACAGTAACCACTCAACCAGTGATGATTAG
SHAP3:    TGACAGCCAGAGCAAGTCTGGTAACGGTAACAACAATGGAAGTGACAGTGACAGTGACAGTGAAGGCAGTGACAGTAACCACTCAACCAGTGATGATTAG
SHAP4:    TGACAGCCAGAGCAAGTCTGGTAACGGTAACAACAATGGAAGTGACAGTGACAGTGACAGTGAAGGCAGTGACAGTAACCACTCAACCAGTGATGATTAG
SHAP5:    TGACAGCCAGAGCAAGTCTGGTAACGGTAACAACAATGGAAGTGACAGTGACAGTGACAGTGAAGGCAGTGACAGTAACCACTCAACCAGTGATGATTAG
SHAP6:    TGACAGCCAGAGCAAGTCTGGTAACGGTAACAACAATGGAAGTGACAGTGACAGTGACAGTGAAGGCAGTGACAGTAACCACTCAACCAGTGATGATTAG
SHAP6(2)  TGACAGCCAGAGCAAGTCTGGTAACGGTAACAACAATGGAAGTGACAGTGACAGTGACAGTGAAGGCAGTGACAGTAACCACTCAACCAGTGATGATTAG
SHAP7     TGACAGCCAGAGCAAGTCTGGTAACGGTAACAACAATGGAAGTGACAGTGACAGTGACAGTGAAGGCAGTGACAGTAACCACTCAACCAGTGATGATTAG
SHAP102   TGACAGCCAGAGCAAGTCTGGTAACGGTAACAACAATGGAAGTGACAGTGACAGTGACAGTGAAGGCAGTGACAGTAACCACTCAACCAGTGATGATTAG
SHAP130   TGACAGCCAGAGCAAGTCTGGTAACGGTAACAACAATGGAAGTGACAGTGACAGTGACAGTGAAGGCAGTGACAGTAACCACTCAACCAGTGATGATTAG
SHAP106   TGACAGCCAGAGCAAGTCTGGTAACGGTAACAACAATGGAAGTGACAGTGACAGTGACAGTGAAGGCAGTGACAGTAACCACTCAACCAGTGATGATTAG
SHAP72    TGACAGCCAGAGCAAGTCTGGTAACGGTAACAACAATGGAAGTGACAGTGACAGTGACAGTGAAGGCAGTGACAGTAACCACTCAACCAGTGATGATTAG
SHAP110   TGACAGCCAGAGCAAGTCTGGTAACGGTAACAACAATGGAAGTGACAGTGACAGTGACAGTGAAGGCAGTGACAGTAACCACTCAACCAGTGATGATTAG
HAP4      TGACAGCCAGAGCAAGTCTGGTAACGGTAACAACAATGGAAGTGACAGTGACAGTGACAGTGAAGGCAGTGACAGTAACCACTCAACCAGTGATGATTAG
HAP5      TGACAGCCAGAGCAAGTCTGGTAACGGTAACAACAATGGAAGTGACAGTGACAGTGACAGTGAAGGCAGTGACAGTAACCACTCAACCAGTGATGATTAG
HAP8      TGACAGCCAGAGCAAGTCTGGTAACGGTAACAACAATGGAAGTGACAGTGACAGTGACAGTGAAGGCAGTGACAGTAACCACTCAACCAGTGATGATTAG
HAP13     TGACAGCCAGAGCAAGTCTGGTAACGGTAACAACAATGGAAGTGACAGTGACAGTGACAGTGAAGGCAGTGACAGTAACCACTCAACCAGTGATGATTAG
HAP24     TGACAGCCAGAGCAAGTCTGGTAACGGTAACAACAATGGAAGTGACAGTGACAGTGACAGTGAAGGCAGTGACAGTAACCACTCAACCAGTGATGATTAG
HAP25     TGACAGCCAGAGCAAGTCTGGTAACGGTAACAACAATGGAAGTGACAGTGACAGTGACAGTGAAGGCAGTGACAGTAACCACTCAACCAGTGATGATTAG
HAP26     TGACAGCCAGAGCAAGTCTGGTAACGGTAACAACAATGGAAGTGACAGTGACAGTGACAGTGAAGGCAGTGACAGTAACCACTCAACCAGTGATGATTAG
HAP29     TGACAGCCAGAGCAAGTCTGGTAACGGTAACAACAATGGAAGTGACAGTGACAGTGACAGTGAAGGCAGTGACAGTAACCACTCAACCAGTGATGATTAG
HAP32     TGACAGCCAGAGCAAGTCTGGTAACGGTAACAACAATGGAAGTGACAGTGACAGTGACAGTGAAGGCAGTGACAGTAACCACTCAACCAGTGATGATTAG
HAP33     TGACAGCCAGAGCAAGTCTGGTAACGGTAACAACAATGGAAGTGACAGTGACAGTGACAGTGAAGGCAGTGACAGTAACCACTCAACCAGTGATGATTAG
HAP35     TGACAGCCAGAGCAAGTCTGGTAACGGTAACAACAATGGAAGTGACAGTGACAGTGACAGTGAAGGCAGTGACAGTAACCACTCAACCAGTGATGATTAG
MERGED    TGACAGCCAGAGCAAGTCTGGTAACGGTAACAACAATGGAAGTGACAGTGACAGTGACAGTGAAGGCAGTGACAGTAACCACTCAACCAGTGATGATTAG
TRANSL      D  S  Q  S  K  S  G  N  G  N  N  N  G  S  D  S  D  S  D  S  E  G  S  D  S  N  H  S  T  S  D  D  *
```

Mutations: No disease-causing frameshifts have been observed in this region.

References

1. Yang, J.; Kawasaki, K.; Lee, M.; Reid, B.M.; Nunez, S.M.; Choi, M.; Seymen, F.; Koruyucu, M.; Kasimoglu, Y.; Estrella-Yuson, N.; et al. The dentin phosphoprotein repeat region and inherited defects of dentin. *Mol Genet Genomic Med* **2016**, *4*, 28-38.

2. McKnight, D.A.; Suzanne Hart, P.; Hart, T.C.; Hartsfield, J.K.; Wilson, A.; Wright, J.T.; Fisher, L.W. A comprehensive analysis of normal variation and disease-causing mutations in the human DSPP gene. *Hum Mutat* **2008**, *29*, 1392-1404.

3. McKnight, D.A.; Simmer, J.P.; Hart, P.S.; Hart, T.C.; Fisher, L.W. Overlapping DSPP mutations cause dentin dysplasia and dentinogenesis imperfecta. *J Dent Res.* **2008**, *87*, 1108-1111.

4. Song, Y.L.; Wang, C.N.; Fan, M.W.; Su, B.; Bian, Z. Dentin phosphoprotein frameshift mutations in hereditary dentin disorders and their variation patterns in normal human population. *J Med Genet* **2008**, *45*, 457-464.

5. Nieminen, P.; Papagiannoulis-Lascarides, L.; Waltimo-Siren, J.; Ollila, P.; Karjalainen, S.; Arte, S.; Veerkamp, J.; Tallon Walton, V.; Chimenos Küstner, E.; Siltanen, T.; et al. Frameshift mutations in dentin phosphoprotein and dependence of dentin disease phenotype on mutation location. *J Bone Miner Res* **2011**, *26*, 873-880.

6. Li, F.; Liu, Y.; Liu, H.; Yang, J.; Zhang, F.; Feng, H. Phenotype and genotype analyses in seven families with dentinogenesis imperfecta or dentin dysplasia. *Oral Dis.* **2017**, *23*, 360-366.

7. Porntaveetus, T.; Osathanon, T.; Nowwarote, N.; Pavasant, P.; Srichomthong, C.; Suphapeetiporn, K.; Shotelersuk, V. Dental Properties, Ultrastructure, and Pulp Cells Associated With a Novel DSPP Mutation. *Oral Dis* **2018**, *24*, 619-627.

8. Lee, J.W.; Hong, J.; Seymen, F.; Kim, Y.J.; Kang, J.; Koruyucu, M.; Tuloglu, N.; Bayrak, S.; Song, J.S.; Shin, T.J.; et al. Novel frameshift mutations in DSPP cause dentin dysplasia type II. *Oral Dis* **2019**, *27*, 13182.

9. Lee, K.; Kang, H.; Lee, S.; Yoo, S.; Lee, J.; Hwang, Y.; Nam, K.; Kim, J.; Park, J.; Kim, J.-W. Novel dentin phosphoprotein frameshift mutations in dentinogenesis imperfecta type II. *Clin Genet* **2011**, *79*, 378-384.

10. McKnight, D.A.; Simmer, J.P.; Hart, P.S.; Hart, T.C.; Fisher, L.W. Overlapping DSPP mutations cause dentin dysplasia and dentinogenesis imperfecta. *J Dent Res* **2008**, *87*, 1108-1111.

11. Bloch-Zupan, A.; Huckert, M.; Stoetzel, C.; Meyer, J.; Geoffroy, V.; Razafindrakoto, R.W.; Ralison, S.N.; Randrianaivo, J.C.; Ralison, G.; Andriamasinoro, R.O.; et al. Detection of a Novel DSPP Mutation by NGS in a Population Isolate in Madagascar. *Front Physiol.* **2016**, *7*.
